# Supplementary material for: PROFIT, a PROspective, randomised placebo controlled feasibility trial of Faecal mIcrobiota Transplantation in cirrhosis: study protocol for a single-blinded trial
Source: BMJ Open. 2019 Feb 15;9(2):e023518. doi: 10.1136/bmjopen-2018-023518 (PMC6398649; doi:10.1136/bmjopen-2018-023518)
Supplement: Supplementary data [file bmjopen-2018-023518supp001.pdf]

Participant Information Sheet

Short Title: **PROFIT**

Full Title of Study: **A Prospective, randomized placebo controlled feasibility trial of faecal microbiota transplantation in cirrhosis**

Chief Investigator: **Dr D. Shawcross**

Researchers: **Dr C Woodhouse, Dr V. C. Patel, Prof A. Sanchez-Fueyo,**

Introduction

You are invited to take part in this research study. Before you decide it is important for you to understand why the research is being done and what it will involve. Please take time to read the following carefully and discuss it with friends, family or your GP if you wish. Ask us if there is anything that is not clear or if you would like more information. Take time to decide whether or not you wish to take part.

What is the purpose of the study?

Our bodies contain trillions of bacteria that live in our bowels. These bacteria help to keep us healthy. Patients with liver cirrhosis have more 'unfriendly' bacteria than people without cirrhosis. Patients with cirrhosis also have a 'leaky' gut, allowing these bacteria to leak into the blood stream, potentially causing serious infections. These can usually be treated with antibiotics, but may be very severe and may also cause kidney failure, hepatic encephalopathy (a condition where toxins build up in the blood stream, causing confusion and even coma) and potentially life threatening bleeding.

The treatment proposed in our study, FMT (faecal microbiota transplantation,) takes the bacteria from the intestines of healthy volunteers and replaces the abnormal bacteria in the gut of a patient with cirrhosis. The aim of faecal microbiota transplantation (FMT) is to replace the 'unfriendly' bacteria with 'friendly' bacteria from healthy volunteers, therefore preventing them from getting into the blood stream and causing serious problems.
Volunteers are screened very thoroughly (in a similar way to blood donors) to ensure that they are healthy and not liable to pass on any infections to the recipients of the FMT.

FMT has been tested in a small group of patients with cirrhosis in America and was found to be safe, and has also been used more widely in other conditions such as patients with a gut infection called 'clostridium difficile' and patients with inflammatory bowel disease. It is extremely successful in these patients and we would like to see if it can be as successful in liver patients.

Results from this study would support further research into the benefit of using FMT to lessen infection rates in patients with liver cirrhosis who have developed bowel problems.

What groups of patients will the study involve?

We are interested in involving people in this study who have liver cirrhosis of any cause.

Why have I been invited?

All patients who are admitted to the Liver wards and/or seen in the Liver out-patient clinics at King's College Hospital NHS Foundation Trust who have liver cirrhosis may be eligible to participate in this study. This also includes patients that are awaiting consideration for or have already been listed for liver transplantation

Do I have to take part?

No, participation is entirely voluntary. It is up to you whether or not you wish to take part. If you decide to take part you will be given this information sheet to keep and asked to sign a consent form. If you decide to take part you are still free to withdraw at any time and without giving a reason. This will not affect the medical care you will receive.

Who is organising and funding this study?

The doctor in charge of this study is Dr Debbie Shawcross. The study is funded by the National Institute of Health Research and is being sponsored by King's College London and Kings College Hospital NHS Foundation Trust.

Who has reviewed the study?

All research in the NHS is looked at by an independent group of people, called a Research Ethics Committee, to protect your interests. This study has been reviewed and approved by the London South East Research Ethics Committee. It has also been approved by the Health Research authority and the Medicines and Healthcare products Regulatory Agency.

What will happen to me if I take part (SUMMARY)?

If you decide to take part you will be randomly assigned to one of two groups. One group (the 'active treatment' group) will receive Faecal Microbiota Transplantation (FMT) whilst the other group (the comparison or 'control group') will receive a 'placebo' treatment. Administration of placebo or FMT will take place only ONCE during this study.

The placebo treatment is a 'dummy' treatment given to participants in the control group of a clinical trial. This method using a 'placebo control' is used to make sure the study results are reliable by checking that any changes measured are actually due to the effect of the active drug and not just because of chance. The placebo in this trial is a solution of saline and glycerol (salty water and a preservative, which are the same ingredients used in the FMT itself, just without the FMT.)

Both groups will then be followed up to see how effective the active treatment is based on various measurements taken. Blood, urine, saliva and stool samples will be obtained for tests at four specific times. Firstly when starting the study at 'baseline' (just before the FMT or placebo is given,) and then repeated at 7, 30 and 90 day intervals after treatment has been administered. Follow-up appointments at the 7, 30 day and 90 days after starting will be arranged to coincide as far as possible with planned routine medical appointments.

Study procedures	Screening	Baseline (-7 days)	Endoscopy	Day 7 (+/- 5days)	Day 30 (+/- 7 days)	Day 90 (+/- 7 days)
Signed informed consent	X					
Eligibility Criteria	X					
Participant demographics	X					
Medical and surgical histories	**X**			X	X	X
Dietary questionnaire		X		X	X	X
Medication usage		X		X	X	X
Clinical examination		X		X	X	X
Blood sampling		X		X	X	X
Stool sampling		X		X	X	X
Saliva sampling		X		X	X	X
Urine sampling		X		X	X	X
Randomisation		X				
FMT/placebo administration			X			
Adverse events monitoring /Safety			X	X	X	X

As part of the study you will have an endoscopy (a camera test looking into your stomach/small bowel) to give you the FMT or the placebo treatment. This means that you will not have to drink the liquid as it is placed directly into the small bowel where it is needed, avoiding the stomach acid, which could stop it working. Before the endoscopy you will be given a medicine to clear your own gut bacteria called 'Moviprep.' This will give you loose bowel motions, which is a normal reaction to the Moviprep. This is essential to clear your own gut bacteria from the bowel to allow the FMT to work.

We will see you after the endoscopy at days 7, 30 and 90 to take blood, urine, saliva and faecal samples from you. We will also assess your general wellbeing and check that your medicines have not changed. Blood samples will usually be taken at the time of when you are having tests done as part of your routine clinical care, to reduce the number of times you have to have a needle in your arm and, where possible, samples will be taken from tubes already in your blood vessels to minimise discomfort to you.
We will ask you to collect the urine and stool samples in the morning of each study visit and bring this with you when you attend clinic. We will ensure you are given all the necessary collection pots for this. After the last visit at 90 days, you will return to the usual standard of care as you were having for you underlying condition.

More detailed information about the study visits can be found on page 7 of this information sheet.

What are the side effects of taking part?

If you are assigned to the active treatment group and receive FMT, there are some possible side effects that you should be aware of. Faecal transplants have not been widely used in patients with cirrhosis yet, but have been used in lots of patients with an antibiotic associated diarrhoea called 'clostridium difficile.' Small numbers of patients in these studies reported some abdominal distension/bloating, flatulence, diarrhoea or constipation as side effects of the treatment. Part of this study is to assess how well tolerated the treatment is and whether or not patients with cirrhosis experience similar symptoms.

Side effects of blood sampling can include bruising and it can be uncomfortable. However, every effort will be made to perform blood sampling at the same time as regular blood tests to minimise any discomfort. There should be no side effects of sampling your urine and stool as we will simply ask you to collect the samples when you go to the toilet as normal. Saliva will be collected by asking you to rinse your mouth out with water and to spit into a collection pot.

What are the possible disadvantages and risks of taking part?

We hope that the knowledge we would gain from this study will improve our understanding of the way in which FMT works, and the role of the gut and immune system in liver cirrhosis. Patients who received FMT in a small study in America did well and had fewer hospital admissions than those in the 'standard of care' group. FMT appears to be safe in the considerable numbers of patients who have received it for other reasons. There are no disadvantages or risks involved in taking part with regard to the samples being obtained, and the amount of blood taken will not be harmful to you. The placebo treatment is not harmful, but is not expected to have any benefit on your clinical condition as it contains no active drug or therapy. All patients, regardless of whether they receive placebo or FMT have the same rigorous follow up and support from the trials team.

You will be closely monitored after the treatment is given and will be seen at regular intervals afterwards to see if you have experienced any side effects. We do not anticipate any serious problems to occur after the FMT or placebo treatment, but you will be given contact numbers should you run into any problems outside of the trial visits.

Apart from the side effects of FMT, all patients (those on placebo and active treatment) will need to take bowel preparation, which can cause dehydration. The endoscopy itself also has a small risk of bleeding, perforation (this is very rare and is thought to occur in around only 1 in 2000 tests) and damage to teeth and dental work. There is also a risk of aspiration of the FMT or placebo into the lungs, therefore you will be monitored closely after the procedure to monitor for this.

What are the possible benefits of taking part?

You will be taking part in a new and innovative trial to assess the effect of FMT on cirrhosis. There are few treatments available to patients with cirrhosis, aside from liver transplantation. Unfortunately due to the scarcity of donor organs, even those listed for transplant can wait months or even years for a new liver. Others are too unwell or frail to be listed for transplant. We hope that looking at FMT in more detail may provide a much needed treatment for patients with cirrhosis who are not suitable for transplant or to help those awaiting a new liver. This study is designed to look at the safety and tolerability of the treatment to see if a larger study would show an improvement in wellbeing for these patients.

What if I lose the ability to provide consent after enrolling to the study ('loss of capacity)?

In the event that you lose the ability to provide ongoing consent during the research due to your illness, having been able to provide consent at the start of the study, we will approach an appropriate relative or friend on your behalf to ask them if they think that you would have wanted to continue participation in this study. This person is termed a 'Consultee' and they are asked to set aside their own views and provide advice on you continuing to participate in the research, taking into consideration your wishes and interests.

Any advance decisions you may have made, and that they are aware of, should take precedence. If the Consultee decides that you would have no objection to continue taking part they will be asked to read and sign a consultee declaration form. They will then be given a copy to keep. If they decide that you would not wish to continue taking part we will withdraw you from the study; this will not, in any way, affect the standard of care you receive. If the Consultee we approach does not feel able to take on this role they may want to identify someone else to do it. They will be asked to take time to decide whether or not they feel that you would wish to continue taking part. They will be encouraged to ask the research team if there is anything that is not clear or if they would like more information. The information they will be provided with is the same as that provided to you.

You may wish to identify a suitable Consultee at this stage given the above, so that if you do lose the ability to provide consent after you are enrolled (should you decide to participate in the study) we can approach a person of your choice.

What if something goes wrong?

If you have a concern about any aspect of this study, you should ask to speak to your study doctor who will do their best to answer your questions (contact details on page 8) If you remain unhappy and wish to complain formally, you can do this through the NHS Complaints procedure by contacting your local Patient Advice Liaison Service (PALS) office. Details of your local office can be obtained by asking your study doctor, GP, telephoning your local hospital or looking on the NHS choices website. http://www.nhs.uk/pages/home.aspx

Every care will be taken in the course of this study. However in the unlikely event that you are injured by taking part, compensation may be available.

In the event that something does go wrong and you are harmed during the research and this is due to someone's negligence then you may have grounds for a legal action for compensation against King's College Hospital NHS Foundation Trust & King's College London, but you may have to pay your legal costs.

Regardless of this, if you wish to complain, or have any concerns about any aspect of the way you have been approached or treated by members of staff or about any side effects (adverse events) you may have experienced due to your participation in the study the normal National Health Service

complaints mechanisms are available to you. Please ask your study doctor if you would like more information on this.

Will my taking part in this study be kept confidential?

Yes. Once you have consented to take part in the study, you will be given a unique study number, which will be used to identify any samples and information collected during the research.
Some information regarding you and your condition will be recorded as part of this study. We will not record any personal identifiable information (name, date of birth or contact details) as part of the research records. All information will be stored anonymously in a password-protected database, or in a file in a secure research office. Only your treating doctors and the research team will have access to this information.

We will inform your General Practitioner (GP) of your participation in the study - this is routine for this type of research and helps them to care for you during the trial. We advise that you inform your private medical insurance provider (if you hold private medical insurance) of your participation in this study - this again is routine procedure for this type of research.

What will happen to the results of the research study?

When we have results for an adequate number of patients after the study is completed we plan to publish them in an international journal so that the information can benefit as many people as possible. We can provide you with a brief summary of the results of the study when available should you desire this.

What will happen to any samples that I give?

Every effort will be made to ensure blood samples for the study are taken at the same time as samples are taken as part of your routine clinical care – so that there will be no additional discomfort to you as a result of being involved in this study. The amount of blood taken per time point for the study is approximately 50mls or 10 teaspoonfuls. This is a small amount for the body (which contains about 5L of blood, or 5000mL) and will not adversely affect you in any way.

Samples of blood and other fluids obtained may be used immediately or saved for human or bacterial DNA or immune system analysis in the future, in the Liver Biobank. Some of the actual tests on the samples that are obtained as a result of you taking part in the study will be performed on site at King's College Hospital NHS Foundation Trust, whilst some samples will be transferred to other collaborator laboratories in the UK and France. At all times any samples that are transferred and stored will be coded anonymously regardless of which laboratory they are sent to.

Contact details:

Thank you for taking the time to read this information.

If there is any other information you would like, please do not hesitate to contact us on the numbers below. Out of hours or if a response on the above contact number is unavailable, it is possible to contact the on-call Hepatology Registrar – via KCH Switchboard – who will pass any messages on to the senior medical staff involved.

Principal investigator: Dr Debbie Shawcross Tel: 02032992504
Clinical research fellow: Dr C Woodhouse Tel: 02032992504
Research nurse: Ane Zamalloa Tel: 0203299 7623

What will happen to me if I take part (DETAILED EXPLANATION)?

You will be assessed for your eligibility to be involved in this study, and if you are happy to proceed will sign the consent form with a member of the research team. You will then be randomly allocated to one of two treatment groups:

1) the 'active treatment' group: participants in this group will receive the treatment being tested (FMT), or
2) the comparison or 'control group'; participants in this group will receive a 'placebo' treatment, which is a 'dummy' treatment and is indistinguishable from the actual treatment given to participants in the 'active treatment' group.

Before starting treatment with either of the above, you will undergo baseline assessments, which involve the following (those marked with * are being done as part of the study):

- Medical history including current medication usage.
- Physical examination including blood pressure, pulse rate, height, weight.
- Dietary questionnaire* and quality of life questionnaire*
- Blood sampling: will include tests of blood counts, kidney and liver function performed as part of your routine clinical care as well as checks for various viruses that can affect the liver.
- Blood sampling*: to measure endotoxin levels, immune function including DNA analysis and metabolic profiling.
- Urine sampling*: we will ask you to provide a urine sample (approx. 20mls) to measure metabolic profiling. If you are a woman of child-bearing age, we will also do a pregnancy test.
- Stool sampling*: we will ask you to provide stool samples to measure what types of bacteria are found, and in what patterns and quantities.
- Salivary sampling: *: we will ask you to provide saliva samples from your mouth to measure what types of bacteria are found, and in what patterns and quantities.
- If you have fluid in your abdomen called "ascites" we would like to take a sample of the fluid to check for infection. This involves passing a small needle into your abdomen under aseptic conditions. This test is voluntary and you do not have to take part, but it would help us to check for infection in the fluid and would mean we could treat any infection early, should one be present*.

After the baseline assessments have been completed, you will come up to the endoscopy unit to have the FMT or placebo treatment given. The procedure will be explained to you and the risks and benefits discussed. Endoscopy is a safe test, but like all procedures has some risks. The main risks to be aware of are a small risk of bleeding and the risk of perforation (1 in 9000.) There is also a risk of damaging teeth or dental work and a small risk of 'aspiration' which is where fluid from the stomach goes into the lungs. This is why we ask you not to eat for six hours prior to the test, to make sure the stomach is empty. Some people also develop a sore throat after the procedure. This should get better on its own.

You will also be given a medicine prior to the test called 'Moviprep' to help clear your bowels of their usual bacteria prior to the treatment with FMT or placebo. This is a safe medicine, but will cause you to have loose stools. This is essential to its function, but it is important you drink

plenty of fluid to prevent dehydration. Side effects can include dehydration, nausea and vomiting.

You will take one sachet of Moviprep mixed with a litre of water at 19.00 the night before the test. You can then have a light meal. On the morning of the endoscopy you will drink another sachet of Moviprep mixed with one litre of water at around 7.00am. You can take your usual medications as normal. There is a lot of fluid to drink, so you may find it easier if it is chilled or mixed with a squash (not blackcurrant) to make it easier to drink.

You will undergo similar assessments to the baseline visit at 7, 30 and 90 day intervals as you did just before starting treatment. You will in addition undergo the following as part of the study:

- Study treatment adverse events monitoring*: this to check whether you may have experienced any side effects that may be due to the study medication.

Your last visit will be at 90 days from the start of the trial. Samples will be collected and you will be reviewed by the team to check for any side effects. You will have the opportunity to ask questions to the team at all visits throughout the duration of the study and if you have any concerns outside of these visits you will be told who to contact.

Thank you for taking the time to read this information. If there is any other information you would like, please do not hesitate to contact us (020 3299 9000 ext. 32504).

Dr C Woodhouse, Dr V. C. Patel, Professor A Sanchez-Fueyo, Dr D. Shawcross
Institute of Liver Studies, Kings College Hospital
