# Supplementary material for: PROFIT, a PROspective, randomised placebo controlled feasibility trial of Faecal mIcrobiota Transplantation in cirrhosis: study protocol for a single-blinded trial
Source: BMJ Open. 2019 Feb 15;9(2):e023518. doi: 10.1136/bmjopen-2018-023518 (PMC6398649; doi:10.1136/bmjopen-2018-023518)
Supplement: Supplementary data [file bmjopen-2018-023518supp002.pdf]

Faecal Microbiota Transplant (FMT) for recurrent or refractory *Clostridium difficile* Infection (CDI)

Donor screening questionnaire – Part 1

Name:

DOB:

Hospital number:

NHS number:

Contact details (preferably mobile):

Date of assessment:

Name / position of assessor:

Donor type	☐ Named donor<br>☐ Anonymous donor
If Named donor, name and hospital number of recipient	
If Named donor, relationship to recipient	
If Named donor, does the recipient normally live in the same dwelling?	☐ Yes ☐ No
Age	Exclude if <18 or >60
Gender	☐ Male ☐ Female
Ethnicity	☐ White - White British<br>☐ White - White Irish<br>☐ White - Other<br>☐ Mixed race – White and Black Caribbean<br>☐ Mixed race – White and Black African<br>☐ Mixed race – White and Asian<br>☐ Mixed race – Other<br>☐ Asian or Asian British – Indian<br>☐ Asian or Asian British – Bangladeshi<br>☐ Asian or Asian British – Pakistani<br>☐ Asian or Asian British – Other<br>☐ Black or Black British – Caribbean<br>☐ Black or Black British – African<br>☐ Black or Black British – Other<br>☐ Chinese<br>☐ Other
Height cm	

Weight kg	
BMI	Exclude if BMI>25
Has your weight changed by more than 5lb / 2kg in the past 6 months?	☐ Yes ☐ No<br><br>Detail:
Describe your diet (as many as apply):	☐ Omnivore<br>☐ Vegetarian<br>☐ Vegan<br>☐ Kosher<br>☐ Halal<br>☐ Raw food only<br>☐ Pescatarian<br>☐ No red meat<br>☐ Low carbohydrate<br>☐ Lactose free<br>☐ Gluten free<br>☐ Other
How many portions of fruit and vegetables do you consume per day?	☐ one or less<br>☐ two to three<br>☐ three to four<br>☐ five to six<br>☐ seven or more
How many servings of cow, sheep or goats milk do you consume per day?	☐ one or less<br>☐ two to three<br>☐ three to four<br>☐ five to six<br>☐ seven or more
Alcohol – units/week	
Smoking/day	
Normal bowel habit – average Bristol Stool Consistency	☐ 1<br>☐ 2<br>☐ 3<br>☐ 4<br>☐ 5<br>☐ 6<br>☐ 7<br><br>Exclude if 6 or 7
Normal bowel habit – average frequency	☐ >2/day<br>☐ once to twice daily<br>☐ once / 2 days<br>☐ <once / 2 days<br><br>Exclude if active diarrhoea (>3 UBM/day for at least 2 consecutive days)

During the past 7 days how many days were you physically active for a cumulative total of >60 mins/day?	
Have you ever been rejected as a blood donor/told not to donate?<br>If yes, why?	☐ Yes ☐ No<br><br>Exclude if YES
What is your country of birth?	
Have you ever resided in another country (other than UK) for >5 years?<br>If so which countries and when?	☐ Yes ☐ No
Do you currently have a profession that is associated with an increased risk of blood-borne transmissible diseases (e.g. daily contact with patients/inmates)?<br>If yes, what profession?	☐ Yes ☐ No<br><br>Exclude if health/social care worker with direct patient contact
Have you ever had a needle-stick or injury from a blood contaminated object from someone else? If yes, when and how was this follow up?	☐ Yes ☐ No
Have you ever injected yourself or been injected with illegal or non-prescribed drugs including body building drugs or cosmetics (even if this was only once or a long time ago?)	☐ Yes ☐ No<br><br>Exclude if YES
Have you ever had a tattoo?<br>If yes, when and in which country was it performed?	☐ Yes ☐ No<br><br>Exclude if within past 4 months
Have you ever had a piercing?<br>If yes, when and in which country was it performed?	☐ Yes ☐ No<br><br>Exclude if within past 4 months
Have you ever had acupuncture?<br>If yes, when and in which country was it performed?	☐ Yes ☐ No<br><br>Exclude if within past 4 months
Have you ever had an operation or undergone clinical treatment in a hospital with poor hygienic conditions?<br>If yes, when and in which country was it performed?	☐ Yes ☐ No
Have you ever had a rare infectious disease (e.g. tuberculosis, trypanosomiasis)?<br>If yes, when and which disease?	☐ Yes ☐ No
Have you ever been vaccinated against Hepatitis A or B?<br>If yes, which?	☐ Yes ☐ No

Have you received a live vaccine in the past 6 months?	☐ Yes ☐ No
In the last 12 months have you had sex with anyone who is HIV positive?	☐ Yes ☐ No<br>Exclude if Yes
In the last 12 months have you had sex with anyone with hepatitis B, hepatitis C or HTLV?	☐ Yes ☐ No<br>Exclude if Yes
In the last 12 months have you had sex with anyone who has ever been given money or drugs for sex?	☐ Yes ☐ No<br>Exclude if Yes
In the last 12 months have you had sex with anyone who has ever injected drugs?	☐ Yes ☐ No<br>Exclude if Yes
In the last 12 months have you had sex with anyone who may ever have had sex in parts of the world where HIV/AIDS is very common (this includes most countries in Africa)?	☐ Yes ☐ No<br>Exclude if Yes
**Male donors ONLY:**<br>In the last 12 months have you ever had oral or anal sex with a man, with or without a condom?	☐ Yes ☐ No<br>Exclude if Yes
**Female donors ONLY:**<br>In the last 12 months have you had sex with a man who has ever had oral or anal sex with another man, with or without a condom?	☐ Yes ☐ No<br>Exclude if Yes
Have you ever been treated for an intestinal infection?<br>If yes, which one and when?	☐ Yes ☐ No
Do you have any intestinal conditions:<br>Barrett's Oesophagus<br>Coeliac disease<br>Diverticular disease<br>Dyspepsia<br>Gastric ulcer<br>Gastro-oesophageal reflux disease<br>Hepatitis<br>H. pylori infection<br>Crohns disease<br>Ulcerative colitis<br>Other inflammatory bowel diseases<br>Irritable Bowel Syndrome<br>Lactose intolerance<br>Liver disease<br>Pancreatitis<br>Gastrointestinal malignancy or polyps	 ☐ Yes ☐ No<br>☐ Yes ☐ No<br>☐ Yes ☐ No<br>☐ Yes ☐ No<br>☐ Yes ☐ No<br>☐ Yes ☐ No<br>☐ Yes ☐ No<br>☐ Yes ☐ No<br>☐ Yes ☐ No<br>☐ Yes ☐ No<br>☐ Yes ☐ No<br>☐ Yes ☐ No<br>☐ Yes ☐ No<br>☐ Yes ☐ No<br>☐ Yes ☐ No<br>☐ Yes ☐ No<br><br>Exclude if Inflammatory Bowel Disease, Irritable Bowel Syndrome, GI malignancy, Hepatitis A, B, C or E

Have you used any antibiotics in the last 3 months?	☐ Yes ☐ No<br><br>Exclude if Yes
Have you had a fever in the last 2 weeks?	☐ Yes ☐ No<br><br>Exclude if Yes
Have you ever been incarcerated in prison?	☐ Yes ☐ No<br><br>Exclude if in past 4 months
Have you ever been immunosuppressed (e.g. during treatment for cancer, or for a solid organ transplant)?<br>If yes, when and why?	☐ Yes ☐ No
Have you ever had major gastrointestinal surgery?<br>If yes, when and why?	☐ Yes ☐ No<br><br>Relative exclusion criteria
Have you ever suffered from metabolic syndrome?	☐ Yes ☐ No<br><br>Relative exclusion criteria
Have you ever suffered from any autoimmune condition (e.g. rheumatoid), asthma or eczema?<br>If yes, which, and do you take any treatment?	☐ Yes ☐ No<br><br>Relative exclusion criteria
Have you ever had any chronic pain or fatigue syndromes?<br>If yes, which?	☐ Yes ☐ No<br><br>Relative exclusion criteria
Have you any history of CJD or other prion disease in your family? If yes, please specify<br>Patients should be considered to be at risk from genetic forms of CJD if they have or have had<br>1. Genetic testing, which has indicated they are at significant risk of developing CJD or other prion disease<br>2. A blood relative known to have a genetic mutation indicative of genetic CJD or other prion disease<br>3. Two or more blood relatives affected by CJD or other prion disease	☐ Yes ☐ No
Have you ever received growth hormone or gonadotrophic treatment? If yes, please specify;<br>i) Whether the hormone was derived from human pituitary glands<br>ii) The year of the treatment<br>iii) Whether the treatment was received in the UK or in another country	☐ Yes ☐ No

Recipients of hormone derived from human pituitary glands eg growth hormone or gonadtotrophin have been identified as potentially at risk of CJD. In the UK, the use of human growth hormone was stopped in 1985 but human-derived products may have been continued to be used in other countries. In the UK, the use of human-derived gonadotrophin was discontinued in 1973 but may have been continued in other countries after this time.	
Have you ever had surgery on your brain or spinal cord?<br>People who underwent intradural neurosurgical or spinal procedures before August 1992 may have received a graft of human-derived dura mater and should be treated as at increased risk, unless evidence can be provided that human-derived dura mater was not used. Patients who received a graft of human-derived dura mater between 1980 and August 1992 are at increased risk of both sporadic CJD and vCJD.	☐ Yes ☐ No
Are you normally resident in the UK?<br>If No state country of usual residence	☐ Yes ☐ No
Which countries have you visited in the last 12 months and what was the duration of stay?	
In the past 12 months have you been admitted to a hospital in a country other than the UK?<br><br>If yes state when and which countries	☐ Yes ☐ No
In the past 12 months have you been admitted to another hospital in London or Manchester?<br><br>If yes state when and which hospitals	☐ Yes ☐ No
List all current medications:	

	Exclude if any regular prescribed drugs (except OCP)

Is patient eligible to donate?	☐ Yes ☐ No
If No document reasons:	
If Yes proceed to screening tests<br>Ensure consent for testing documented	
Sign and date (health care practitioner) to indicate patient has provided consent	

Obtain serology for:	Requested?
HIV antigen/antibody	☐ Yes
HTLV-1/HTLV-II antibodies	☐ Yes
Hepatitis A IgG (add on IgM if positive)	☐ Yes
Hepatitis B surface antigen	☐ Yes
Hepatitis C IgG	☐ Yes
Syphilis serology (T. pallidum antibodies)	☐ Yes
CMV IgG (add on IgM if positive)	☐ Yes
EBV Serology	☐ Yes
Strongyloides serology	☐ Yes

Provide stool sample collection pots and request forms x3 for patient to take home. Instruct patient to provide three separate samples over 2-3 days, ensuring the pot is filled halfway in order to complete all testing.

Stool testing for:	Requested?
Bacterial culture (Campylobacter, Salmonella, Shigella, E. coli O:157) x3	☐ Yes
Ova, Cysts and Parasites x3	☐ Yes
C. difficile	☐ Yes
Resistant Gram negative organism screen	☐ Yes
MRSA screen	☐ Yes
Helicobacter pylori stool antigen	☐ Yes
E. histolytica PCR (not orderable on PCR)	

Arrange follow up visit for a minimum of 2-3 weeks time

Follow-up visit

Serology results:	Result
HIV antigen/antibody	☐ Detected ☐ Not detected

HTLV-1/HTLV-II antibodies	☐ Detected ☐ Not detected
Hepatitis A IgG (add on IgM if positive)	☐ Detected ☐ Not detected
Hepatitis B surface antigen	☐ Detected ☐ Not detected
Hepatitis C IgG	☐ Detected ☐ Not detected
Syphilis serology (T. pallidum antibodies)	☐ Detected ☐ Not detected
CMV IgG (add on IgM if positive)	☐ Detected ☐ Not detected
EBV Serology	☐ Detected ☐ Not detected
Strongyloides serology	☐ Detected ☐ Not detected

Stool results:	Result
Bacterial culture (Campylobacter, Salmonella, Shigella, E. coli O:157) x3	☐ Detected ☐ Not detected
Ova, Cysts and Parasites x3	☐ Detected ☐ Not detected
C. difficile	☐ Detected ☐ Not detected
Resistant Gram negative organism screen	☐ Detected ☐ Not detected
MRSA screen	☐ Detected ☐ Not detected
Helicobacter pylori stool antigen	☐ Detected ☐ Not detected
E. histolytica PCR (not orderable on PCR)	☐ Detected ☐ Not detected

Is all testing complete?	☐ Yes ☐ No
Accept as donor?	☐ Yes ☐ No
Any referrals required	☐ Yes ☐ No
Details of referrals made:	

Provide donor with stool collection kit x2 (contains collection instructions and copy of patient screening questionnaire 2). Explain requirement for stool to be fully processed within 6 hours of production.

The donor may provide multiple donations, but screening questionnaire 2 **MUST** be completed with **EACH** donation

If yes to any question, must undergo repeat full screening (however samples may be banked and quarantined for release after repeat testing).

If No to all questions samples may be released immediately and donor may continue to provide unlimited donations for 3 months after which time repeated screening will be required.
