# Supplementary material for: PROFIT, a PROspective, randomised placebo controlled feasibility trial of Faecal mIcrobiota Transplantation in cirrhosis: study protocol for a single-blinded trial
Source: BMJ Open. 2019 Feb 15;9(2):e023518. doi: 10.1136/bmjopen-2018-023518 (PMC6398649; doi:10.1136/bmjopen-2018-023518)
Supplement: Author's manuscript [file bmjopen-2018-023518.draft_revisions.pdf]

BMJ Open is committed to open peer review. As part of this commitment we make the peer review history of every article we publish publicly available.

When an article is published we post the peer reviewers' comments and the authors' responses online. We also post the versions of the paper that were used during peer review. These are the versions that the peer review comments apply to.

The versions of the paper that follow are the versions that were submitted during the peer review process. They are not the versions of record or the final published versions. They should not be cited or distributed as the published version of this manuscript.

BMJ Open is an open access journal and the full, final, typeset and author-corrected version of record of the manuscript is available on our site with no access controls, subscription charges or pay-per-view fees (http://bmjopen.bmj.com).

If you have any questions on BMJ Open's open peer review process please email info.bmjopen@bmj.com

BMJ Open

PROFIT: A PROspective, randomised placebo controlled feasibility trial of Faecal mIcrobiota Transplantation in cirrhosis

Journal:	*BMJ Open*
Manuscript ID	bmjopen-2018-023518
Article Type:	Protocol
Date Submitted by the Author:	05-May-2018
Complete List of Authors:	Woodhouse, Charlotte; King's College London, Hepatology<br>Patel, Vishal; Kings College London, Hepatology<br>Goldenberg, Simon; Guy's & St. Thomas' NHS Foundation Trust and King's College,<br>Sanchez Fueyo, Alberto; Kings College London, Hepatology<br>China, Louise; UCL, Division of Medicine<br>O'Brien, Alastair; UCL, Division of Medicine<br>Flach, Clare; 1. School of Population Health & Environmental Sciences, Faculty of Life Sciences & Medicine, King's College London<br>Douiri, Abdel; 1. School of Population Health & Environmental Sciences, Faculty of Life Sciences & Medicine, King's College London<br>Shawcross, Debbie ; Kings College London, Liver Sciences, 1st Floor JBC, School of Immunology & Microbial Sciences Faculty of Life Sciences & Medicine, King’s College London, Denmark Hill Campus, London, SE5 9RS
Keywords:	cirrhosis, faecal microbiota transplantation, gut microbiota, feasibility

SCHOLARONE™
Manuscripts

PROFIT: A PROspective, randomised placebo controlled feasibility trial of Faecal mIcrobiota Transplantation in cirrhosis

C. Woodhouse[1], V. Patel[1], S. Goldenberg[2], A. Sanchez-Fueyo[1], L. China[3], A. O'Brien[3], C. Flach[4], A. Douiri[4], D. Shawcross[1]

1. **Liver Sciences, 1st Floor JBC, School of Immunology & Microbial Sciences Faculty of Life Sciences & Medicine, King's College London, Denmark Hill Campus, London, SE5 9RS**
2. **Directorate of Infection, Guy's and St Thomas' NHS Foundation Trust, London**
3. **Division of Medicine, University College London (UCL), London**
4. **School of Population Health & Environmental Sciences, Faculty of Life Sciences & Medicine, King's College London**

Corresponding author: Dr Charlotte Woodhouse, Liver Sciences, 1st Floor JBC, School of Immunology & Microbial Sciences, Faculty of Life Sciences & Medicine, King's College London, Denmark Hill Campus SE5 9RS charlottewoodhouse@nhs.net Tel 02032993713 Fax: 020232993899

Abstract

Introduction

Patients with advanced cirrhosis have enteric bacterial dysbiosis and translocation of bacteria and their products across the gut epithelial barrier. This culminates in systemic inflammation and endotoxemia, inducing innate immune dysfunction which predisposes to infection, and development of complications such as bleeding, sepsis and hepatic encephalopathy. This feasibility study aims to assess the safety of administering faecal microbiota transplant to patients with cirrhosis and explore the effect of the intervention on their prognosis by achieving restoration of a healthy gut microbiome.

Methods and Analysis

PROFIT is a single centre, randomized, single-blinded, placebo-controlled study evaluating Faecal Microbiota Transplantation (FMT) against placebo. Patients with advanced but stable cirrhosis with a MELD score between 10 and 16 will be recruited. Twenty-four patients will be randomized to FMT plus standard of care (as per our institutional practice) and eight patients to placebo in a ratio of 3:1. The patients will be assessed in the Clinical Research Facility at King's College Hospital and the FMT/placebo will be administered in the endoscopy department within 5 days of recruitment and within 24 hours of randomisation. Patients will be evaluated at baseline before the study intervention is administered and at 7, 30 and 90 days post intervention.

For peer review only

Ethics and dissemination

Research Ethics approval was given by the London South East Research Ethics committee (ref 17/LO/2081)

Results

Will be disseminated via peer reviewed journals and international conferences. The recruitment of the first patient will occur on or shortly after 16/04/2018.

Trial Registration number

The study has been registered with *ClinicalTrials.gov* as part of the ethics application and approval process – reference number: NCT02862249. The trial is registered with the European Medicines Agency (EudraCT 2017-003629-13) and has been adopted by the NIHR (IRAS 197237). This manuscript refers to the version 2.0 of the protocol; pre-results.

Strengths and limitations of this study

- This study will demonstrate the feasibility and safety of administering FMT to these patients, however it is not statistically powered to assess for clinically relevant outcomes.
- This is the first study examining the effect of FMT delivered directly into the small bowel in patients with advanced cirrhosis. This RCT does not involve antibiotic pre-treatment in the FMT group, as has been undertaken in the USA in patients with HE.
- This study will provide preliminary data for the development of a further clinical trial to assess the clinical benefit of FMT in advanced cirrhosis.
- One further limitation of the study is its single-blinded design, which was necessary as the FMT and placebo (saline with glycerol) are not matched.

Introduction

Patients with advanced cirrhosis have enteric dysbiosis with small bowel bacterial overgrowth and translocation of bacteria and their products across the gut epithelial barrier (1). This culminates in systemic inflammation and endotoxaemia, inducing innate immune dysfunction which predisposes to infection (2), and development of complications such as bleeding, sepsis and hepatic encephalopathy (3). It also plays a key role in the natural history of cirrhosis by influencing the rate of progression to advanced liver disease and terminal liver failure (4).

Utilising quantitative metagenomics our group has found 75,245 genes differentially expressed between patients with cirrhosis and healthy individuals. Over 50% of these bacterial species are of buccal origin suggesting an invasion of the gut from the mouth in cirrhosis (5). Patients with cirrhosis also have salivary dysbiosis associated with impaired salivary defenses and systemic inflammation. Salivary dysbiosis has been shown to be greater in patients with cirrhosis who developed complications necessitating hospitalisation within 90 days. Modulating the gut microbiota in patients with cirrhosis with the non-

For peer review only

absorbable antibiotic rifaximin has been associated with improved cognitive performance and reduction in endotoxaemia in patients with cirrhosis (6, 7). Moreover, we have recently performed a multi-centre retrospective study including 170 patients in which rifaximin-α therapy given for 90 days significantly (i) reduced hospital re-admission rates after 3 months treatment, impacting significantly on the NHS resource burden and (ii) reduced overall liver disease severity (as measured by the Child Pugh and Model for End Stage Liver Disease scores) raising the possibility that modulation of gut microbiota may significantly modify the natural history of chronic liver failure (8).

These data constitute in our view “proof of principle” that modifying the gut microbiota in patients with cirrhosis improves clinical outcomes. Rifaximin-α was approved by NICE for the prevention of the recurrence of overt hepatic encephalopathy in cirrhosis (9) but considerable concern remains regarding whether long term antibiotic prescription will result in a change in bacterial function and virulence rather than a simple reduction in bacterial population and whether this may drive bacterial resistance to antibiotics in an already functionally immunocompromised population. The question was therefore raised as to whether directly, as opposed to indirectly modulating the gut microbiota utilising faeces from healthy donors may be a safer and more durable therapy. Faecal microbiota transplantation (FMT) is a well-established treatment to stably modify the gut microbiome and has been shown to be safe and efficacious in several disease states resulting from gut dysbiosis including *Clostridium difficile* infection (10) and inflammatory bowel disease (11) .

We hypothesise that in patients with advanced cirrhosis, FMT may reduce the progression to chronic liver failure including the development of jaundice, ascites, bleeding, encephalopathy, infection and organ dysfunction. Whether FMT is feasible in the setting of cirrhosis remains to be investigated. We propose conducting a feasibility trial to determine whether FMT from a healthy donor will alleviate gut dysbiosis and immune dysfunction in advanced cirrhosis.

Methods and Analysis

Primary Objectives:

The primary objective of this study will be to assess whether stabilising gut dysbiosis with FMT in patients with advanced cirrhosis is both feasible and safe.

Primary Endpoints:

The primary endpoints of the study will be twofold. To assess the feasibility of FMT as determined by the recruitment rates (including acceptability of the intervention) and tolerability of FMT e.g. gastro-oesophageal reflux rates. The second primary outcome measure will be to assess the safety of FMT administration, including the incidence of any transmissible bacterial or viral infection that is deemed to have been acquired from the donor including *Clostridium difficile* infection.

For peer review only

Secondary Objectives:

The secondary objectives of the study are to provide preliminary evidence of efficacy for a larger randomised trial, with the purpose of choosing the optimal primary outcome, and estimating the parameters for sample size calculation. We will also collect blood, stool and urine samples from participants to assess the stability of the transplanted gut microbiome by comparing the percentage composition of the stool microbiota on day 7, 30 and 90 with the donor microbiome. Plasma endotoxin (and endotoxin binding protein), pro- and anti-inflammatory cytokine levels, bacterial DNA quantification and serum procalcitonin will be performed at 7, 30 and 90 days as will changes in faecal biomarkers (calprotectin, lactoferrin and M2-Pyruvate Kinase).

Trial Design

PROFIT is a single-centre study. Thirty-two patients will be recruited from out-patient clinics at King's College Hospital (KCH) or from suitable inpatients on the wards. The patients will be recruited as per the inclusion and exclusion criteria listed in table 1. Patients will be randomized in a single blinded fashion in a ratio of 3:1 FMT to placebo. Patients will be unaware of the intervention given, but investigators will not be blinded to the treatment intervention.

Table 1- Inclusion and exclusion criteria

Inclusion Criteria

- 18–75 years
- Confirmed advanced cirrhosis of any aetiology with a MELD (12) score between 10 and 16. The diagnosis of cirrhosis will be based on clinical, radiological, or histological criteria.
- Patients with alcohol-related liver disease must have been abstinent from alcohol for a minimum of 6 weeks.
- Patients must be deemed to have capacity to consent to the study.

For peer review only

Exclusion Criteria

- Severe or life-threatening food allergy
- Pregnancy or breastfeeding
- Patients treated for active variceal bleeding, infection, bacterial peritonitis, overt hepatic encephalopathy or acute-on-chronic liver failure within the past 14 days.
- Patients who have received antibiotics in the past 14 days.
- Active alcohol consumption of >20 grams/day.
- Has had a previous liver transplant
- Hepatocellular carcinoma outside of the Milan Criteria (13)
- Inflammatory bowel disease
- Coeliac disease
- A history of prior gastrointestinal resection such as gastric bypass
- Patient is not expected to survive the duration of the study (90 days).
- Severe renal impairment (creatinine >150 µmol/L)
- HIV positive
- Immunosuppression e.g. more than two weeks treatment with corticosteroids within 8 weeks of intervention, active treatment with tacrolimus, mycophenylate, azathioprine.

Patient Population

The study will include all patients aged 18 to 75 years with capacity to consent and a diagnosis of cirrhosis. Patients will have a MELD (12) score of between 10 and 16. The diagnosis of cirrhosis may be based on radiological, clinical or histological parameters. In the case of alcohol related cirrhosis, patients must have been abstinent for a minimum of six weeks. The exclusion criteria are outlined in table 1.

Consent

All participants will be asked to provide informed consent after having had the opportunity to discuss the trial with the clinician, their family and friends and having read the patient information sheet (PIS- Appendix 1.) Patients who lack capacity will not be enrolled in this study. If patients lose capacity during the trial follow up period, an appropriate legal representative will be consulted and will provide consent to ongoing trial participation after appropriate discussion with the trials team and having read the PIS. If the legal representative does not agree to ongoing trial participation, the subject will be withdrawn from trial follow up.

Figure 1 Study Flow Chart and Anticipated Recruitment (adapted from the PROFIT protocol)

For peer review only

Study Intervention

FMT is prepared in laboratory at St Thomas' Hospital in accordance with the principles of Good Manufacturing Practice (GMP) and under Manufacturing Authorisation for an Investigational Medicinal Product (MIA(IMP)) from the Medicines and Healthcare products Regulatory Agency (MHRA). FMT donors are rigorously screened for blood borne and enteric pathogens prior to donation and undergo questionnaire screening for risk factors at baseline and again at donation to reduce the risk of transmissible infection as outlined in table 2. Stool is processed (diluted and filtered with the addition of glycerol) within 6 hours of donation and stored at -80 degrees Celsius for up to six months (as previously described) (15). Material for FMT has full traceability from donor to recipient and aliquots of donor stool are kept for 30 years to allow further testing in the case of any adverse events. Placebo is a solution of saline with glycerol without faecal matter.

Blood (serology)

- HIV 1+2 serology
- HTLV I/II Ab
- Hepatitis A IgG (and if positive IgM)
- Hepatitis B surface antigen (HBsAg) and core antibody
- Hepatitis C (HCV Ab)
- Hepatitis E
- Syphilis
- CMV/EBV IgG/M
- Strongyloides stercoralis (ELISA)

Stool

- PCR for gastroenteritis agents (*Campylobacter, Salmonella, Shigella* and *E. coli* O:157)
- Ova, cysts and parasites by concentration and microscopy x3
- *C. difficile* test
- Norovirus PCR
- Screen for gentamicin and carbapenem resistant Gram negative organisms
- Screen for MRSA
- Helicobacter pylori antigen
- Entamoeba histolytica PCR

Table 2 blood and stool testing of donor FMT samples

For peer review only

Following identification, all patients will attend a screening visit and sign the informed consent form. If deemed to be eligible they will undergo baseline screening with full clinical history and examination. Medication history will be recorded. Patients will complete a dietary questionnaire. Baseline samples of blood, urine, saliva and stool will be obtained. All patients will subsequently attend for a gastroscopy at which the FMT or placebo will be administered. This will be performed as per the local Endoscopy Unit Protocol using topical local anaesthetic spray or midazolam sedation as per patient preference. Patients will first be asked to take bowel preparation to purge the bowel of its native bacteria. This consists of two sachets of Moviprep® to be taken two hours apart on the evening prior to gastroscopy. Patients will be monitored for side effects in the Clinical Research Facility after the procedure.

Evaluations during and after treatment

Participants will be reassessed at 7 (+/- 5), 30 (+/-7) and 90 (+/-7) days post intervention. They will be reviewed in the Clinical Research Facility and undergo physical examination, review of medications and dietary changes and adverse event monitoring. Samples of blood, urine, saliva and stool will be taken at these visits also. At the end of the 90 day follow up period, patients will return to their usual care pathway.

Statistical analysis

Sample Size

This feasibility study will evaluate feasibility parameters using 95% Confidence Intervals. The sample size has been proposed mainly to enable the trial to be conducted within the allocated budget and with acceptable precision for continuous outcomes. According to the simulation work by Teare MD et al. (18), even with the relatively small pilot sample size of 20, the planned studies would have at least 80% power to detect the target effect size (for continuous outcomes) more than 75% of the time. Teare et al. recommend that 60 to 100 subjects are sufficient to estimate an event rate (such as recruitment rates) with acceptable precision in a feasibility study, while sample sizes between 24 and 50 have been recommended for the accurate estimation of standard deviations. Therefore, we have chosen a sample size of 32 patients in this trial to have reliable data on all critical parameters (including event rates) which can also be utilised when planning a larger intervention trial. For event rates (e.g. recruitment rates) and particularly in the extreme case with lower rates e.g. 10%, we estimate a drop of precision by only 5% using our updated sample size and the minimum recommended by Teare et al. (0.16 for 60 patients vs 0.21 for 32 patients). Figure 2 below illustrates the reduction in precision of different rates

For peer review only

when the sample size increases for binary outcomes. This sample size will also be feasible within the budget and will provide acceptable information for planning a future large clinical trial.

Figure 2 Confidence interval width around one proportion (P) by sample size (N) from PASS software REF - PASS 15 Power Analysis and Sample Size Software (2017). NCSS, LLC. Kaysville, Utah, USA, ncss.com/software/pass.

The sample size of 32 patients will undergo randomisation in a 3:1 ratio. This will allow the study to demonstrate feasibility of randomising, yet providing robust evidence with respect to the feasibility of the treatment, and preliminary evidence of efficacy parameters.

The following feasibility criteria have been established:

- 25% of screened patients will consent: 256 patients will allow the estimate of the 2-Sided 95% confidence interval of the proportion of consented patients, where the distance from the observed proportion to the limit is 0.058 units.
- 50% of patients screened will fulfil inclusion/exclusion criteria for the trial: screening 64 patients will allow the estimate of the 2-Sided 95% confidence interval of the proportion of patients eligible, where the distance from the observed proportion to the limit is 0.128 units.
- 80% of randomised patients will complete treatment and follow up: 26 of 32 patients completing will allow the estimate of the 2-Sided 95% confidence interval for a single proportion, where the distance from the observed proportion to the limit is 0.177 units.

Differences between the placebo and treatment group in efficacy binary outcomes, will be estimated along with the two-sided 95% CI. Assuming the proportion with the outcome in the control group is 0.5 our sample size will allow estimation of the half-width of the confidence intervals for different size of differences as indicated below. Differences of 0.4 or higher will be distinguishable from 0.

Table 3 Half width of confidence intervals for differences in proportions between two groups with given sample size, placebo group p=0.5 (created with PASS software REF - PASS 15 Power Analysis and Sample Size Software (2017). NCSS, LLC. Kaysville, Utah, USA, ncss.com/software/pass.)

Difference	0.1	0.2	0.3	0.4	0.5
Width	0.398	0.392	0.382	0.337	0.347

The half-width of the 95% CI of the difference in means for continuous outcome will be 0.889 SD.

For peer review only

Clinical Endpoints

Clinical and safety events will be listed and summarised by intervention group. MELD (12) scores will be calculated by visit and treatment group.

Data synthesis, analysis and presentation

Feasibility and efficacy outcomes will be summarised using the appropriate descriptive statistics, and 95% Confidence Intervals will be calculated to allow for success and go/no go decisions. Biomarker data will be pre-processed according to the established standards for each platform, and statistical analyses will be performed using non-parametric and permutation based methods which are more appropriate for small sample sizes.

Statistical Software

Analyses will be performed using R and/or Stata statistical software packages.

Discussion

The PROFIT study is the first study to look at the use of FMT delivered directly into the small bowel in patients with cirrhosis. Liver transplantation is a highly selective treatment and only those most likely to benefit will be listed. Depending on blood type patients can wait up to 24 months for a liver transplant. There are even fewer options for patients who are unsuitable for transplantation.

Cirrhosis is often complicated by recurrent admissions with sepsis, variceal bleeding, ascites and hepatic encephalopathy. We know that patients with cirrhosis have enteric dysbiosis and altered gut permeability. The non-absorbable antibiotic rifaximin has proved useful in patients with cirrhosis and chronic hepatic encephalopathy, but the long-term benefits of antibiotic use are unknown and the possibility of development of bacterial resistance remains with all antibiotics. The possibility of re-populating the gut of cirrhotic patients with healthy gut microbiota could be a potential alternative, providing a new treatment approach for these vulnerable individuals.

A group in the USA have recently published the results of the impact of FMT delivered via enema in 10 cirrhotic patients, compared with 10 patients receiving standard of care treatment (16). FMT was found to be safe and the patients in the treatment group did not have any admissions for HE, whereas there were six episodes of HE in the standard of care group. Four of these episodes required hospital admission. The FMT group were treated with broad spectrum antibiotics prior to FMT administration, whereas the standard of care group were not, meaning that the effects of FMT cannot be clearly separated from the antibiotic administration. We hope that PROFIT will add to the knowledge base of the use of FMT in cirrhosis and will provide an accurate assessment of the safety of FMT. We plan to treat the FMT and control groups identically, aside from the administration of FMT to accurately assess its impact, without the confounding factor of antibiotic treatment.

A limitation of our study is the single blinded design. This was selected due to the inherent difficulties in preparing a matched placebo, without introducing substances that may upset

For peer review only

the delicate gut microbiota. The IMP will be delivered by the trial investigators, so it has not been possible to blind the clinicians in this study. Patients and the trial Statistician will be blinded.

The study is not powered to detect differences in clinical outcomes, but may provide evidence for markers relating to clinical outcomes that could be studied in a larger RCT.

Sponsor

The PROFIT trial is co-sponsored by King's College Hospital and Kings' College London. The funding was supplied by an NIHR research grant for patient benefit (PB-PG-0215-36070).

Trial Monitoring Groups

Trial Steering Committee (TSC)

This group will oversee the running of the trial and discuss any issues that may arise throughout the process of recruitment and follow up of patients. The group will be chaired by an independent clinician. Investigators will report to the group on a regular basis. The Data Monitoring Committee (DMC) will inform the TSC if there are any issues raised from its discussions.

Data Monitoring Committee

This is an entirely independent group that analyzes interim data, to determine whether or not the trial is safe to continue. It monitors adverse events and adverse reactions and reacts to any issues and directs the TSC as to whether or not the trial should continue. The DMC undertakes interim statistical analysis using an independent statistician to ensure the ongoing safety and integrity of the trial. The members of this committee are independent of the trial, but will be experienced clinicians with expertise in clinical trials.

Ethics and Dissemination

The ethical permission has been given by the South-East Research Ethics Committee, REC reference 17/LO/2081, IRAS project ID 197237. EUDRACT number 2017-003629-13.
The results of the trial will be analyzed and published in a peer reviewed journal and disseminated at international conferences.

Patient and Public Involvement

A lay reviewer/patient representative at the NIHR reviewed the protocol. This was funded by the Research for Patient Benefit programme. Feedback was taken on board and revisions were subsequently made.

For peer review only

The British Liver Trust, British Society of Gastroenterology Liver Research Group and King's Liver Outpatient Advisory Group (patient group) were all involved in the study set-up and protocol development at all stages.

Results will be disseminated to study participants via the Liver Research Nurse if they indicate an interest in the study outcome.

Funding statement

This work was supported by the NIHR, grant number PB-PG-0215-36070.

Competing Interests Statement

Dr Shawcross has received fees from Norgine and Falk, outside the submitted work. Dr Patel has received a travel bursary from Norgine outside the submitted work. Dr. Goldenberg reports grants and personal fees from Astellas, personal fees from MSD, personal fees from Pfizer, personal fees from Shionogi, outside the submitted work.

Acknowledgements

We would like to thank the clinicians involved, King's College Hospital R&D Department, the NIHR, patient advisers and lay reviewers for their contributions in the set-up of the PROFIT trial. We would also like to acknowledge Liz Allen, our QP, for her contribution to the MHRA approval process.

Author Contributions

CW and DS wrote the manuscript, with assistance from SG, VP and ASF. AD and CF prepared the statistical analysis plan. LC and AO'B advised on study analyses and the format of the protocol for submission. SG provided the expert advice on FMT and has set up the FMT service at GSTT, which will supply the FMT to the study. All authors reviewed and edited the manuscript prior to submission.

Appendix 1- PIS

For peer review only

1. Wiest R, Garcia-Tsao G. Bacterial translocation (BT) in cirrhosis. Hepatology. 2005;41(3):422-33.
2. Jalan R, Fernandez J, Wiest R, Schnabl B, Moreau R, Angeli P, et al. Bacterial infections in cirrhosis: a position statement based on the EASL Special Conference 2013. J Hepatol. 2014;60(6):1310-24.
3. Bajaj JS, Heuman D. M., Hylemin P.B. Sanyal A. J., White M. B. <Altered profile of human gut microbiome is associated with cirrhosis and its complications>. Journal of Hepatology. 2014;60:940-7
.
4. Nolan JP. The role of intestinal endotoxin in liver injury: a long and evolving history. Hepatology. 2010;52(5):1829-35.
5. Qin N, Yang F, Li A, Prifti E, Chen Y, Shao L, et al. Alterations of the human gut microbiome in liver cirrhosis. Nature. 2014;513(7516):59-64.
6. Bajaj JS, Heuman DM, Sanyal AJ, Hylemon PB, Sterling RK, Stravitz RT, et al. Modulation of the metabiome by rifaximin in patients with cirrhosis and minimal hepatic encephalopathy. PLoS One. 2013;8(4):e60042.
7. Shawcross DL. Is it time to target gut dysbiosis and immune dysfunction in the therapy of hepatic encephalopathy? Expert Rev Gastroenterol Hepatol. 2015;9(5):539-42.
8. Patel V OJ, Sturgeon J, Habtemariam Z, Preedy H, Richardson P, et al. Rifaximin Is Efficacious In The Treatment Of Chronic Overt Hepatic Encephalopathy: A UK Liver Multi-Centre Experience [Abstract]Suppl 1 Gut 2014;63.
9. NICE. National Institute for Health and Care Excellence. Rifaximin for the maintenance treatment of hepatic encephalopathy. 2015 Mar. Report No.: ID496.
.
10. NICE. National Institute for Health and Care Excellence. Faecal microbiota transplant for recurrent Clostridium difficile infection. 2014 Mar. Report No.: Interventional Procedure Guidance 485.
11. Suskind DL, Brittnacher MJ, Wahbeh G, Shaffer ML, Hayden HS, Qin X, et al. Fecal microbial transplant effect on clinical outcomes and fecal microbiome in active Crohn's disease. Inflamm Bowel Dis. 2015;21(3):556-63.
12. Kamath PS, Wiesner RH, Malinchoc M, Kremers W, Therneau TM, Kosberg CL, et al. A model to predict survival in patients with end-stage liver disease. Hepatology. 2001;33(2):464-70.
13. Mazzaferro V RE, Doci R, Andreloa S, Pulvirenti A, Bozzetti F, Montalto F, Ammatuna M, Morabito A, Gennari L Liver transplantation for the treatment of small hepatocellular carcinomas in patients with cirrhosis. New England Journal of Medicine. 1996;334(11):693-700.
14. PROFIT A Prospective, radnsomised placebo controlled feasibility trial of faecal microbiota transplantation in cirrhosis Version 2.0. N/A. 2018.
15. Goldenberg SD BR, Beales I, Digby-Bell J, Irving P, Kellingray L, Narbad A, Franslem-Elumogo N. Comparison of different strategies for providing fecal microbiota transplantation to treat patients with recurrent Clostridium difficile infection in two English hospitals: a review. Infectious Diseases and Therapy 2018.

For peer review only

16. Bajaj JS, Kassam Z, Fagan A, Gavis EA, Liu E, Cox IJ, et al. Fecal Microbiota Transplant from a Rational Stool Donor Improves Hepatic Encephalopathy: A Randomized Clinical Trial. Hepatology. 2017.

For peer review only

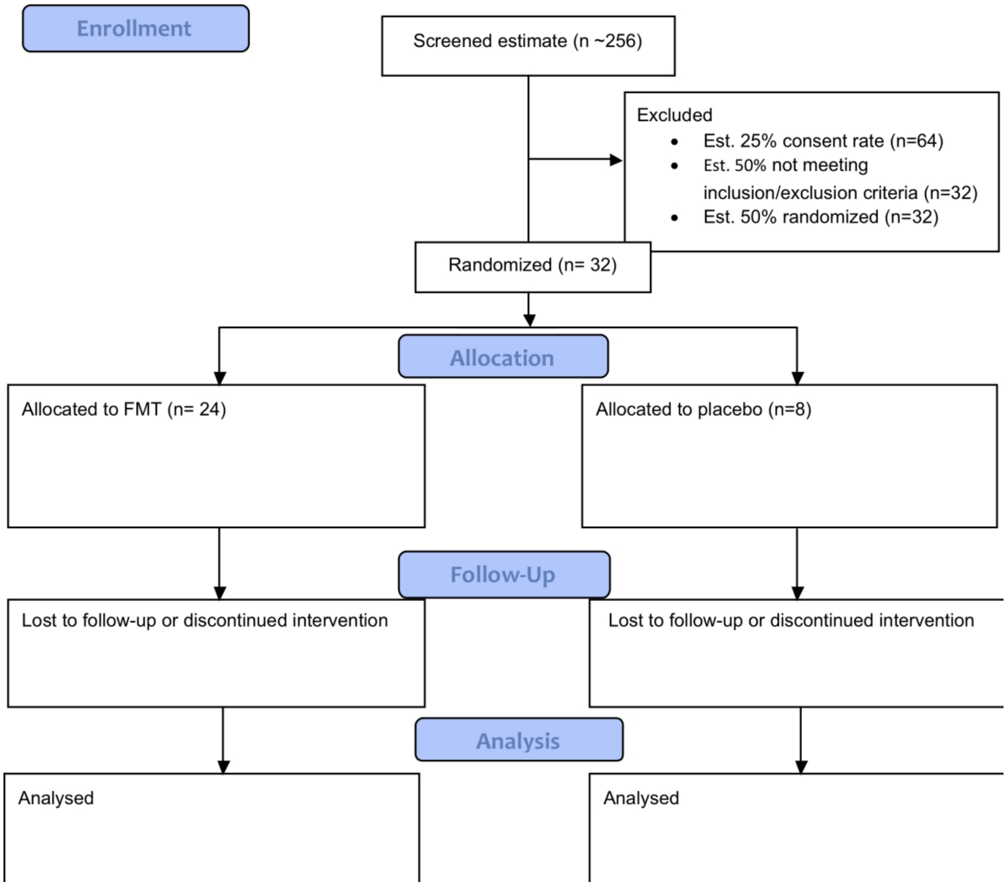

Figure 1 Study Flow Chart and Anticipated Recruitment

190x212mm (300 x 300 DPI)

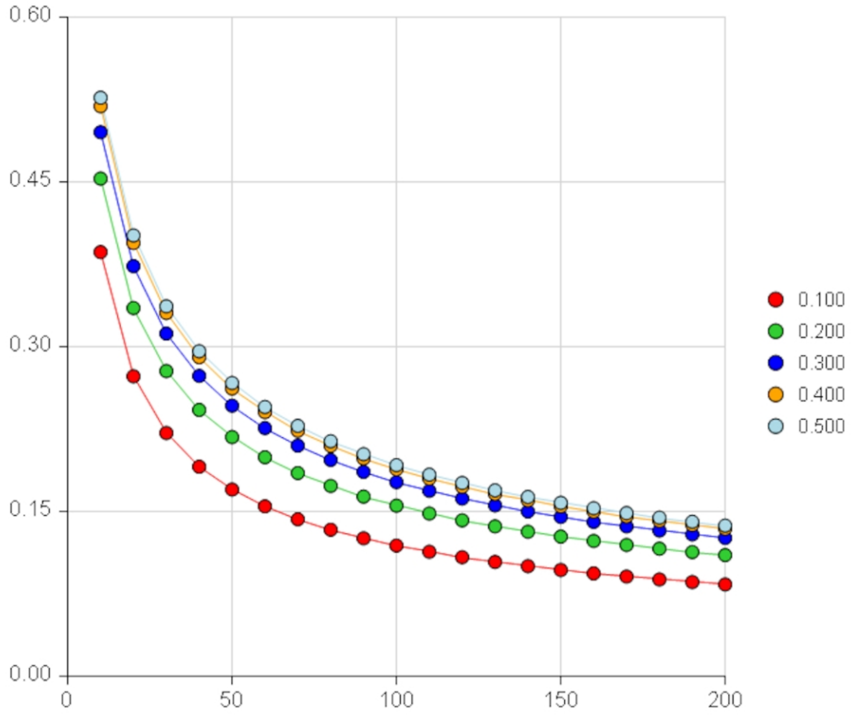

Figure 2 Confidence interval width around one proportion (P) by sample size (N) from PASS software

101x90mm (300 x 300 DPI)

King's College Hospital NHS
NHS Foundation Trust
Institute of Liver Studies

Participant Consent Form (PROFIT)

Title of Study: **A Prospective, randomized placebo controlled feasibility trial of faecal microbiota transplantation in cirrhosis.**

Researchers: **Dr C Woodhouse, Dr V. C. Patel, Prof A. Sanchez-Fueyo, Dr D. Shawcross.**

Participant Identifier N^{o}: ☐ *Initials:* ☐

Please print your initials in the boxes:

1. I confirm that I have read and understand the participant information sheet ________________for the above study and have had the opportunity to ask questions. ☐

2. I understand that my participation in this study is voluntary and that I am free to withdraw at any time, without giving any reason, without my medical care or legal rights being affected. ☐

3. I understand that sections of my medical records may be looked at by responsible individuals or regulatory authorities where it is relevant to taking part in the research. I give permission for these individuals to access my records. ☐

4. I consent to the following samples being collected, stored and analysed: Blood, stool, urine, saliva and ascites (if present) ☐

5. I agree/do not agree (please delete as appropriate) that any surplus samples (including DNA) will be transferred into and stored in the Liver Biobank, and may be used in future, anonymised research Yes/No

6. I understand that my General Practitioner will be informed of my participation in this study. ☐

7. I agree to take part in the above study ☐

8. I would like to be sent information regarding the results of this study when available. ☐

For peer review only - http://bmjopen.bmj.com/site/about/guidelines.xhtml

King's College Hospital NHS
NHS Foundation Trust
Institute of Liver Studies

**NAME OF PARTICIPANT**	**Signature**	**Date**
**NAME OF PERSON OBTAINING CONSENT**	**Signature**	**Date**
**WITNESS (IF APPLICABLE)**		

Records:

1) Participant *(copy)*
2) Hospital notes *(copy)*
3) Researcher *(original)*

For peer review only - http://bmjopen.bmj.com/site/about/guidelines.xhtml

King's College Hospital NHS
NHS Foundation Trust

Participant Information Sheet

Short Title: **PROFIT**

Full Title of Study: **A Prospective, randomized placebo controlled feasibility trial of faecal microbiota transplantation in cirrhosis**

Chief Investigator: **Dr D. Shawcross**

Researchers: **Dr C Woodhouse, Dr V. C. Patel, Prof A. Sanchez-Fueyo,**

For peer review only

Introduction

You are invited to take part in this research study. Before you decide it is important for you to understand why the research is being done and what it will involve. Please take time to read the following carefully and discuss it with friends, family or your GP if you wish. Ask us if there is anything that is not clear or if you would like more information. Take time to decide whether or not you wish to take part.

What is the purpose of the study?

Our bodies contain trillions of bacteria that live in our bowels. These bacteria help to keep us healthy. Patients with liver cirrhosis have more 'unfriendly' bacteria than people without cirrhosis. Patients with cirrhosis also have a 'leaky' gut, allowing these bacteria to leak into the blood stream, potentially causing serious infections. These can usually be treated with antibiotics, but may be very severe and may also cause kidney failure, hepatic encephalopathy (a condition where toxins build up in the blood stream, causing confusion and even coma) and potentially life threatening bleeding.

The treatment proposed in our study, FMT (faecal microbiota transplantation,) takes the bacteria from the intestines of healthy volunteers and replaces the abnormal bacteria in the gut of a patient with cirrhosis. The aim of faecal microbiota transplantation (FMT) is to replace the 'unfriendly' bacteria with 'friendly' bacteria from healthy volunteers, therefore preventing them from getting into the blood stream and causing serious problems.
Volunteers are screened very thoroughly (in a similar way to blood donors) to ensure that they are healthy and not liable to pass on any infections to the recipients of the FMT.

FMT has been tested in a small group of patients with cirrhosis in America and was found to be safe, and has also been used more widely in other conditions such as patients with a gut infection called 'clostridium difficile' and patients with inflammatory bowel disease. It is extremely successful in these patients and we would like to see if it can be as successful in liver patients.

Results from this study would support further research into the benefit of using FMT to lessen infection rates in patients with liver cirrhosis who have developed bowel problems.

What groups of patients will the study involve?

We are interested in involving people in this study who have liver cirrhosis of any cause.

For peer review only - http://bmjopen.bmj.com/site/about/guidelines.xhtml

Why have I been invited?

All patients who are admitted to the Liver wards and/or seen in the Liver out-patient clinics at King's College Hospital NHS Foundation Trust who have liver cirrhosis may be eligible to participate in this study. This also includes patients that are awaiting consideration for or have already been listed for liver transplantation

Do I have to take part?

No, participation is entirely voluntary. It is up to you whether or not you wish to take part. If you decide to take part you will be given this information sheet to keep and asked to sign a consent form. If you decide to take part you are still free to withdraw at any time and without giving a reason. This will not affect the medical care you will receive.

Who is organising and funding this study?

The doctor in charge of this study is Dr Debbie Shawcross. The study is funded by the National Institute of Health Research and is being sponsored by King's College London and Kings College Hospital NHS Foundation Trust.

Who has reviewed the study?

All research in the NHS is looked at by an independent group of people, called a Research Ethics Committee, to protect your interests. This study has been reviewed and approved by the London South East Research Ethics Committee. It has also been approved by the Health Research authority and the Medicines and Healthcare products Regulatory Agency.

What will happen to me if I take part (SUMMARY)?

If you decide to take part you will be randomly assigned to one of two groups. One group (the 'active treatment' group) will receive Faecal Microbiota Transplantation (FMT) whilst the other group (the comparison or 'control group') will receive a 'placebo' treatment. Administration of placebo or FMT will take place only ONCE during this study.

The placebo treatment is a 'dummy' treatment given to participants in the control group of a clinical trial. This method using a 'placebo control' is used to make sure the study results are reliable by checking that any changes measured are actually due to the effect of the active drug and not just because of chance. The placebo in this trial is a solution of saline and glycerol (salty water and a preservative, which are the same ingredients used in the FMT itself, just without the FMT.)

Both groups will then be followed up to see how effective the active treatment is based on various measurements taken. Blood, urine, saliva and stool samples will be obtained for tests at four specific times. Firstly when starting the study at 'baseline' (just before the FMT or placebo is given,) and then repeated at 7, 30 and 90 day intervals after treatment has been administered. Follow-up appointments at the 7, 30 day and 90 days after starting will be arranged to coincide as far as possible with planned routine medical appointments.

For peer review only - http://bmjopen.bmj.com/site/about/guidelines.xhtml

King's College Hospital NHS
NHS Foundation Trust

Study procedures	Screening	Baseline (-7 days)	Endoscopy	Day 7 (+/- 5days)	Day 30 (+/- 7 days)	Day 90 (+/- 7 days)
Signed informed consent	X					
Eligibility Criteria	X					
Participant demographics	X					
Medical and surgical histories	**X**			X	X	X
Dietary questionnaire		X		X	X	X
Medication usage		X		X	X	X
Clinical examination		X		X	X	X
Blood sampling		X		X	X	X
Stool sampling		X		X	X	X
Saliva sampling		X		X	X	X
Urine sampling		X		X	X	X
Randomisation		X				
FMT/placebo administration			X			
Adverse events monitoring /Safety			X	X	X	X

As part of the study you will have an endoscopy (a camera test looking into your stomach/small bowel) to give you the FMT or the placebo treatment. This means that you will not have to drink the liquid as it is placed directly into the small bowel where it is needed, avoiding the stomach acid, which could stop it working. Before the endoscopy you will be given a medicine to clear your own gut bacteria called 'Moviprep.' This will give you loose bowel motions, which is a normal reaction to the Moviprep. This is essential to clear your own gut bacteria from the bowel to allow the FMT to work.

We will see you after the endoscopy at days 7, 30 and 90 to take blood, urine, saliva and faecal samples from you. We will also assess your general wellbeing and check that your medicines have not changed. Blood samples will usually be taken at the time of when you are having tests done as part of your routine clinical care, to reduce the number of times you have to have a needle in your arm and, where possible, samples will be taken from tubes already in your blood vessels to minimise discomfort to you.
We will ask you to collect the urine and stool samples in the morning of each study visit and bring this with you when you attend clinic. We will ensure you are given all the necessary collection pots for this. After the last visit at 90 days, you will return to the usual standard of care as you were having for you underlying condition.

More detailed information about the study visits can be found on page 7 of this information sheet.

For peer review only - http://bmjopen.bmj.com/site/about/guidelines.xhtml

What are the side effects of taking part?

If you are assigned to the active treatment group and receive FMT, there are some possible side effects that you should be aware of. Faecal transplants have not been widely used in patients with cirrhosis yet, but have been used in lots of patients with an antibiotic associated diarrhoea called 'clostridium difficile.' Small numbers of patients in these studies reported some abdominal distension/bloating, flatulence, diarrhoea or constipation as side effects of the treatment. Part of this study is to assess how well tolerated the treatment is and whether or not patients with cirrhosis experience similar symptoms.

Side effects of blood sampling can include bruising and it can be uncomfortable. However, every effort will be made to perform blood sampling at the same time as regular blood tests to minimise any discomfort. There should be no side effects of sampling your urine and stool as we will simply ask you to collect the samples when you go to the toilet as normal. Saliva will be collected by asking you to rinse your mouth out with water and to spit into a collection pot.

What are the possible disadvantages and risks of taking part?

We hope that the knowledge we would gain from this study will improve our understanding of the way in which FMT works, and the role of the gut and immune system in liver cirrhosis. Patients who received FMT in a small study in America did well and had fewer hospital admissions than those in the 'standard of care' group. FMT appears to be safe in the considerable numbers of patients who have received it for other reasons. There are no disadvantages or risks involved in taking part with regard to the samples being obtained, and the amount of blood taken will not be harmful to you. The placebo treatment is not harmful, but is not expected to have any benefit on your clinical condition as it contains no active drug or therapy. All patients, regardless of whether they receive placebo or FMT have the same rigorous follow up and support from the trials team.

You will be closely monitored after the treatment is given and will be seen at regular intervals afterwards to see if you have experienced any side effects. We do not anticipate any serious problems to occur after the FMT or placebo treatment, but you will be given contact numbers should you run into any problems outside of the trial visits.

Apart from the side effects of FMT, all patients (those on placebo and active treatment) will need to take bowel preparation, which can cause dehydration. The endoscopy itself also has a small risk of bleeding, perforation (this is very rare and is thought to occur in around only 1 in 2000 tests) and damage to teeth and dental work. There is also a risk of aspiration of the FMT or placebo into the lungs, therefore you will be monitored closely after the procedure to monitor for this.

What are the possible benefits of taking part?

You will be taking part in a new and innovative trial to assess the effect of FMT on cirrhosis. There are few treatments available to patients with cirrhosis, aside from liver transplantation. Unfortunately due to the scarcity of donor organs, even those listed for transplant can wait months or even years for a new liver. Others are too unwell or frail to be listed for transplant. We hope that looking at FMT in more detail may provide a much needed treatment for patients with cirrhosis who are not suitable for transplant or to help those awaiting a new liver. This study is designed to look at the safety and tolerability of the treatment to see if a larger study would show an improvement in wellbeing for these patients.

For peer review only - http://bmjopen.bmj.com/site/about/guidelines.xhtml

King's College Hospital NHS
NHS Foundation Trust

What if I lose the ability to provide consent after enrolling to the study ('loss of capacity)?

In the event that you lose the ability to provide ongoing consent during the research due to your illness, having been able to provide consent at the start of the study, we will approach an appropriate relative or friend on your behalf to ask them if they think that you would have wanted to continue participation in this study. This person is termed a 'Consultee' and they are asked to set aside their own views and provide advice on you continuing to participate in the research, taking into consideration your wishes and interests.

Any advance decisions you may have made, and that they are aware of, should take precedence. If the Consultee decides that you would have no objection to continue taking part they will be asked to read and sign a consultee declaration form. They will then be given a copy to keep. If they decide that you would not wish to continue taking part we will withdraw you from the study; this will not, in any way, affect the standard of care you receive. If the Consultee we approach does not feel able to take on this role they may want to identify someone else to do it. They will be asked to take time to decide whether or not they feel that you would wish to continue taking part. They will be encouraged to ask the research team if there is anything that is not clear or if they would like more information. The information they will be provided with is the same as that provided to you.

You may wish to identify a suitable Consultee at this stage given the above, so that if you do lose the ability to provide consent after you are enrolled (should you decide to participate in the study) we can approach a person of your choice.

What if something goes wrong?

If you have a concern about any aspect of this study, you should ask to speak to your study doctor who will do their best to answer your questions (contact details on page 8) If you remain unhappy and wish to complain formally, you can do this through the NHS Complaints procedure by contacting your local Patient Advice Liaison Service (PALS) office. Details of your local office can be obtained by asking your study doctor, GP, telephoning your local hospital or looking on the NHS choices website. http://www.nhs.uk/pages/home.aspx

Every care will be taken in the course of this study. However in the unlikely event that you are injured by taking part, compensation may be available.

In the event that something does go wrong and you are harmed during the research and this is due to someone's negligence then you may have grounds for a legal action for compensation against King's College Hospital NHS Foundation Trust & King's College London, but you may have to pay your legal costs.

Regardless of this, if you wish to complain, or have any concerns about any aspect of the way you have been approached or treated by members of staff or about any side effects (adverse events) you

For peer review only - http://bmjopen.bmj.com/site/about/guidelines.xhtml

may have experienced due to your participation in the study the normal National Health Service complaints mechanisms are available to you. Please ask your study doctor if you would like more information on this.

Will my taking part in this study be kept confidential?

Yes. Once you have consented to take part in the study, you will be given a unique study number, which will be used to identify any samples and information collected during the research.
Some information regarding you and your condition will be recorded as part of this study. We will not record any personal identifiable information (name, date of birth or contact details) as part of the research records. All information will be stored anonymously in a password-protected database, or in a file in a secure research office. Only your treating doctors and the research team will have access to this information.

We will inform your General Practitioner (GP) of your participation in the study - this is routine for this type of research and helps them to care for you during the trial. We advise that you inform your private medical insurance provider (if you hold private medical insurance) of your participation in this study - this again is routine procedure for this type of research.

What will happen to the results of the research study?

When we have results for an adequate number of patients after the study is completed we plan to publish them in an international journal so that the information can benefit as many people as possible. We can provide you with a brief summary of the results of the study when available should you desire this.

What will happen to any samples that I give?

Every effort will be made to ensure blood samples for the study are taken at the same time as samples are taken as part of your routine clinical care – so that there will be no additional discomfort to you as a result of being involved in this study. The amount of blood taken per time point for the study is approximately 50mls or 10 teaspoonfuls. This is a small amount for the body (which contains about 5L of blood, or 5000mL) and will not adversely affect you in any way.

Samples of blood and other fluids obtained may be used immediately or saved for human or bacterial DNA or immune system analysis in the future, in the Liver Biobank. Some of the actual tests on the samples that are obtained as a result of you taking part in the study will be performed on site at King's College Hospital NHS Foundation Trust, whilst some samples will be transferred to other collaborator laboratories in the UK and France. At all times any samples that are transferred and stored will be coded anonymously regardless of which laboratory they are sent to.

Contact details:

Thank you for taking the time to read this information.

If there is any other information you would like, please do not hesitate to contact us on the numbers below. Out of hours or if a response on the above contact number is unavailable, it is possible to contact the on-call Hepatology Registrar – via KCH Switchboard – who will pass any messages on to the senior medical staff involved.

For peer review only

For peer review only - http://bmjopen.bmj.com/site/about/guidelines.xhtml

King's College Hospital NHS
NHS Foundation Trust

Principal investigator: Dr Debbie Shawcross Tel: 02032992504
Clinical research fellow: Dr C Woodhouse Tel: 02032992504
Research nurse: Ane Zamalloa Tel: 0203299 7623

For peer review only

For peer review only - http://bmjopen.bmj.com/site/about/guidelines.xhtml

King's College Hospital NHS
NHS Foundation Trust

What will happen to me if I take part (DETAILED EXPLANATION)?

You will be assessed for your eligibility to be involved in this study, and if you are happy to proceed will sign the consent form with a member of the research team. You will then be randomly allocated to one of two treatment groups:

1) the 'active treatment' group: participants in this group will receive the treatment being tested (FMT), or
2) the comparison or 'control group'; participants in this group will receive a 'placebo' treatment, which is a 'dummy' treatment and is indistinguishable from the actual treatment given to participants in the 'active treatment' group.

Before starting treatment with either of the above, you will undergo baseline assessments, which involve the following (those marked with * are being done as part of the study):

- Medical history including current medication usage.
- Physical examination including blood pressure, pulse rate, height, weight.
- Dietary questionnaire* and quality of life questionnaire*
- Blood sampling: will include tests of blood counts, kidney and liver function performed as part of your routine clinical care as well as checks for various viruses that can affect the liver.
- Blood sampling*: to measure endotoxin levels, immune function including DNA analysis and metabolic profiling.
- Urine sampling*: we will ask you to provide a urine sample (approx. 20mls) to measure metabolic profiling. If you are a woman of child-bearing age, we will also do a pregnancy test.
- Stool sampling*: we will ask you to provide stool samples to measure what types of bacteria are found, and in what patterns and quantities.
- Salivary sampling: *: we will ask you to provide saliva samples from your mouth to measure what types of bacteria are found, and in what patterns and quantities.
- If you have fluid in your abdomen called "ascites" we would like to take a sample of the fluid to check for infection. This involves passing a small needle into your abdomen under aseptic conditions. This test is voluntary and you do not have to take part, but it would help us to check for infection in the fluid and would mean we could treat any infection early, should one be present*.

After the baseline assessments have been completed, you will come up to the endoscopy unit to have the FMT or placebo treatment given. The procedure will be explained to you and the risks and benefits discussed. Endoscopy is a safe test, but like all procedures has some risks. The main risks to be aware of are a small risk of bleeding and the risk of perforation (1 in 9000.) There is also a risk of damaging teeth or dental work and a small risk of 'aspiration' which is where fluid from the stomach goes into the lungs. This is why we ask you not to eat for six hours prior to the test, to make sure the stomach is empty. Some people also develop a sore throat after the procedure. This should get better on its own.

You will also be given a medicine prior to the test called 'Moviprep' to help clear your bowels of their usual bacteria prior to the treatment with FMT or placebo. This is a safe medicine, but will cause you to have loose stools. This is essential to its function, but it is important you drink

For peer review only

plenty of fluid to prevent dehydration. Side effects can include dehydration, nausea and vomiting.

You will take one sachet of Moviprep mixed with a litre of water at 19.00 the night before the test. You can then have a light meal. On the morning of the endoscopy you will drink another sachet of Moviprep mixed with one litre of water at around 7.00am. You can take your usual medications as normal. There is a lot of fluid to drink, so you may find it easier if it is chilled or mixed with a squash (not blackcurrant) to make it easier to drink.

You will undergo similar assessments to the baseline visit at 7, 30 and 90 day intervals as you did just before starting treatment. You will in addition undergo the following as part of the study:

- Study treatment adverse events monitoring*: this to check whether you may have experienced any side effects that may be due to the study medication.

Your last visit will be at 90 days from the start of the trial. Samples will be collected and you will be reviewed by the team to check for any side effects. You will have the opportunity to ask questions to the team at all visits throughout the duration of the study and if you have any concerns outside of these visits you will be told who to contact.

Thank you for taking the time to read this information. If there is any other information you would like, please do not hesitate to contact us (020 3299 9000 ext. 32504).

Dr C Woodhouse, Dr V. C. Patel, Professor A Sanchez-Fueyo, Dr D. Shawcross
Institute of Liver Studies, Kings College Hospital

For peer review only

For peer review only - http://bmjopen.bmj.com/site/about/guidelines.xhtml

BMJ Open

PROFIT: A PROspective, randomised placebo controlled feasibility trial of Faecal mIcrobiota Transplantation in cirrhosis: study protocol for a single-blinded trial

Journal:	*BMJ Open*
Manuscript ID	bmjopen-2018-023518.R1
Article Type:	Protocol
Date Submitted by the Author:	18-Sep-2018
Complete List of Authors:	Woodhouse, Charlotte; King's College London, Hepatology<br>Patel, Vishal; Kings College London, Hepatology<br>Goldenberg, Simon; Guy's & St. Thomas' NHS Foundation Trust and King's College,<br>Sanchez Fueyo, Alberto; Kings College London, Hepatology<br>China, Louise; UCL, Division of Medicine<br>O'Brien, Alastair; UCL, Division of Medicine<br>Flach, Clare; 1. School of Population Health & Environmental Sciences, Faculty of Life Sciences & Medicine, King's College London<br>Douiri, Abdel; 1. School of Population Health & Environmental Sciences, Faculty of Life Sciences & Medicine, King's College London<br>Shawcross, Debbie ; Kings College London, Liver Sciences, 1st Floor JBC, School of Immunology & Microbial Sciences Faculty of Life Sciences & Medicine, King’s College London, Denmark Hill Campus, London, SE5 9RS
**Primary Subject Heading**:	Gastroenterology and hepatology
Secondary Subject Heading:	Pharmacology and therapeutics
Keywords:	cirrhosis, faecal microbiota transplantation, gut microbiota, feasibility

SCHOLARONE™
Manuscripts

PROFIT: A PROspective, randomised placebo controlled feasibility trial of Faecal mIcrobiota Transplantation in cirrhosis: study protocol for a single-blinded trial

C. Woodhouse[1], V. Patel[1], S. Goldenberg[2], A. Sanchez-Fueyo[1], L. China[3], A. O'Brien[3], C. Flach[4], A. Douiri[4], D. Shawcross[1]

1. **Liver Sciences, 1st Floor James Black Centre, School of Immunology & Microbial Sciences, Faculty of Life Sciences & Medicine, King's College London, Denmark Hill Campus, London, SE5 9RS**
2. **Directorate of Infection, Guy's and St Thomas' NHS Foundation Trust, London**
3. **Division of Medicine, University College London (UCL), London**
4. **School of Population Health & Environmental Sciences, Faculty of Life Sciences & Medicine, King's College London**

Corresponding author: Dr Charlotte Woodhouse, Liver Sciences, 1st Floor James Black Centre, School of Immunology & Microbial Sciences, Faculty of Life Sciences & Medicine, King's College London, Denmark Hill Campus SE5 9RS charlottewoodhouse@nhs.net Tel 02032993713 Fax: 020232993899

For peer review only

Abstract

Introduction

Patients with advanced cirrhosis have enteric bacterial dysbiosis and translocation of bacteria and their products across the gut epithelial barrier. This culminates in systemic inflammation and endotoxemia, inducing innate immune dysfunction which predisposes to infection, and development of complications such as bleeding, sepsis and hepatic encephalopathy (HE). This feasibility study aims to assess the safety of administering faecal microbiota transplant to patients with cirrhosis and explore the effect of the intervention on their prognosis by achieving restoration of a healthy gut microbiome.

Methods and Analysis

PROFIT is a single-centre, randomized, single-blinded, placebo-controlled study evaluating Faecal Microbiota Transplantation (FMT) against placebo. Patients with advanced but stable cirrhosis with a MELD score between 10 and 16 will be recruited. Twenty-four patients will be randomized to FMT plus standard of care (as per our institutional practice) and eight patients to placebo in a ratio of 3:1. Patients will be evaluated at baseline before the study intervention is administered and at 7, 30 and 90 days post intervention to assess safety and adverse events. FMT/placebo will be administered into the jejunum within 7 days of baseline. Research Ethics approval was given by the London South East Research Ethics committee (ref 17/LO/2081).

The primary outcome measure will be safety and feasibility as assessed by recruitment rates, tolerability and safety of FMT treatment. Results will be disseminated via peer reviewed journals and international conferences. The recruitment of the first patient occurred on 23/05/2018.

The study has been registered with *ClinicalTrials.gov*– reference number: NCT02862249. The trial is registered with the European Medicines Agency (EudraCT 2017-003629-13) and has been adopted by the NIHR (IRAS 197237). This manuscript refers to the version 2.0 of the protocol; pre-results.

Strengths and limitations of this study

- This study is powered to assess feasibility and safety of administering FMT to patients with cirrhosis, however it is not statistically powered to assess for clinically relevant outcomes.
- This is the first study examining the effect of FMT delivered directly into the small bowel in patients with advanced cirrhosis. This trial does not involve antibiotic pre-treatment in the FMT group, as has been undertaken in the USA in patients with HE.
- PROFIT will assess instillation of FMT/placebo directly into the small bowel, as opposed to the colon-, directly targetting small bowel bacterial overgrowth that is observed in cirrhosis
- A limitation of the study is its single-blinded design, which was necessary as the FMT and placebo (saline with glycerol) are not matched.

Introduction

Patients with advanced cirrhosis have enteric dysbiosis with small bowel bacterial overgrowth and translocation of bacteria and their products across the gut epithelial barrier (1). This culminates in systemic inflammation and endotoxaemia, inducing innate immune dysfunction which predisposes to infection (2), and development of complications such as bleeding, sepsis and hepatic encephalopathy (3). It also plays a key role in the natural history of cirrhosis by influencing the rate of progression to advanced liver disease and terminal liver failure (4).

Utilising quantitative metagenomics our group has found 75,245 genes differentially expressed between patients with cirrhosis and healthy individuals. Over 50% of these bacterial species are of buccal origin suggesting an invasion of the gut from the mouth in cirrhosis (5). Patients with cirrhosis also have salivary dysbiosis associated with impaired salivary defenses and systemic inflammation. Salivary dysbiosis has been shown to be greater in patients with cirrhosis who developed complications necessitating hospitalisation within 90 days. Modulating the gut microbiota in patients with cirrhosis with the non-absorbable antibiotic rifaximin has been associated with improved cognitive performance and reduction in endotoxaemia in patients with cirrhosis (6, 7). Moreover, we have recently performed a multi-centre retrospective study including 170 patients in which rifaximin-α therapy given for 90 days significantly (i) reduced hospital re-admission rates after 3 months treatment, impacting significantly on the NHS resource burden and (ii) reduced overall liver

For peer review only

disease severity (as measured by the Child Pugh and Model for End Stage Liver Disease scores) raising the possibility that modulation of gut microbiota may significantly modify the natural history of chronic liver failure (8).

These data constitute in our view "proof of principle" that modifying the gut microbiota in patients with cirrhosis improves clinical outcomes. Rifaximin-α was approved by NICE for the prevention of the recurrence of overt hepatic encephalopathy in cirrhosis (9) but considerable concern remains regarding whether long term antibiotic prescription will result in a change in bacterial function and virulence rather than a simple reduction in bacterial population and whether this may drive bacterial resistance to antibiotics in an already functionally immunocompromised population. The question was therefore raised as to whether directly, as opposed to indirectly modulating the gut microbiota utilising faeces from healthy donors may be a safer and more durable therapy. Faecal microbiota transplantation (FMT) has been licensed by NICE since 2014 for the treatment of recurrent *Clostridium difficile* infection (10). FMT has shown promising results in clinical trials of several disease states resulting from gut dysbiosis beyond *Clostridium difficile* infection (10) for example in ulcerative colitis (11-14).

We hypothesise that in patients with advanced cirrhosis, FMT may reduce the progression to chronic liver failure including the development of jaundice, ascites, bleeding, encephalopathy, infection and organ dysfunction. Whether FMT is feasible in the setting of cirrhosis remains to be investigated. We propose conducting a feasibility trial to determine whether FMT from a healthy donor will alleviate gut dysbiosis and immune dysfunction in advanced cirrhosis.

Methods and Analysis

Primary Objectives:

The primary objective of this study will be to assess whether stabilising gut dysbiosis with FMT in patients with advanced cirrhosis is both feasible and safe.

Primary Endpoints:

The primary endpoints of the study will be twofold. To assess the feasibility of FMT as determined by the recruitment rates (including acceptability of the intervention) and tolerability of FMT e.g. gastro-oesophageal reflux rates. The second primary outcome measure will be to assess the safety of FMT administration, including the incidence of any transmissible bacterial or viral infection that is deemed to have been acquired from the donor including *Clostridium difficile* infection.

Secondary Objectives:

The secondary objectives of the study are to provide preliminary evidence of efficacy for a larger randomised trial, with the purpose of choosing the optimal primary outcome, and

For peer review only

estimating the parameters for sample size calculation. We will also collect blood, stool and urine samples from participants to assess the stability of the transplanted gut microbiome by comparing the percentage composition of the stool microbiota on day 7, 30 and 90 with the donor microbiome. Plasma endotoxin (and endotoxin binding protein), pro- and anti-inflammatory cytokine levels, bacterial DNA quantification and serum procalcitonin will be performed at 7, 30 and 90 days as will changes in faecal biomarkers (calprotectin, lactoferrin and M2-Pyruvate Kinase).

Trial Design

PROFIT is a single-centre study. Thirty-two patients will be recruited from out-patient clinics at King's College Hospital (KCH) or from suitable inpatients on the wards. The patients will be recruited as per the inclusion and exclusion criteria listed in table 1. Patients will be randomized in a single-blinded fashion in a ratio of 3:1 FMT to placebo. Patients will be unaware of the intervention given, but investigators will not be blinded to the treatment intervention.

Patient and Public Involvement

A lay reviewer/patient representative at the NIHR reviewed the protocol. This was funded by the Research for Patient Benefit programme. Feedback was taken on board and revisions were subsequently made.

The British Liver Trust, British Society of Gastroenterology Liver Research Group and King's Liver Outpatient Advisory Group (patient group) were all involved in the study set-up and protocol development at all stages.

Results will be disseminated to study participants via the Liver Research Nurse if they indicate an interest in the study outcome.

Table 1- Inclusion and exclusion criteria

Inclusion Criteria

- 18–75 years
- Confirmed advanced cirrhosis of any aetiology with a MELD (15) score between 10 and 16. The diagnosis of cirrhosis will be based on clinical, radiological, or histological criteria.
- Patients with alcohol-related liver disease must have been abstinent from alcohol for a minimum of 6 weeks.
- Patients must be deemed to have capacity to consent to the study.

For peer review only

Exclusion Criteria

- Severe or life-threatening food allergy
- Pregnancy or breastfeeding
- Patients treated for active variceal bleeding, infection, bacterial peritonitis, overt hepatic encephalopathy or acute-on-chronic liver failure within the past 14 days.
- Patients who have received antibiotics in the past 14 days.
- Active alcohol consumption of >20 grams/day.
- Has had a previous liver transplant
- Hepatocellular carcinoma outside of the Milan Criteria (16)
- Inflammatory bowel disease
- Coeliac disease
- A history of prior gastrointestinal resection such as gastric bypass
- Patient is not expected to survive the duration of the study (90 days).
- Severe renal impairment (creatinine >150 µmol/L)
- HIV positive
- Immunosuppression e.g. more than two weeks treatment with corticosteroids within 8 weeks of intervention, active treatment with tacrolimus, mycophenylate, azathioprine.

Patient Population

The study will include all patients aged 18 to 75 years with capacity to consent and a diagnosis of cirrhosis. Patients will have a MELD (15) score of between 10 and 16. The diagnosis of cirrhosis may be based on radiological, clinical or histological parameters. In the case of alcohol-related cirrhosis, patients must have been abstinent for a minimum of six weeks. The exclusion criteria are outlined in table 1. Anticipated recruitment is outlined in Figure 1 below (17).

Consent

All participants will be asked to provide informed consent after having had the opportunity to discuss the trial with the clinician, their family and friends and having read the patient information sheet (PIS- see supplementary file.) Patients who lack capacity will not be enrolled in this study. If patients lose capacity during the trial follow up period, an appropriate legal representative will be consulted and will provide consent to ongoing trial participation after appropriate discussion with the trials team and having read the PIS. If the legal representative does not agree to ongoing trial participation, the subject will be withdrawn from trial follow up.

Figure 1 Study Flow Chart and Anticipated Recruitment

For peer review only

Study Intervention

FMT is prepared in a laboratory at St Thomas' Hospital in accordance with the principles of Good Manufacturing Practice (GMP) and under Manufacturing Authorisation for an Investigational Medicinal Product (MIA(IMP)) from the Medicines and Healthcare products Regulatory Agency (MHRA). FMT donors are healthy volunteers with no medical problems and normal body mass index. They must not be taking any regular medications and are rigorously screened for blood borne and enteric pathogens prior to donation. Donors undergo questionnaire screening for risk factors at baseline and again at donation to reduce the risk of transmissible infection as outlined in table 2 (full questionnaire in supplementary materials). Stool is processed (diluted and filtered with the addition of glycerol) within 6 hours of donation and stored at -80 degrees Celsius for up to six months (as previously described) (18). Material for FMT has full traceability from donor to recipient and aliquots of donor stool are kept for 30 years to allow further testing in the case of any adverse events. Placebo is a solution of saline with glycerol without faecal matter.

Blood (serology)

- HIV 1+2 serology
- HTLV I/II Ab
- Hepatitis A IgG (and if positive IgM)
- Hepatitis B surface antigen (HBsAg) and core antibody
- Hepatitis C (HCV Ab)
- Hepatitis E
- Syphilis
- CMV/EBV IgG/M
- Strongyloides stercoralis (ELISA)

Stool

- PCR for gastroenteritis agents (*Campylobacter, Salmonella, Shigella* and *E. coli* O:157)
- Ova, cysts and parasites by concentration and microscopy x3
- *C. difficile* test
- Norovirus PCR
- Screen for gentamicin and carbapenem resistant Gram negative organisms
- Screen for MRSA
- Helicobacter pylori antigen
- Entamoeba histolytica PCR

Table 2 blood and stool testing of donor FMT samples

For peer review only

Following identification, all patients will attend a screening visit and sign the informed consent form. If deemed to be eligible they will attend for a baseline visit with full clinical history and examination. Medication history will be recorded. Patients will complete a dietary questionnaire. Baseline samples of blood, urine, saliva and stool will be obtained. All patients will subsequently attend for a gastroscopy at which the FMT or placebo will be administered under direct visualization into the jejunum via a nasojejunal tube. This will be performed as per the local Endoscopy Unit Protocol using topical local anaesthetic spray or midazolam sedation as per patient preference. Patients will first be asked to take bowel preparation to purge the bowel of its native bacteria. This consists of two sachets of Moviprep® taken prior to gastroscopy. Patients will be monitored for side effects in the Clinical Research Facility after the procedure.

Evaluations during and after treatment

Participants will be reassessed at 7 (+/- 5), 30 (+/-7) and 90 (+/-7) days post intervention. They will be reviewed in the Clinical Research Facility and undergo physical examination, review of medications and dietary changes and adverse event monitoring. Samples of blood, urine, saliva and stool will be taken at these visits also. At the end of the 90 day follow up period, patients will return to their usual care pathway.

Statistical analysis

Sample Size

This feasibility study will evaluate feasibility parameters using 95% Confidence Intervals. The sample size has been proposed mainly to enable the trial to be conducted within the allocated budget and with acceptable precision for continuous outcomes. According to the simulation work by Teare MD et al. (18), even with the relatively small pilot sample size of 20, the planned studies would have at least 80% power to detect the target effect size (for continuous outcomes) more than 75% of the time. Teare et al. recommend that 60 to 100 subjects are sufficient to estimate an event rate (such as recruitment rates) with acceptable precision in a feasibility study, while sample sizes between 24 and 50 have been recommended for the accurate estimation of standard deviations. Therefore, we have chosen a sample size of 32 patients in this trial to have reliable data on all critical parameters (including event rates) which can also be utilised when planning a larger intervention trial. For event rates (e.g. recruitment rates) and particularly in the extreme

For peer review only - http://bmjopen.bmj.com/site/about/guidelines.xhtml

case with lower rates e.g. 10%, we estimate a drop of precision by only 5% using our updated sample size and the minimum recommended by Teare et al. (0.16 for 60 patients vs 0.21 for 32 patients). Figure 2 below illustrates the reduction in precision of different rates when the sample size increases for binary outcomes. This sample size will also be feasible within the budget and will provide acceptable information for planning a future large clinical trial.

Figure 2 Confidence interval width around one proportion (P) by sample size (N) from PASS software REF - PASS 15 Power Analysis and Sample Size Software (2017). NCSS, LLC. Kaysville, Utah, USA, ncss.com/software/pass.

The sample size of 32 patients will undergo randomisation in a 3:1 ratio. This will allow the study to demonstrate feasibility of randomising, yet providing robust evidence with respect to the feasibility of the treatment, and preliminary evidence of efficacy parameters.

The following feasibility criteria have been established:

- 25% of screened patients will consent: 256 patients will allow the estimate of the 2-Sided 95% confidence interval of the proportion of consented patients, where the distance from the observed proportion to the limit is 0.058 units.
- 50% of patients screened will fulfil inclusion/exclusion criteria for the trial: screening 64 patients will allow the estimate of the 2-Sided 95% confidence interval of the proportion of patients eligible, where the distance from the observed proportion to the limit is 0.128 units.
- 80% of randomised patients will complete treatment and follow up: 26 of 32 patients completing will allow the estimate of the 2-Sided 95% confidence interval for a single proportion, where the distance from the observed proportion to the limit is 0.177 units.

Differences between the placebo and treatment group in efficacy binary outcomes, will be estimated along with the two-sided 95% CI. Assuming the proportion with the outcome in the control group is 0.5 our sample size will allow estimation of the half-width of the confidence intervals for different size of differences as indicated below. Differences of 0.4 or higher will be distinguishable from 0.

Table 3 Half width of confidence intervals for differences in proportions between two groups with given sample size, placebo group p=0.5 (created with PASS software REF - PASS 15 Power Analysis and Sample Size Software (2017). NCSS, LLC. Kaysville, Utah, USA, ncss.com/software/pass.)

Difference	0.1	0.2	0.3	0.4	0.5
Width	0.398	0.392	0.382	0.337	0.347

The half-width of the 95% CI of the difference in means for continuous outcome will be 0.889 SD.

For peer review only

Clinical Endpoints

Clinical and safety events will be listed and summarised by intervention group. MELD (15) scores will be calculated by visit and treatment group.

Data synthesis, analysis and presentation

Feasibility and efficacy outcomes will be summarised using the appropriate descriptive statistics, and 95% Confidence Intervals will be calculated to allow for success and go/no go decisions. Biomarker data will be pre-processed according to the established standards for each platform, and statistical analyses will be performed using non-parametric and permutation based methods which are more appropriate for small sample sizes.

Statistical Software

Analyses will be performed using R and/or Stata statistical software packages.

Discussion

The PROFIT study is the first study to look at the use of FMT delivered directly into the small bowel in patients with cirrhosis. Liver transplantation is a highly selective treatment and only those most likely to benefit will be listed. Depending on blood type patients can wait up to 24 months for a liver transplant. There are even fewer options for patients who are unsuitable for transplantation.

Cirrhosis is often complicated by recurrent admissions with sepsis, variceal bleeding, ascites and hepatic encephalopathy. We know that patients with cirrhosis have enteric dysbiosis and altered gut permeability. The non-absorbable antibiotic rifaximin has proved useful in patients with cirrhosis and chronic hepatic encephalopathy, but the long-term benefits of antibiotic use are unknown and the possibility of development of bacterial resistance remains with all antibiotics. The possibility of re-populating the gut of cirrhotic patients with healthy gut microbiota could be a potential alternative, providing a new treatment approach for these vulnerable individuals.

A group in the USA have recently published the results of the impact of FMT delivered via enema in 10 cirrhotic patients, compared with 10 patients receiving standard of care treatment (19). FMT was found to be safe and the patients in the treatment group did not have any admissions for HE, whereas there were six episodes of HE in the standard of care group. Four of these episodes required hospital admission. The FMT group were treated with broad spectrum antibiotics prior to FMT administration, whereas the standard of care group were not, meaning that the effects of FMT cannot be clearly separated from the antibiotic administration. We hope that PROFIT will add to the knowledge base of the use of FMT in cirrhosis and will provide an accurate assessment of the safety of FMT. We plan to treat the FMT and control groups identically, aside from the administration of FMT to accurately assess its impact, without the confounding factor of antibiotic treatment.

For peer review only

A limitation of our study is the single-blinded design. This was selected due to the inherent difficulties in preparing a matched placebo, without introducing substances that may upset the delicate gut microbiota. The IMP will be delivered by the trial investigators, so it has not been possible to blind the clinicians in this study. Patients and the trial statistician will be blinded.

The study is not powered to detect differences in clinical outcomes, but may provide evidence for markers relating to clinical outcomes that could be studied in a larger RCT.

Sponsor

The PROFIT trial is co-sponsored by King's College Hospital and Kings' College London. The funding was supplied by an NIHR research grant for patient benefit (PB-PG-0215-36070).

Trial Monitoring Groups

Trial Steering Committee (TSC)

This group will oversee the running of the trial and discuss any issues that may arise throughout the process of recruitment and follow up of patients. The group will be chaired by an independent clinician. Investigators will report to the group on a regular basis. The Data Monitoring Committee (DMC) will inform the TSC if there are any issues raised from its discussions.

Data Monitoring Committee

This is an entirely independent group that analyzes interim data, to determine whether or not the trial is safe to continue. It monitors adverse events and adverse reactions and reacts to any issues and directs the TSC as to whether or not the trial should continue. The DMC undertakes interim statistical analysis using an independent statistician to ensure the ongoing safety and integrity of the trial. The members of this committee are independent of the trial, but will be experienced clinicians with expertise in clinical trials.

Ethics and Dissemination

The ethical permission has been given by the South-East Research Ethics Committee, REC reference 17/LO/2081, IRAS project ID 197237. EUDRACT number 2017-003629-13.
The results of the trial will be analyzed and published in a peer reviewed journal and disseminated at international conferences.

Funding statement

This work was supported by the NIHR, grant number PB-PG-0215-36070. This paper presents independent research funded by the National Institute for Health Research (NIHR) under its Research for Patient Benefit (RfPB) Programme (Grant Reference Number PB-PG-

For peer review only

0215-36070). The views expressed are those of the author(s) and not necessarily those of the NHS, the NIHR or the Department of Health & Social Care.

Competing Interests Statement

Dr Shawcross has received fees from Norgine, Falk and Shinogi-, outside the submitted work. Dr Goldenberg reports grants and personal fees from Astellas, personal fees from MSD, personal fees from Pfizer, personal fees from Shinogi, outside the submitted work.

Acknowledgements

We would like to thank the clinicians involved, King's College Hospital R&D Department, the NIHR, patient advisers and lay reviewers for their contributions in the set-up of the PROFIT trial. We would also like to acknowledge Liz Allen, our QP, for her contribution to the MHRA approval process.

Author Contributions

CW and DS wrote the manuscript, with assistance from SG, VP and ASF. AD and CF prepared the statistical analysis plan. LC and AO'B advised on study analyses and the format of the protocol for submission. SG provided the expert advice on FMT and has set up the FMT service at GSTT, which will supply the FMT to the study. All authors reviewed and edited the manuscript prior to submission.

For peer review only

1. Wiest R, Garcia-Tsao G. Bacterial translocation (BT) in cirrhosis. Hepatology. 2005;41(3):422-33.
2. Jalan R, Fernandez J, Wiest R, Schnabl B, Moreau R, Angeli P, et al. Bacterial infections in cirrhosis: a position statement based on the EASL Special Conference 2013. J Hepatol. 2014;60(6):1310-24.
3. Bajaj JS, Heuman D. M., Hylemin P.B. Sanyal A. J., White M. B. <Altered profile of human gut microbiome is associated with cirrhosis and its complications>. Journal of Hepatology. 2014;60:940-7
.
4. Nolan JP. The role of intestinal endotoxin in liver injury: a long and evolving history. Hepatology. 2010;52(5):1829-35.
5. Qin N, Yang F, Li A, Prifti E, Chen Y, Shao L, et al. Alterations of the human gut microbiome in liver cirrhosis. Nature. 2014;513(7516):59-64.
6. Bajaj JS, Heuman DM, Sanyal AJ, Hylemon PB, Sterling RK, Stravitz RT, et al. Modulation of the metabiome by rifaximin in patients with cirrhosis and minimal hepatic encephalopathy. PLoS One. 2013;8(4):e60042.
7. Shawcross DL. Is it time to target gut dysbiosis and immune dysfunction in the therapy of hepatic encephalopathy? Expert Rev Gastroenterol Hepatol. 2015;9(5):539-42.
8. Patel V OJ, Sturgeon J, Habtemariam Z, Preedy H, Richardson P, et al. Rifaximin Is Efficacious In The Treatment Of Chronic Overt Hepatic Encephalopathy: A UK Liver Multi-Centre Experience [Abstract]Suppl 1 Gut 2014;63.
9. NICE. National Institute for Health and Care Excellence. Rifaximin for the maintenance treatment of hepatic encephalopathy. 2015 Mar. Report No.: ID496.
.
10. NICE. National Institute for Health and Care Excellence. Faecal microbiota transplant for recurrent Clostridium difficile infection. 2014 Mar. Report No.: Interventional Procedure Guidance 485.
11. Sudarshan Paramsothy MAK, Nadeem O Kaakoush, Alissa J Walsh, Johan van den Bogaerde, Douglas Samuel, Rupert W L Leong, Susan Connor, Watson Ng, Ramesh Paramsothy, Wei Xuan, Enmoore Lin, Hazel M Mitchell, Thomas J Borody. <Multidonor intensive faecal microbiota transplantation for active UC; a randomsied placebo controlled trial.pdf>. Lancet. 2017;389:1218-28.
12. Paramsothy S, Paramsothy R, Rubin DT, Kamm MA, Kaakoush NO, Mitchell HM, et al. Faecal Microbiota Transplantation for Inflammatory Bowel Disease: A Systematic Review and Meta-analysis. J Crohns Colitis. 2017;11(10):1180-99.
13. Rossen NG, Fuentes S, van der Spek MJ, Tijssen JG, Hartman JH, Duflou A, et al. Findings From a Randomized Controlled Trial of Fecal Transplantation for Patients With Ulcerative Colitis. Gastroenterology. 2015;149(1):110-8 e4.
14. Moayyedi P, Surette MG, Kim PT, Libertucci J, Wolfe M, Onischi C, et al. Fecal Microbiota Transplantation Induces Remission in Patients With Active Ulcerative Colitis in a Randomized Controlled Trial. Gastroenterology. 2015;149(1):102-9 e6.
15. Kamath PS, Wiesner RH, Malinchoc M, Kremers W, Therneau TM, Kosberg CL, et al. A model to predict survival in patients with end-stage liver disease. Hepatology. 2001;33(2):464-70.

For peer review only

16. Mazzaferro V RE, Doci R, Andreloa S, Pulvirenti A, Bozzetti F, Montalto F, Ammatuna M, Morabito A, Gennari L Liver transplantation for the treatment of small hepatocellular carcinomas in patients with cirrhosis. New England Journal of Medicine. 1996;334(11):693-700.
17. DL S. RANDOMISED CLINICAL FEASIBILITY TRIAL PROTOCOL PROFIT: A PROspective, randomised placebo controlled feasibility trial of Faecal mIcrobiota Transplantation in cirrhosis
. 2015.
18. Goldenberg SD BR, Beales I, Digby-Bell J, Irving P, Kellingray L, Narbad A, Franslem-Elumogo N. Comparison of different strategies for providing fecal microbiota transplantation to treat patients with recurrent Clostridium difficile infection in two English hospitals: a review. Infectious Diseases and Therapy 2018.
19. Bajaj JS, Kassam Z, Fagan A, Gavis EA, Liu E, Cox IJ, et al. Fecal Microbiota Transplant from a Rational Stool Donor Improves Hepatic Encephalopathy: A Randomized Clinical Trial. Hepatology. 2017.

For peer review only

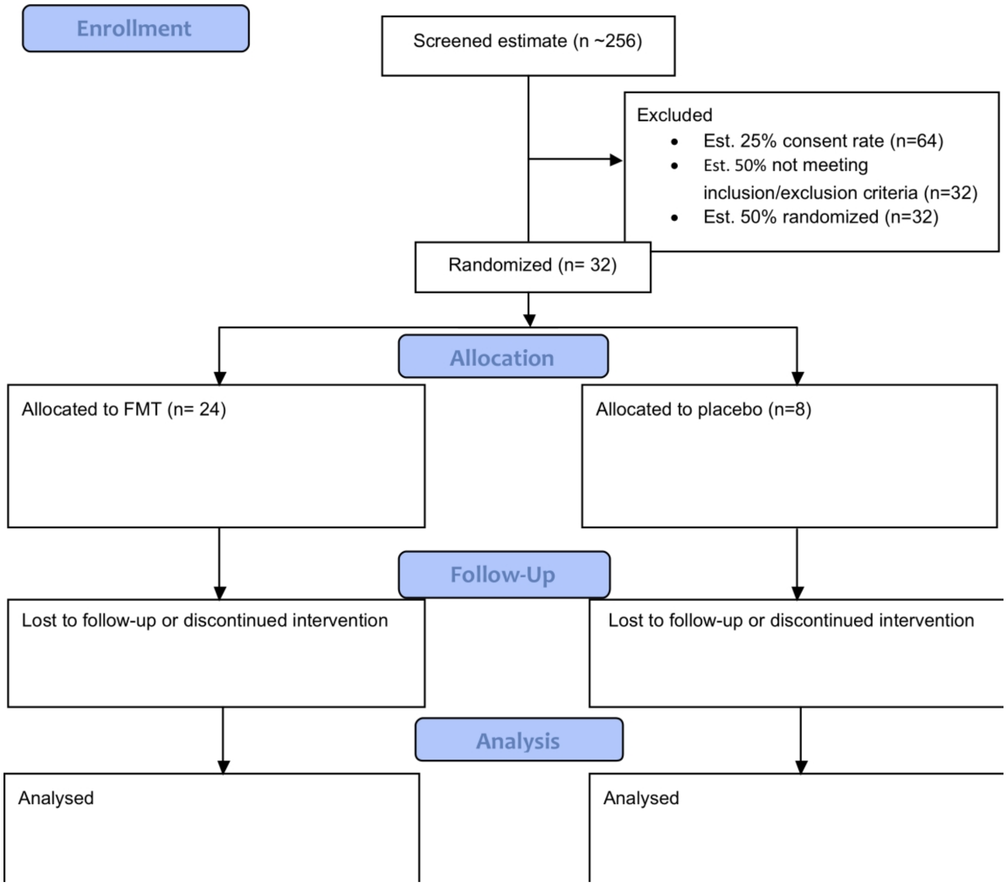

Figure 1 Study Flow Chart and Anticipated Recruitment

190x212mm (300 x 300 DPI)

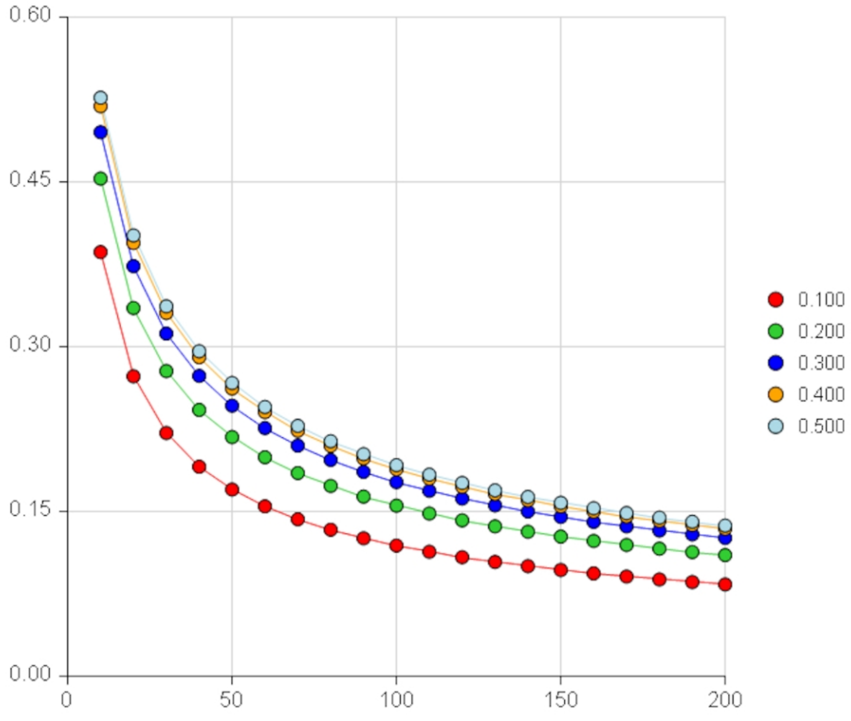

Figure 2 Confidence interval width around one proportion (P) by sample size (N) from PASS software

101x90mm (300 x 300 DPI)

King's College Hospital NHS
NHS Foundation Trust

Participant Information Sheet

Short Title: **PROFIT**

Full Title of Study: **A Prospective, randomized placebo controlled feasibility trial of faecal microbiota transplantation in cirrhosis**

Chief Investigator: **Dr D. Shawcross**

Researchers: **Dr C Woodhouse, Dr V. C. Patel, Prof A. Sanchez-Fueyo,**

For peer review only

Introduction

You are invited to take part in this research study. Before you decide it is important for you to understand why the research is being done and what it will involve. Please take time to read the following carefully and discuss it with friends, family or your GP if you wish. Ask us if there is anything that is not clear or if you would like more information. Take time to decide whether or not you wish to take part.

What is the purpose of the study?

Our bodies contain trillions of bacteria that live in our bowels. These bacteria help to keep us healthy. Patients with liver cirrhosis have more 'unfriendly' bacteria than people without cirrhosis. Patients with cirrhosis also have a 'leaky' gut, allowing these bacteria to leak into the blood stream, potentially causing serious infections. These can usually be treated with antibiotics, but may be very severe and may also cause kidney failure, hepatic encephalopathy (a condition where toxins build up in the blood stream, causing confusion and even coma) and potentially life threatening bleeding.

The treatment proposed in our study, FMT (faecal microbiota transplantation,) takes the bacteria from the intestines of healthy volunteers and replaces the abnormal bacteria in the gut of a patient with cirrhosis. The aim of faecal microbiota transplantation (FMT) is to replace the 'unfriendly' bacteria with 'friendly' bacteria from healthy volunteers, therefore preventing them from getting into the blood stream and causing serious problems.
Volunteers are screened very thoroughly (in a similar way to blood donors) to ensure that they are healthy and not liable to pass on any infections to the recipients of the FMT.

FMT has been tested in a small group of patients with cirrhosis in America and was found to be safe, and has also been used more widely in other conditions such as patients with a gut infection called 'clostridium difficile' and patients with inflammatory bowel disease. It is extremely successful in these patients and we would like to see if it can be as successful in liver patients.

Results from this study would support further research into the benefit of using FMT to lessen infection rates in patients with liver cirrhosis who have developed bowel problems.

What groups of patients will the study involve?

We are interested in involving people in this study who have liver cirrhosis of any cause.

For peer review only - http://bmjopen.bmj.com/site/about/guidelines.xhtml

King's College Hospital NHS
NHS Foundation Trust

Why have I been invited?

All patients who are admitted to the Liver wards and/or seen in the Liver out-patient clinics at King's College Hospital NHS Foundation Trust who have liver cirrhosis may be eligible to participate in this study. This also includes patients that are awaiting consideration for or have already been listed for liver transplantation

Do I have to take part?

No, participation is entirely voluntary. It is up to you whether or not you wish to take part. If you decide to take part you will be given this information sheet to keep and asked to sign a consent form. If you decide to take part you are still free to withdraw at any time and without giving a reason. This will not affect the medical care you will receive.

Who is organising and funding this study?

The doctor in charge of this study is Dr Debbie Shawcross. The study is funded by the National Institute of Health Research and is being sponsored by King's College London and Kings College Hospital NHS Foundation Trust.

Who has reviewed the study?

All research in the NHS is looked at by an independent group of people, called a Research Ethics Committee, to protect your interests. This study has been reviewed and approved by the London South East Research Ethics Committee. It has also been approved by the Health Research authority and the Medicines and Healthcare products Regulatory Agency.

What will happen to me if I take part (SUMMARY)?

If you decide to take part you will be randomly assigned to one of two groups. One group (the 'active treatment' group) will receive Faecal Microbiota Transplantation (FMT) whilst the other group (the comparison or 'control group') will receive a 'placebo' treatment. Administration of placebo or FMT will take place only ONCE during this study.

The placebo treatment is a 'dummy' treatment given to participants in the control group of a clinical trial. This method using a 'placebo control' is used to make sure the study results are reliable by checking that any changes measured are actually due to the effect of the active drug and not just because of chance. The placebo in this trial is a solution of saline and glycerol (salty water and a preservative, which are the same ingredients used in the FMT itself, just without the FMT.)

Both groups will then be followed up to see how effective the active treatment is based on various measurements taken. Blood, urine, saliva and stool samples will be obtained for tests at four specific times. Firstly when starting the study at 'baseline' (just before the FMT or placebo is given,) and then repeated at 7, 30 and 90 day intervals after treatment has been administered. Follow-up appointments at the 7, 30 day and 90 days after starting will be arranged to coincide as far as possible with planned routine medical appointments.

For peer review only - http://bmjopen.bmj.com/site/about/guidelines.xhtml

Study procedures	Screening	Baseline (-7 days)	Endoscopy	Day 7 (+/- 5days)	Day 30 (+/- 7 days)	Day 90 (+/- 7 days)
Signed informed consent	X					
Eligibility Criteria	X					
Participant demographics	X					
Medical and surgical histories	**X**			X	X	X
Dietary questionnaire		X		X	X	X
Medication usage		X		X	X	X
Clinical examination		X		X	X	X
Blood sampling		X		X	X	X
Stool sampling		X		X	X	X
Saliva sampling		X		X	X	X
Urine sampling		X		X	X	X
Randomisation		X				
FMT/placebo administration			X			
Adverse events monitoring /Safety			X	X	X	X

As part of the study you will have an endoscopy (a camera test looking into your stomach/small bowel) to give you the FMT or the placebo treatment. This means that you will not have to drink the liquid as it is placed directly into the small bowel where it is needed, avoiding the stomach acid, which could stop it working. Before the endoscopy you will be given a medicine to clear your own gut bacteria called 'Moviprep.' This will give you loose bowel motions, which is a normal reaction to the Moviprep. This is essential to clear your own gut bacteria from the bowel to allow the FMT to work.

We will see you after the endoscopy at days 7, 30 and 90 to take blood, urine, saliva and faecal samples from you. We will also assess your general wellbeing and check that your medicines have not changed. Blood samples will usually be taken at the time of when you are having tests done as part of your routine clinical care, to reduce the number of times you have to have a needle in your arm and, where possible, samples will be taken from tubes already in your blood vessels to minimise discomfort to you.
We will ask you to collect the urine and stool samples in the morning of each study visit and bring this with you when you attend clinic. We will ensure you are given all the necessary collection pots for this. After the last visit at 90 days, you will return to the usual standard of care as you were having for you underlying condition.

More detailed information about the study visits can be found on page 7 of this information sheet.

What are the side effects of taking part?

For peer review only - http://bmjopen.bmj.com/site/about/guidelines.xhtml

If you are assigned to the active treatment group and receive FMT, there are some possible side effects that you should be aware of. Faecal transplants have not been widely used in patients with cirrhosis yet, but have been used in lots of patients with an antibiotic associated diarrhoea called 'clostridium difficile.' Small numbers of patients in these studies reported some abdominal distension/bloating, flatulence, diarrhoea or constipation as side effects of the treatment. Part of this study is to assess how well tolerated the treatment is and whether or not patients with cirrhosis experience similar symptoms.

Side effects of blood sampling can include bruising and it can be uncomfortable. However, every effort will be made to perform blood sampling at the same time as regular blood tests to minimise any discomfort. There should be no side effects of sampling your urine and stool as we will simply ask you to collect the samples when you go to the toilet as normal. Saliva will be collected by asking you to rinse your mouth out with water and to spit into a collection pot.

What are the possible disadvantages and risks of taking part?

We hope that the knowledge we would gain from this study will improve our understanding of the way in which FMT works, and the role of the gut and immune system in liver cirrhosis. Patients who received FMT in a small study in America did well and had fewer hospital admissions than those in the 'standard of care' group. FMT appears to be safe in the considerable numbers of patients who have received it for other reasons. There are no disadvantages or risks involved in taking part with regard to the samples being obtained, and the amount of blood taken will not be harmful to you. The placebo treatment is not harmful, but is not expected to have any benefit on your clinical condition as it contains no active drug or therapy. All patients, regardless of whether they receive placebo or FMT have the same rigorous follow up and support from the trials team.

You will be closely monitored after the treatment is given and will be seen at regular intervals afterwards to see if you have experienced any side effects. We do not anticipate any serious problems to occur after the FMT or placebo treatment, but you will be given contact numbers should you run into any problems outside of the trial visits.

Apart from the side effects of FMT, all patients (those on placebo and active treatment) will need to take bowel preparation, which can cause dehydration. The endoscopy itself also has a small risk of bleeding, perforation (this is very rare and is thought to occur in around only 1 in 2000 tests) and damage to teeth and dental work. There is also a risk of aspiration of the FMT or placebo into the lungs, therefore you will be monitored closely after the procedure to monitor for this.

What are the possible benefits of taking part?

You will be taking part in a new and innovative trial to assess the effect of FMT on cirrhosis. There are few treatments available to patients with cirrhosis, aside from liver transplantation. Unfortunately due to the scarcity of donor organs, even those listed for transplant can wait months or even years for a new liver. Others are too unwell or frail to be listed for transplant. We hope that looking at FMT in more detail may provide a much needed treatment for patients with cirrhosis who are not suitable for transplant or to help those awaiting a new liver. This study is designed to look at the safety and tolerability of the treatment to see if a larger study would show an improvement in wellbeing for these patients.

For peer review only - http://bmjopen.bmj.com/site/about/guidelines.xhtml

King's College Hospital NHS
NHS Foundation Trust

What if I lose the ability to provide consent after enrolling to the study ('loss of capacity)?

In the event that you lose the ability to provide ongoing consent during the research due to your illness, having been able to provide consent at the start of the study, we will approach an appropriate relative or friend on your behalf to ask them if they think that you would have wanted to continue participation in this study. This person is termed a 'Consultee' and they are asked to set aside their own views and provide advice on you continuing to participate in the research, taking into consideration your wishes and interests.

Any advance decisions you may have made, and that they are aware of, should take precedence. If the Consultee decides that you would have no objection to continue taking part they will be asked to read and sign a consultee declaration form. They will then be given a copy to keep. If they decide that you would not wish to continue taking part we will withdraw you from the study; this will not, in any way, affect the standard of care you receive. If the Consultee we approach does not feel able to take on this role they may want to identify someone else to do it. They will be asked to take time to decide whether or not they feel that you would wish to continue taking part. They will be encouraged to ask the research team if there is anything that is not clear or if they would like more information. The information they will be provided with is the same as that provided to you.

You may wish to identify a suitable Consultee at this stage given the above, so that if you do lose the ability to provide consent after you are enrolled (should you decide to participate in the study) we can approach a person of your choice.

What if something goes wrong?

If you have a concern about any aspect of this study, you should ask to speak to your study doctor who will do their best to answer your questions (contact details on page 8) If you remain unhappy and wish to complain formally, you can do this through the NHS Complaints procedure by contacting your local Patient Advice Liaison Service (PALS) office. Details of your local office can be obtained by asking your study doctor, GP, telephoning your local hospital or looking on the NHS choices website. http://www.nhs.uk/pages/home.aspx

Every care will be taken in the course of this study. However in the unlikely event that you are injured by taking part, compensation may be available.

In the event that something does go wrong and you are harmed during the research and this is due to someone's negligence then you may have grounds for a legal action for compensation against King's College Hospital NHS Foundation Trust & King's College London, but you may have to pay your legal costs.

Regardless of this, if you wish to complain, or have any concerns about any aspect of the way you have been approached or treated by members of staff or about any side effects (adverse events) you may have experienced due to your participation in the study the normal National Health Service

For peer review only - http://bmjopen.bmj.com/site/about/guidelines.xhtml

complaints mechanisms are available to you. Please ask your study doctor if you would like more information on this.

Will my taking part in this study be kept confidential?

Yes. Once you have consented to take part in the study, you will be given a unique study number, which will be used to identify any samples and information collected during the research.

Some information regarding you and your condition will be recorded as part of this study. We will not record any personal identifiable information (name, date of birth or contact details) as part of the research records. All information will be stored anonymously in a password-protected database, or in a file in a secure research office. Only your treating doctors and the research team will have access to this information.

We will inform your General Practitioner (GP) of your participation in the study - this is routine for this type of research and helps them to care for you during the trial. We advise that you inform your private medical insurance provider (if you hold private medical insurance) of your participation in this study - this again is routine procedure for this type of research.

What will happen to the results of the research study?

When we have results for an adequate number of patients after the study is completed we plan to publish them in an international journal so that the information can benefit as many people as possible. We can provide you with a brief summary of the results of the study when available should you desire this.

What will happen to any samples that I give?

Every effort will be made to ensure blood samples for the study are taken at the same time as samples are taken as part of your routine clinical care – so that there will be no additional discomfort to you as a result of being involved in this study. The amount of blood taken per time point for the study is approximately 50mls or 10 teaspoonfuls. This is a small amount for the body (which contains about 5L of blood, or 5000mL) and will not adversely affect you in any way.

Samples of blood and other fluids obtained may be used immediately or saved for human or bacterial DNA or immune system analysis in the future, in the Liver Biobank. Some of the actual tests on the samples that are obtained as a result of you taking part in the study will be performed on site at King's College Hospital NHS Foundation Trust, whilst some samples will be transferred to other collaborator laboratories in the UK and France. At all times any samples that are transferred and stored will be coded anonymously regardless of which laboratory they are sent to.

Contact details:

Thank you for taking the time to read this information.

If there is any other information you would like, please do not hesitate to contact us on the numbers below. Out of hours or if a response on the above contact number is unavailable, it is possible to contact the on-call Hepatology Registrar – via KCH Switchboard – who will pass any messages on to the senior medical staff involved.

For peer review only - http://bmjopen.bmj.com/site/about/guidelines.xhtml

King's College Hospital NHS
NHS Foundation Trust

Principal investigator: Dr Debbie Shawcross Tel: 02032992504
Clinical research fellow: Dr C Woodhouse Tel: 02032992504
Research nurse: Ane Zamalloa Tel: 0203299 7623

For peer review only

For peer review only - http://bmjopen.bmj.com/site/about/guidelines.xhtml

King's College Hospital NHS
NHS Foundation Trust

What will happen to me if I take part (DETAILED EXPLANATION)?

You will be assessed for your eligibility to be involved in this study, and if you are happy to proceed will sign the consent form with a member of the research team. You will then be randomly allocated to one of two treatment groups:

1) the 'active treatment' group: participants in this group will receive the treatment being tested (FMT), or
2) the comparison or 'control group'; participants in this group will receive a 'placebo' treatment, which is a 'dummy' treatment and is indistinguishable from the actual treatment given to participants in the 'active treatment' group.

Before starting treatment with either of the above, you will undergo baseline assessments, which involve the following (those marked with * are being done as part of the study):

- Medical history including current medication usage.
- Physical examination including blood pressure, pulse rate, height, weight.
- Dietary questionnaire* and quality of life questionnaire*
- Blood sampling: will include tests of blood counts, kidney and liver function performed as part of your routine clinical care as well as checks for various viruses that can affect the liver.
- Blood sampling*: to measure endotoxin levels, immune function including DNA analysis and metabolic profiling.
- Urine sampling*: we will ask you to provide a urine sample (approx. 20mls) to measure metabolic profiling. If you are a woman of child-bearing age, we will also do a pregnancy test.
- Stool sampling*: we will ask you to provide stool samples to measure what types of bacteria are found, and in what patterns and quantities.
- Salivary sampling: *: we will ask you to provide saliva samples from your mouth to measure what types of bacteria are found, and in what patterns and quantities.
- If you have fluid in your abdomen called "ascites" we would like to take a sample of the fluid to check for infection. This involves passing a small needle into your abdomen under aseptic conditions. This test is voluntary and you do not have to take part, but it would help us to check for infection in the fluid and would mean we could treat any infection early, should one be present*.

After the baseline assessments have been completed, you will come up to the endoscopy unit to have the FMT or placebo treatment given. The procedure will be explained to you and the risks and benefits discussed. Endoscopy is a safe test, but like all procedures has some risks. The main risks to be aware of are a small risk of bleeding and the risk of perforation (1 in 9000.) There is also a risk of damaging teeth or dental work and a small risk of 'aspiration' which is where fluid from the stomach goes into the lungs. This is why we ask you not to eat for six hours prior to the test, to make sure the stomach is empty. Some people also develop a sore throat after the procedure. This should get better on its own.

You will also be given a medicine prior to the test called 'Moviprep' to help clear your bowels of their usual bacteria prior to the treatment with FMT or placebo. This is a safe medicine, but will cause you to have loose stools. This is essential to its function, but it is important you drink

For peer review only

King's College Hospital NHS
NHS Foundation Trust

plenty of fluid to prevent dehydration. Side effects can include dehydration, nausea and vomiting.

You will take one sachet of Moviprep mixed with a litre of water at 19.00 the night before the test. You can then have a light meal. On the morning of the endoscopy you will drink another sachet of Moviprep mixed with one litre of water at around 7.00am. You can take your usual medications as normal. There is a lot of fluid to drink, so you may find it easier if it is chilled or mixed with a squash (not blackcurrant) to make it easier to drink.

You will undergo similar assessments to the baseline visit at 7, 30 and 90 day intervals as you did just before starting treatment. You will in addition undergo the following as part of the study:

- Study treatment adverse events monitoring*: this to check whether you may have experienced any side effects that may be due to the study medication.

Your last visit will be at 90 days from the start of the trial. Samples will be collected and you will be reviewed by the team to check for any side effects. You will have the opportunity to ask questions to the team at all visits throughout the duration of the study and if you have any concerns outside of these visits you will be told who to contact.

Thank you for taking the time to read this information. If there is any other information you would like, please do not hesitate to contact us (020 3299 9000 ext. 32504).

Dr C Woodhouse, Dr V. C. Patel, Professor A Sanchez-Fueyo, Dr D. Shawcross
Institute of Liver Studies, Kings College Hospital

For peer review only

For peer review only - http://bmjopen.bmj.com/site/about/guidelines.xhtml

Faecal Microbiota Transplant (FMT) for recurrent or refractory *Clostridium difficile* Infection (CDI)

Donor screening questionnaire – Part 1

Name:

DOB:

Hospital number:

NHS number:

Contact details (preferably mobile):

Date of assessment:

Name / position of assessor:

Donor type	☐ Named donor<br>☐ Anonymous donor
If Named donor, name and hospital number of recipient	
If Named donor, relationship to recipient	
If Named donor, does the recipient normally live in the same dwelling?	☐ Yes ☐ No
Age	Exclude if <18 or >60
Gender	☐ Male ☐ Female
Ethnicity	☐ White - White British<br>☐ White - White Irish<br>☐ White - Other<br>☐ Mixed race – White and Black Caribbean<br>☐ Mixed race – White and Black African<br>☐ Mixed race – White and Asian<br>☐ Mixed race – Other<br>☐ Asian or Asian British – Indian<br>☐ Asian or Asian British – Bangladeshi<br>☐ Asian or Asian British – Pakistani<br>☐ Asian or Asian British – Other<br>☐ Black or Black British – Caribbean<br>☐ Black or Black British – African<br>☐ Black or Black British – Other<br>☐ Chinese<br>☐ Other
Height cm	

For peer review only

Weight kg	
BMI	Exclude if BMI>25
Has your weight changed by more than 5lb / 2kg in the past 6 months?	☐ Yes ☐ No<br><br>Detail:
Describe your diet (as many as apply):	☐ Omnivore<br>☐ Vegetarian<br>☐ Vegan<br>☐ Kosher<br>☐ Halal<br>☐ Raw food only<br>☐ Pescatarian<br>☐ No red meat<br>☐ Low carbohydrate<br>☐ Lactose free<br>☐ Gluten free<br>☐ Other
How many portions of fruit and vegetables do you consume per day?	☐ one or less<br>☐ two to three<br>☐ three to four<br>☐ five to six<br>☐ seven or more
How many servings of cow, sheep or goats milk do you consume per day?	☐ one or less<br>☐ two to three<br>☐ three to four<br>☐ five to six<br>☐ seven or more
Alcohol – units/week	
Smoking/day	
Normal bowel habit – average Bristol Stool Consistency	☐ 1<br>☐ 2<br>☐ 3<br>☐ 4<br>☐ 5<br>☐ 6<br>☐ 7<br><br>Exclude if 6 or 7
Normal bowel habit – average frequency	☐ >2/day<br>☐ once to twice daily<br>☐ once / 2 days<br>☐ <once / 2 days<br><br>Exclude if active diarrhoea (>3 UBM/day for at least 2 consecutive days)

For peer review only

During the past 7 days how many days were you physically active for a cumulative total of >60 mins/day?	
Have you ever been rejected as a blood donor/told not to donate?<br>If yes, why?	☐ Yes ☐ No<br>Exclude if YES
What is your country of birth?	
Have you ever resided in another country (other than UK) for >5 years?<br>If so which countries and when?	☐ Yes ☐ No
Do you currently have a profession that is associated with an increased risk of blood-borne transmissible diseases (e.g. daily contact with patients/inmates)?<br>If yes, what profession?	☐ Yes ☐ No<br>Exclude if health/social care worker with direct patient contact
Have you ever had a needle-stick or injury from a blood contaminated object from someone else? If yes, when and how was this follow up?	☐ Yes ☐ No
Have you ever injected yourself or been injected with illegal or non-prescribed drugs including body building drugs or cosmetics (even if this was only once or a long time ago?)	☐ Yes ☐ No<br>Exclude if YES
Have you ever had a tattoo?<br>If yes, when and in which country was it performed?	☐ Yes ☐ No<br>Exclude if within past 4 months
Have you ever had a piercing?<br>If yes, when and in which country was it performed?	☐ Yes ☐ No<br>Exclude if within past 4 months
Have you ever had acupuncture?<br>If yes, when and in which country was it performed?	☐ Yes ☐ No<br>Exclude if within past 4 months
Have you ever had an operation or undergone clinical treatment in a hospital with poor hygienic conditions?<br>If yes, when and in which country was it performed?	☐ Yes ☐ No
Have you ever had a rare infectious disease (e.g. tuberculosis, trypanosomiasis)?<br>If yes, when and which disease?	☐ Yes ☐ No
Have you ever been vaccinated against Hepatitis A or B?<br>If yes, which?	☐ Yes ☐ No

For peer review only

Have you received a live vaccine in the past 6 months?	☐ Yes ☐ No
In the last 12 months have you had sex with anyone who is HIV positive?	☐ Yes ☐ No<br>Exclude if Yes
In the last 12 months have you had sex with anyone with hepatitis B, hepatitis C or HTLV?	☐ Yes ☐ No<br>Exclude if Yes
In the last 12 months have you had sex with anyone who has ever been given money or drugs for sex?	☐ Yes ☐ No<br>Exclude if Yes
In the last 12 months have you had sex with anyone who has ever injected drugs?	☐ Yes ☐ No<br>Exclude if Yes
In the last 12 months have you had sex with anyone who may ever have had sex in parts of the world where HIV/AIDS is very common (this includes most countries in Africa)?	☐ Yes ☐ No<br>Exclude if Yes
**Male donors ONLY:**<br>In the last 12 months have you ever had oral or anal sex with a man, with or without a condom?	☐ Yes ☐ No<br>Exclude if Yes
**Female donors ONLY:**<br>In the last 12 months have you had sex with a man who has ever had oral or anal sex with another man, with or without a condom?	☐ Yes ☐ No<br>Exclude if Yes
Have you ever been treated for an intestinal infection?<br>If yes, which one and when?	☐ Yes ☐ No
Do you have any intestinal conditions:<br>Barrett's Oesophagus<br>Coeliac disease<br>Diverticular disease<br>Dyspepsia<br>Gastric ulcer<br>Gastro-oesophageal reflux disease<br>Hepatitis<br>H. pylori infection<br>Crohns disease<br>Ulcerative colitis<br>Other inflammatory bowel diseases<br>Irritable Bowel Syndrome<br>Lactose intolerance<br>Liver disease<br>Pancreatitis<br>Gastrointestinal malignancy or polyps	 ☐ Yes ☐ No<br>☐ Yes ☐ No<br>☐ Yes ☐ No<br>☐ Yes ☐ No<br>☐ Yes ☐ No<br>☐ Yes ☐ No<br>☐ Yes ☐ No<br>☐ Yes ☐ No<br>☐ Yes ☐ No<br>☐ Yes ☐ No<br>☐ Yes ☐ No<br>☐ Yes ☐ No<br>☐ Yes ☐ No<br>☐ Yes ☐ No<br>☐ Yes ☐ No<br>☐ Yes ☐ No<br><br>Exclude if Inflammatory Bowel Disease, Irritable Bowel Syndrome, GI malignancy, Hepatitis A, B, C or E

For peer review only

Have you used any antibiotics in the last 3 months?	☐ Yes ☐ No<br><br>Exclude if Yes
Have you had a fever in the last 2 weeks?	☐ Yes ☐ No<br><br>Exclude if Yes
Have you ever been incarcerated in prison?	☐ Yes ☐ No<br><br>Exclude if in past 4 months
Have you ever been immunosuppressed (e.g. during treatment for cancer, or for a solid organ transplant)?<br>If yes, when and why?	☐ Yes ☐ No
Have you ever had major gastrointestinal surgery?<br>If yes, when and why?	☐ Yes ☐ No<br><br>Relative exclusion criteria
Have you ever suffered from metabolic syndrome?	☐ Yes ☐ No<br><br>Relative exclusion criteria
Have you ever suffered from any autoimmune condition (e.g. rheumatoid), asthma or eczema?<br>If yes, which, and do you take any treatment?	☐ Yes ☐ No<br><br>Relative exclusion criteria
Have you ever had any chronic pain or fatigue syndromes?<br>If yes, which?	☐ Yes ☐ No<br><br>Relative exclusion criteria
Have you any history of CJD or other prion disease in your family? If yes, please specify<br>Patients should be considered to be at risk from genetic forms of CJD if they have or have had<br>1. Genetic testing, which has indicated they are at significant risk of developing CJD or other prion disease<br>2. A blood relative known to have a genetic mutation indicative of genetic CJD or other prion disease<br>3. Two or more blood relatives affected by CJD or other prion disease	☐ Yes ☐ No
Have you ever received growth hormone or gonadotrophic treatment? If yes, please specify;<br>i) Whether the hormone was derived from human pituitary glands<br>ii) The year of the treatment<br>iii) Whether the treatment was received in the UK or in another country	☐ Yes ☐ No

Recipients of hormone derived from human pituitary glands eg growth hormone or gonadtotrophin have been identified as potentially at risk of CJD. In the UK, the use of human growth hormone was stopped in 1985 but human-derived products may have been continued to be used in other countries. In the UK, the use of human-derived gonadotrophin was discontinued in 1973 but may have been continued in other countries after this time.	
Have you ever had surgery on your brain or spinal cord?<br>People who underwent intradural neurosurgical or spinal procedures before August 1992 may have received a graft of human-derived dura mater and should be treated as at increased risk, unless evidence can be provided that human-derived dura mater was not used. Patients who received a graft of human-derived dura mater between 1980 and August 1992 are at increased risk of both sporadic CJD and vCJD.	☐ Yes ☐ No
Are you normally resident in the UK?<br>If No state country of usual residence	☐ Yes ☐ No
Which countries have you visited in the last 12 months and what was the duration of stay?	
In the past 12 months have you been admitted to a hospital in a country other than the UK?<br><br>If yes state when and which countries	☐ Yes ☐ No
In the past 12 months have you been admitted to another hospital in London or Manchester?<br><br>If yes state when and which hospitals	☐ Yes ☐ No
List all current medications:	

For peer review only

	Exclude if any regular prescribed drugs (except OCP)

Is patient eligible to donate?	☐ Yes ☐ No
If No document reasons:	
If Yes proceed to screening tests<br>Ensure consent for testing documented	
Sign and date (health care practitioner) to indicate patient has provided consent	

Obtain serology for:	Requested?
HIV antigen/antibody	☐ Yes
HTLV-1/HTLV-II antibodies	☐ Yes
Hepatitis A IgG (add on IgM if positive)	☐ Yes
Hepatitis B surface antigen	☐ Yes
Hepatitis C IgG	☐ Yes
Syphilis serology (T. pallidum antibodies)	☐ Yes
CMV IgG (add on IgM if positive)	☐ Yes
EBV Serology	☐ Yes
Strongyloides serology	☐ Yes

Provide stool sample collection pots and request forms x3 for patient to take home. Instruct patient to provide three separate samples over 2-3 days, ensuring the pot is filled halfway in order to complete all testing.

Stool testing for:	Requested?
Bacterial culture (Campylobacter, Salmonella, Shigella, E. coli O:157) x3	☐ Yes
Ova, Cysts and Parasites x3	☐ Yes
C. difficile	☐ Yes
Resistant Gram negative organism screen	☐ Yes
MRSA screen	☐ Yes
Helicobacter pylori stool antigen	☐ Yes
E. histolytica PCR (not orderable on PCR)	

Arrange follow up visit for a minimum of 2-3 weeks time

Follow-up visit

Serology results:	Result
HIV antigen/antibody	☐ Detected ☐ Not detected

For peer review only

HTLV-1/HTLV-II antibodies	☐ Detected ☐ Not detected
Hepatitis A IgG (add on IgM if positive)	☐ Detected ☐ Not detected
Hepatitis B surface antigen	☐ Detected ☐ Not detected
Hepatitis C IgG	☐ Detected ☐ Not detected
Syphilis serology (T. pallidum antibodies)	☐ Detected ☐ Not detected
CMV IgG (add on IgM if positive)	☐ Detected ☐ Not detected
EBV Serology	☐ Detected ☐ Not detected
Strongyloides serology	☐ Detected ☐ Not detected

Stool results:	Result
Bacterial culture (Campylobacter, Salmonella, Shigella, E. coli O:157) x3	☐ Detected ☐ Not detected
Ova, Cysts and Parasites x3	☐ Detected ☐ Not detected
C. difficile	☐ Detected ☐ Not detected
Resistant Gram negative organism screen	☐ Detected ☐ Not detected
MRSA screen	☐ Detected ☐ Not detected
Helicobacter pylori stool antigen	☐ Detected ☐ Not detected
E. histolytica PCR (not orderable on PCR)	☐ Detected ☐ Not detected

Is all testing complete?	☐ Yes ☐ No
Accept as donor?	☐ Yes ☐ No
Any referrals required	☐ Yes ☐ No
Details of referrals made:	

Provide donor with stool collection kit x2 (contains collection instructions and copy of patient screening questionnaire 2). Explain requirement for stool to be fully processed within 6 hours of production.

The donor may provide multiple donations, but screening questionnaire 2 **MUST** be completed with **EACH** donation

If yes to any question, must undergo repeat full screening (however samples may be banked and quarantined for release after repeat testing).

If No to all questions samples may be released immediately and donor may continue to provide unlimited donations for 3 months after which time repeated screening will be required.

For peer review only

SPIRIT

STANDARD PROTOCOL ITEMS: RECOMMENDATIONS FOR INTERVENTIONAL TRIALS

SPIRIT 2013 Checklist: Recommended items to address in a clinical trial protocol and related documents*

Section/item	Item No	Description
**Administrative information**		
Title	1	Descriptive title identifying the study design, population, interventions, and, if applicable, trial acronym (Page 1)
Trial registration	2a	Trial identifier and registry name. If not yet registered, name of intended registry (Page 1)
	2b	All items from the World Health Organization Trial Registration Data Set
Protocol version	3	Date and version identifier (Page 1)
Funding	4	Sources and types of financial, material, and other support (Pages 9, 47)
Roles and responsibilities	5a	Names, affiliations, and roles of protocol contributors (Pages 2-4)
	5b	Name and contact information for the trial sponsor (Pages 3-4)
	5c	Role of study sponsor and funders, if any, in study design; collection, management, analysis, and interpretation of data; writing of the report; and the decision to submit the report for publication, including whether they will have ultimate authority over any of these activities N/A
	5d	Composition, roles, and responsibilities of the coordinating centre, steering committee, endpoint adjudication committee, data management team, and other individuals or groups overseeing the trial, if applicable (see Item 21a for data monitoring committee) (Page 47)
**Introduction**		
Background and rationale	6a	Description of research question and justification for undertaking the trial, including summary of relevant studies (published and unpublished) examining benefits and harms for each intervention (Pages 17-20)
	6b	Explanation for choice of comparators (Pages 17-20)
Objectives	7	Specific objectives or hypotheses (Pages 21-22)

For peer review only

Trial design	8	Description of trial design including type of trial (eg, parallel group, crossover, factorial, single group), allocation ratio, and framework (eg, superiority, equivalence, noninferiority, exploratory) (Pages 23-25)

Methods: Participants, interventions, and outcomes

Study setting	9	Description of study settings (eg, community clinic, academic hospital) and list of countries where data will be collected. Reference to where list of study sites can be obtained (Page 23)
Eligibility criteria	10	Inclusion and exclusion criteria for participants. If applicable, eligibility criteria for study centres and individuals who will perform the interventions (eg, surgeons, psychotherapists) (Page 12, Page 29)
Interventions	11a	Interventions for each group with sufficient detail to allow replication, including how and when they will be administered (Pages 32-36)
	11b	Criteria for discontinuing or modifying allocated interventions for a given trial participant (eg, drug dose change in response to harms, participant request, or improving/worsening disease) (Pages 15-16, 36-39)
	11c	Strategies to improve adherence to intervention protocols, and any procedures for monitoring adherence (eg, drug tablet return, laboratory tests) (N/A, single treatment administered by clinical trials team)
	11d	Relevant concomitant care and interventions that are permitted or prohibited during the trial (Pages 29-31)
Outcomes	12	Primary, secondary, and other outcomes, including the specific measurement variable (eg, systolic blood pressure), analysis metric (eg, change from baseline, final value, time to event), method of aggregation (eg, median, proportion), and time point for each outcome. Explanation of the clinical relevance of chosen efficacy and harm outcomes is strongly recommended (Pages 10-11, 21-22)
Participant timeline	13	Time schedule of enrolment, interventions (including any run-ins and washouts), assessments, and visits for participants. A schematic diagram is highly recommended (see Figure) (Pages 24-28)
Sample size	14	Estimated number of participants needed to achieve study objectives and how it was determined, including clinical and statistical assumptions supporting any sample size calculations (Pages 45-46)
Recruitment	15	Strategies for achieving adequate participant enrolment to reach target sample size (Pages 23, 25)

Methods: Assignment of interventions (for controlled trials)

Allocation:

Sequence generation	16a	Method of generating the allocation sequence (eg, computer-generated random numbers), and list of any factors for stratification. To reduce predictability of a random sequence, details of any planned restriction (eg, blocking) should be provided in a separate document that is unavailable to those who enrol participants or assign interventions (Page 31)
Allocation concealment mechanism	16b	Mechanism of implementing the allocation sequence (eg, central telephone; sequentially numbered, opaque, sealed envelopes), describing any steps to conceal the sequence until interventions are assigned (Page 31, single blinded so only the patient is blinded)
Implementation	16c	Who will generate the allocation sequence, who will enrol participants, and who will assign participants to interventions (Trials Nurse, Chief Investigator or Clinical Research Fellow using randomisation software)
Blinding (masking)	17a	Who will be blinded after assignment to interventions (eg, trial participants, care providers, outcome assessors, data analysts), and how (Patient is blinded to treatment allocation as is trial statistician, database constructed so as not to reveal treatment allocation)
	17b	If blinded, circumstances under which unblinding is permissible, and procedure for revealing a participant's allocated intervention during the trial (N/A)

Methods: Data collection, management, and analysis

Data collection methods	18a	Plans for assessment and collection of outcome, baseline, and other trial data, including any related processes to promote data quality (eg, duplicate measurements, training of assessors) and a description of study instruments (eg, questionnaires, laboratory tests) along with their reliability and validity, if known. Reference to where data collection forms can be found, if not in the protocol
	18b	Plans to promote participant retention and complete follow-up, including list of any outcome data to be collected for participants who discontinue or deviate from intervention protocols
Data management	19	Plans for data entry, coding, security, and storage, including any related processes to promote data quality (eg, double data entry; range checks for data values). Reference to where details of data management procedures can be found, if not in the protocol (Page 43)
Statistical methods	20a	Statistical methods for analysing primary and secondary outcomes. Reference to where other details of the statistical analysis plan can be found, if not in the protocol (Pages 45-46)
	20b	Methods for any additional analyses (eg, subgroup and adjusted analyses) (To be confirmed)

	20c	Definition of analysis population relating to protocol non-adherence (eg, as randomised analysis), and any statistical methods to handle missing data (eg, multiple imputation) (Pages 45-46)
**Methods: Monitoring**		
Data monitoring	21a	Composition of data monitoring committee (DMC); summary of its role and reporting structure; statement of whether it is independent from the sponsor and competing interests; and reference to where further details about its charter can be found, if not in the protocol. Alternatively, an explanation of why a DMC is not needed (Page 47)
	21b	Description of any interim analyses and stopping guidelines, including who will have access to these interim results and make the final decision to terminate the trial (Pages 39-40, 47)
Harms	22	Plans for collecting, assessing, reporting, and managing solicited and spontaneously reported adverse events and other unintended effects of trial interventions or trial conduct (Pages 36-38)
Auditing	23	Frequency and procedures for auditing trial conduct, if any, and whether the process will be independent from investigators and the sponsor (Pages 14, 43-44)
**Ethics and dissemination**		
Research ethics approval	24	Plans for seeking research ethics committee/institutional review board (REC/IRB) approval (Page 14) Research Ethics approval was given by the London South East Research Ethics committee (ref 17/LO/2081)
Protocol amendments	25	Plans for communicating important protocol modifications (eg, changes to eligibility criteria, outcomes, analyses) to relevant parties (eg, investigators, REC/IRBs, trial participants, trial registries, journals, regulators) Direct communication with REC and involvement of DMC and TSC as required
Consent or assent	26a	Who will obtain informed consent or assent from potential trial participants or authorised surrogates, and how (see Item 32) (Pages 14-16)
	26b	Additional consent provisions for collection and use of participant data and biological specimens in ancillary studies, if applicable (N/A)
Confidentiality	27	How personal information about potential and enrolled participants will be collected, shared, and maintained in order to protect confidentiality before, during, and after the trial (Pages 15, 23, 31, 43, 44)
Declaration of interests	28	Financial and other competing interests for principal investigators for the overall trial and each study site N/A

For peer review only

Access to data	29	Statement of who will have access to the final trial dataset, and disclosure of contractual agreements that limit such access for investigators (Page 43)
Ancillary and post-trial care	30	Provisions, if any, for ancillary and post-trial care, and for compensation to those who suffer harm from trial participation (Information in PIS)
Dissemination policy	31a	Plans for investigators and sponsor to communicate trial results to participants, healthcare professionals, the public, and other relevant groups (eg, via publication, reporting in results databases, or other data sharing arrangements), including any publication restrictions (Page 47)
	31b	Authorship eligibility guidelines and any intended use of professional writers N/A
	31c	Plans, if any, for granting public access to the full protocol, participant-level dataset, and statistical code (Page 47)
**Appendices**		
Informed consent materials	32	Model consent form and other related documentation given to participants and authorised surrogates
Biological specimens	33	Plans for collection, laboratory evaluation, and storage of biological specimens for genetic or molecular analysis in the current trial and for future use in ancillary studies, if applicable

*It is strongly recommended that this checklist be read in conjunction with the SPIRIT 2013 Explanation & Elaboration for important clarification on the items. Amendments to the protocol should be tracked and dated. The SPIRIT checklist is copyrighted by the SPIRIT Group under the Creative Commons "Attribution-NonCommercial-NoDerivs 3.0 Unported" license.

For peer review only

BMJ Open

PROFIT: A PROspective, randomised placebo controlled feasibility trial of Faecal mIcrobiota Transplantation in cirrhosis: study protocol for a single-blinded trial

Journal:	*BMJ Open*
Manuscript ID	bmjopen-2018-023518.R2
Article Type:	Protocol
Date Submitted by the Author:	26-Nov-2018

Complete List of Authors:	Woodhouse, Charlotte; King's College London, Hepatology<br>Patel, Vishal; Kings College London, Hepatology<br>Goldenberg, Simon; Guy's & St. Thomas' NHS Foundation Trust and King's College,<br>Sanchez Fueyo, Alberto; Kings College London, Hepatology<br>China, Louise; UCL, Division of Medicine<br>O'Brien, Alastair; UCL, Division of Medicine<br>Flach, Clare; 1. School of Population Health & Environmental Sciences, Faculty of Life Sciences & Medicine, King's College London<br>Douiri, Abdel; 1. School of Population Health & Environmental Sciences, Faculty of Life Sciences & Medicine, King's College London<br>Shawcross, Debbie ; Kings College London, Liver Sciences, 1st Floor JBC, School of Immunology & Microbial Sciences Faculty of Life Sciences & Medicine, King’s College London, Denmark Hill Campus, London, SE5 9RS

**Primary Subject Heading**:	Gastroenterology and hepatology
Secondary Subject Heading:	Pharmacology and therapeutics
Keywords:	cirrhosis, faecal microbiota transplantation, gut microbiota, feasibility

SCHOLARONE™
Manuscripts

PROFIT: A PROspective, randomised placebo controlled feasibility trial of Faecal mIcrobiota Transplantation in cirrhosis: study protocol for a single-blinded trial

C. Woodhouse[1], V. Patel[1], S. Goldenberg[2], A. Sanchez-Fueyo[1], L. China[3], A. O'Brien[3], C. Flach[4], A. Douiri[4], D. Shawcross[1]

1. **Liver Sciences, 1st Floor James Black Centre, School of Immunology & Microbial Sciences, Faculty of Life Sciences & Medicine, King's College London, Denmark Hill Campus, London, SE5 9RS**
2. **Directorate of Infection, Guy's and St Thomas' NHS Foundation Trust, London**
3. **Division of Medicine, University College London (UCL), London**
4. **School of Population Health & Environmental Sciences, Faculty of Life Sciences & Medicine, King's College London**

Corresponding author: Dr Charlotte Woodhouse, Liver Sciences, 1st Floor James Black Centre, School of Immunology & Microbial Sciences, Faculty of Life Sciences & Medicine, King's College London, Denmark Hill Campus SE5 9RS charlottewoodhouse@nhs.net Tel 02032993713 Fax: 020232993899

Abstract

Introduction

Patients with advanced cirrhosis have enteric bacterial dysbiosis and translocation of bacteria and their products across the gut epithelial barrier. This culminates in systemic inflammation and endotoxemia, inducing innate immune dysfunction which predisposes to infection, and development of complications such as bleeding, sepsis and hepatic encephalopathy (HE). This feasibility study aims to assess the safety of administering faecal microbiota transplant to patients with cirrhosis and explore the effect of the intervention on their prognosis by achieving restoration of a healthy gut microbiome.

Methods and Analysis

PROFIT is a single-centre, randomized, single-blinded, placebo-controlled study evaluating Faecal Microbiota Transplantation (FMT) against placebo. Patients with advanced but stable cirrhosis with a MELD score between 10 and 16 will be recruited. Twenty-four patients will be randomized to FMT plus standard of care (as per our institutional practice) and eight patients to placebo in a ratio of 3:1. Patients will be evaluated at baseline before the study intervention is administered and at 7, 30 and 90 days post intervention to assess safety and adverse events. FMT/placebo will be administered into the jejunum within 7 days of baseline.

The primary outcome measure will be safety and feasibility as assessed by recruitment rates, tolerability and safety of FMT treatment. Results will be disseminated via peer

For peer review only

reviewed journals and international conferences. The recruitment of the first patient occurred on 23/05/2018.

Ethics and dissemination

Research Ethics approval was given by the London South East Research Ethics committee (ref 17/LO/2081). The study has been registered with *ClinicalTrials.gov*– reference number: NCT02862249. The trial is registered with the European Medicines Agency (EudraCT 2017-003629-13) and has been adopted by the NIHR (IRAS 197237). This manuscript refers to the version 2.0 of the protocol; pre-results.

Strengths and limitations of this study

- This study is powered to assess feasibility and safety of administering FMT to patients with cirrhosis, however it is not statistically powered to assess for clinically relevant outcomes.
- This is the first study examining the effect of FMT delivered directly into the small bowel in patients with advanced cirrhosis. This trial does not involve antibiotic pre-treatment in the FMT group, as has been undertaken in the USA in patients with HE.
- PROFIT will assess instillation of FMT/placebo directly into the small bowel, as opposed to the colon-, directly targetting small bowel bacterial overgrowth that is observed in cirrhosis
- A limitation of the study is its single-blinded design, which was necessary as the FMT and placebo (saline with glycerol) are not matched.

Introduction

Patients with advanced cirrhosis have enteric dysbiosis with small bowel bacterial overgrowth and translocation of bacteria and their products across the gut epithelial barrier (1). This culminates in systemic inflammation and endotoxaemia, inducing innate immune dysfunction which predisposes to infection (2), and development of complications such as bleeding, sepsis and hepatic encephalopathy (3). It also plays a key role in the natural history of cirrhosis by influencing the rate of progression to advanced liver disease and terminal liver failure (4).

Utilising quantitative metagenomics our group has found 75,245 genes differentially expressed between patients with cirrhosis and healthy individuals. Over 50% of these bacterial species are of buccal origin suggesting an invasion of the gut from the mouth in cirrhosis (5). Patients with cirrhosis also have salivary dysbiosis associated with impaired salivary defenses and systemic inflammation. Salivary dysbiosis has been shown to be greater in patients with cirrhosis who developed complications necessitating hospitalisation within 90 days. Modulating the gut microbiota in patients with cirrhosis with the non-absorbable antibiotic rifaximin has been associated with improved cognitive performance and reduction in endotoxaemia in patients with cirrhosis (6, 7). Moreover, we have recently performed a multi-centre retrospective study including 170 patients in which rifaximin-α therapy given for 90 days significantly (i) reduced hospital re-admission rates after 3 months treatment, impacting significantly on the NHS resource burden and (ii) reduced overall liver disease severity (as measured by the Child Pugh and Model for End Stage Liver Disease scores) raising

For peer review only

the possibility that modulation of gut microbiota may significantly modify the natural history of chronic liver failure (8).

These data constitute in our view "proof of principle" that modifying the gut microbiota in patients with cirrhosis improves clinical outcomes. Rifaximin-α was approved by NICE for the prevention of the recurrence of overt hepatic encephalopathy in cirrhosis (9) but considerable concern remains regarding whether long term antibiotic prescription will result in a change in bacterial function and virulence rather than a simple reduction in bacterial population and whether this may drive bacterial resistance to antibiotics in an already functionally immunocompromised population. The question was therefore raised as to whether directly, as opposed to indirectly modulating the gut microbiota utilising faeces from healthy donors may be a safer and more durable therapy. Faecal microbiota transplantation (FMT) has been licensed by NICE since 2014 for the treatment of recurrent *Clostridium difficile* infection (10). FMT has shown promising results in clinical trials of several disease states resulting from gut dysbiosis beyond *Clostridium difficile* infection (10) for example in ulcerative colitis (11-14).

We hypothesise that in patients with advanced cirrhosis, FMT may reduce the progression to chronic liver failure including the development of jaundice, ascites, bleeding, encephalopathy, infection and organ dysfunction. Whether FMT is feasible in the setting of cirrhosis remains to be investigated. We propose conducting a feasibility trial to determine whether FMT from a healthy donor will alleviate gut dysbiosis and immune dysfunction in advanced cirrhosis.

Methods and Analysis

Primary Objectives:

The primary objective of this study will be to assess whether stabilising gut dysbiosis with FMT in patients with advanced cirrhosis is both feasible and safe.

Primary Endpoints:

The primary endpoints of the study will be twofold. To assess the feasibility of FMT as determined by the recruitment rates (including acceptability of the intervention) and tolerability of FMT e.g. gastro-oesophageal reflux rates. The second primary outcome measure will be to assess the safety of FMT administration, including the incidence of any transmissible bacterial or viral infection that is deemed to have been acquired from the donor including *Clostridium difficile* infection.

Secondary Objectives:

The secondary objectives of the study are to provide preliminary evidence of efficacy for a larger randomised trial, with the purpose of choosing the optimal primary outcome, and estimating the parameters for sample size calculation. We will also collect blood, stool and urine samples from participants to assess the stability of the transplanted gut microbiome by

For peer review only

comparing the percentage composition of the stool microbiota on day 7, 30 and 90 with the donor microbiome. Plasma endotoxin (and endotoxin binding protein), pro- and anti-inflammatory cytokine levels, bacterial DNA quantification and serum procalcitonin will be performed at 7, 30 and 90 days as will changes in faecal biomarkers (calprotectin, lactoferrin and M2-Pyruvate Kinase).

Trial Design

PROFIT is a single-centre study. Thirty-two patients will be recruited from out-patient clinics at King's College Hospital (KCH) or from suitable inpatients on the wards. The patients will be recruited as per the inclusion and exclusion criteria listed in table 1. Patients will be randomized in a single-blinded fashion in a ratio of 3:1 FMT to placebo. Patients will be unaware of the intervention given, but investigators will not be blinded to the treatment intervention.

Patient and Public Involvement

A lay reviewer/patient representative at the NIHR reviewed the protocol. This was funded by the Research for Patient Benefit programme. Feedback was taken on board and revisions were subsequently made.

The British Liver Trust, British Society of Gastroenterology Liver Research Group and King's Liver Outpatient Advisory Group (patient group) were all involved in the study set-up and protocol development at all stages.

Results will be disseminated to study participants via the Liver Research Nurse if they indicate an interest in the study outcome.

Table 1- Inclusion and exclusion criteria

Inclusion Criteria
• 18–75 years<br>• Confirmed advanced cirrhosis of any aetiology with a MELD (15) score between 10 and 16. The diagnosis of cirrhosis will be based on clinical, radiological, or histological criteria.<br>• Patients with alcohol-related liver disease must have been abstinent from alcohol for a minimum of 6 weeks.<br>• Patients must be deemed to have capacity to consent to the study.

For peer review only

Exclusion Criteria

- Severe or life-threatening food allergy
- Pregnancy or breastfeeding
- Patients treated for active variceal bleeding, infection, bacterial peritonitis, overt hepatic encephalopathy or acute-on-chronic liver failure within the past 14 days.
- Patients who have received antibiotics in the past 14 days.
- Active alcohol consumption of >20 grams/day.
- Has had a previous liver transplant
- Hepatocellular carcinoma outside of the Milan Criteria (16)
- Inflammatory bowel disease
- Coeliac disease
- A history of prior gastrointestinal resection such as gastric bypass
- Patient is not expected to survive the duration of the study (90 days).
- Severe renal impairment (creatinine >150 µmol/L)
- HIV positive
- Immunosuppression e.g. more than two weeks treatment with corticosteroids within 8 weeks of intervention, active treatment with tacrolimus, mycophenylate, azathioprine.

For peer review only

Patient Population

The study will include all patients aged 18 to 75 years with capacity to consent and a diagnosis of cirrhosis. Patients will have a MELD (15) score of between 10 and 16. The diagnosis of cirrhosis may be based on radiological, clinical or histological parameters. In the case of alcohol-related cirrhosis, patients must have been abstinent for a minimum of six weeks. The exclusion criteria are outlined in table 1. Anticipated recruitment is outlined in Figure 1 below (17).

Consent

All participants will be asked to provide informed consent after having had the opportunity to discuss the trial with the clinician, their family and friends and having read the patient information sheet (PIS- see supplementary file.) Patients who lack capacity will not be enrolled in this study. If patients lose capacity during the trial follow up period, an appropriate legal representative will be consulted and will provide consent to ongoing trial participation after appropriate discussion with the trials team and having read the PIS. If the legal representative does not agree to ongoing trial participation, the subject will be withdrawn from trial follow up.

Figure 1 Study Flow Chart and Anticipated Recruitment

Study Intervention

FMT is prepared in a laboratory at St Thomas' Hospital in accordance with the principles of Good Manufacturing Practice (GMP) and under Manufacturing Authorisation for an Investigational Medicinal Product (MIA(IMP)) from the Medicines and Healthcare products Regulatory Agency (MHRA). FMT donors are healthy volunteers with no medical problems and normal body mass index. They must not be taking any regular medications and are rigorously screened for blood borne and enteric pathogens prior to donation. Donors undergo questionnaire screening for risk factors at baseline and again at donation to reduce the risk of transmissible infection as outlined in table 2 (full questionnaire in supplementary materials). Stool is processed (diluted and filtered with the addition of glycerol) within 6 hours of donation and stored at -80 degrees Celsius for up to six months (as previously described) (18). Material for FMT has full traceability from donor to recipient and aliquots of donor stool are kept for 30 years to allow further testing in the case of any adverse events. Placebo is a solution of saline with glycerol without faecal matter.

Table 2 blood and stool testing of donor FMT samples

Blood (serology)

- HIV 1+2 serology
- HTLV I/II Ab
- Hepatitis A IgG (and if positive IgM)
- Hepatitis B surface antigen (HBsAg) and core antibody
- Hepatitis C (HCV Ab)
- Hepatitis E
- Syphilis
- CMV/EBV IgG/M
- Strongyloides stercoralis (ELISA)

Stool

- PCR for gastroenteritis agents (*Campylobacter, Salmonella, Shigella* and *E. coli* O:157)
- Ova, cysts and parasites by concentration and microscopy x3
- *C. difficile* test
- Norovirus PCR
- Screen for gentamicin and carbapenem resistant Gram negative organisms
- Screen for MRSA
- Helicobacter pylori antigen
- Entamoeba histolytica PCR

For peer review only

Following identification, all patients will attend a screening visit and sign the informed consent form. If deemed to be eligible they will attend for a baseline visit with full clinical history and examination. Medication history will be recorded. Patients will complete a dietary questionnaire. Baseline samples of blood, urine, saliva and stool will be obtained. All patients will subsequently attend for a gastroscopy at which the FMT or placebo will be administered under direct visualization into the jejunum via a nasojejunal tube. This will be performed as per the local Endoscopy Unit Protocol using topical local anaesthetic spray or midazolam sedation as per patient preference. Patients will first be asked to take bowel preparation to purge the bowel of its native bacteria. This consists of two sachets of Moviprep® taken prior to gastroscopy. Patients will be monitored for side effects in the Clinical Research Facility after the procedure.

Evaluations during and after treatment

Participants will be reassessed at 7 (+/- 5), 30 (+/-7) and 90 (+/-7) days post intervention. They will be reviewed in the Clinical Research Facility and undergo physical examination, review of medications and dietary changes and adverse event monitoring. Samples of blood, urine, saliva and stool will be taken at these visits also. At the end of the 90 day follow up period, patients will return to their usual care pathway.

Statistical analysis

Sample Size

This feasibility study will evaluate feasibility parameters using 95% Confidence Intervals. The sample size has been proposed mainly to enable the trial to be conducted within the allocated budget and with acceptable precision for continuous outcomes. According to the simulation work by Teare MD et al. (18), even with the relatively small pilot sample size of 20, the planned studies would have at least 80% power to detect the target effect size (for continuous outcomes) more than 75% of the time. Teare et al. recommend that 60 to 100 subjects are sufficient to estimate an event rate (such as recruitment rates) with acceptable precision in a feasibility study, while sample sizes between 24 and 50 have been recommended for the accurate estimation of standard deviations. Therefore, we have chosen a sample size of 32 patients in this trial to have reliable data on all critical parameters (including event rates) which can also be utilised when planning a larger intervention trial. For event rates (e.g. recruitment rates) and particularly in the extreme case with lower rates e.g. 10%, we estimate a drop of precision by only 5% using our updated sample size and the minimum recommended by Teare et al. (0.16 for 60 patients vs 0.21 for 32 patients). Figure 2 below illustrates the

reduction in precision of different rates when the sample size increases for binary outcomes. This sample size will also be feasible within the budget and will provide acceptable information for planning a future large clinical trial.

Figure 2 Confidence interval width around one proportion (P) by sample size (N) from PASS software REF - PASS 15 Power Analysis and Sample Size Software (2017). NCSS, LLC. Kaysville, Utah, USA, ncss.com/software/pass.

The sample size of 32 patients will undergo randomisation in a 3:1 ratio. This will allow the study to demonstrate feasibility of randomising, yet providing robust evidence with respect to the feasibility of the treatment, and preliminary evidence of efficacy parameters.

The following feasibility criteria have been established:

- 25% of screened patients will consent: 256 patients will allow the estimate of the 2-Sided 95% confidence interval of the proportion of consented patients, where the distance from the observed proportion to the limit is 0.058 units.
- 50% of patients screened will fulfil inclusion/exclusion criteria for the trial: screening 64 patients will allow the estimate of the 2-Sided 95% confidence interval of the proportion of patients eligible, where the distance from the observed proportion to the limit is 0.128 units.
- 80% of randomised patients will complete treatment and follow up: 26 of 32 patients completing will allow the estimate of the 2-Sided 95% confidence interval for a single proportion, where the distance from the observed proportion to the limit is 0.177 units.

Differences between the placebo and treatment group in efficacy binary outcomes, will be estimated along with the two-sided 95% CI. Assuming the proportion with the outcome in the control group is 0.5 our sample size will allow estimation of the half-width of the confidence intervals for different size of differences as indicated below. Differences of 0.4 or higher will be distinguishable from 0.

Table 3 Half width of confidence intervals for differences in proportions between two groups with given sample size, placebo group p=0.5 (created with PASS software REF - PASS 15 Power Analysis and Sample Size Software (2017). NCSS, LLC. Kaysville, Utah, USA, ncss.com/software/pass.)

Difference	0.1	0.2	0.3	0.4	0.5
Width	0.398	0.392	0.382	0.337	0.347

The half-width of the 95% CI of the difference in means for continuous outcome will be 0.889 SD.

For peer review only

For peer review only - http://bmjopen.bmj.com/site/about/guidelines.xhtml

Clinical Endpoints

Clinical and safety events will be listed and summarised by intervention group. MELD (15) scores will be calculated by visit and treatment group.

Data synthesis, analysis and presentation

Feasibility and efficacy outcomes will be summarised using the appropriate descriptive statistics, and 95% Confidence Intervals will be calculated to allow for success and go/no go decisions. Biomarker data will be pre-processed according to the established standards for each platform, and statistical analyses will be performed using non-parametric and permutation based methods which are more appropriate for small sample sizes.

Statistical Software

Analyses will be performed using R and/or Stata statistical software packages.

Discussion

The PROFIT study is the first study to look at the use of FMT delivered directly into the small bowel in patients with cirrhosis. Liver transplantation is a highly selective treatment and only those most likely to benefit will be listed. Depending on blood type patients can wait up to 24 months for a liver transplant. There are even fewer options for patients who are unsuitable for transplantation.

Cirrhosis is often complicated by recurrent admissions with sepsis, variceal bleeding, ascites and hepatic encephalopathy. We know that patients with cirrhosis have enteric dysbiosis and altered gut permeability. The non-absorbable antibiotic rifaximin has proved useful in patients with cirrhosis and chronic hepatic encephalopathy, but the long-term benefits of antibiotic use are unknown and the possibility of development of bacterial resistance remains with all antibiotics. The possibility of re-populating the gut of cirrhotic patients with healthy gut microbiota could be a potential alternative, providing a new treatment approach for these vulnerable individuals.

A group in the USA have recently published the results of the impact of FMT delivered via enema in 10 cirrhotic patients, compared with 10 patients receiving standard of care treatment (19). FMT was found to be safe and the patients in the treatment group did not have any admissions for HE, whereas there were six episodes of HE in the standard of care group. Four of these episodes required hospital admission. The FMT group were treated with broad spectrum antibiotics prior to FMT administration, whereas the standard of care group were not, meaning that the effects of FMT cannot be clearly separated from the antibiotic administration. We hope that PROFIT will add to the knowledge base of the use of FMT in cirrhosis and will provide an accurate assessment of the safety of FMT. We plan to treat the FMT and control groups identically, aside from the administration of FMT to accurately assess its impact, without the confounding factor of antibiotic treatment.

A limitation of our study is the single-blinded design. This was selected due to the inherent difficulties in preparing a matched placebo, without introducing substances that may upset

For peer review only

the delicate gut microbiota. The IMP will be delivered by the trial investigators, so it has not been possible to blind the clinicians in this study. Patients and the trial statistician will be blinded.

The study is not powered to detect differences in clinical outcomes, but may provide evidence for markers relating to clinical outcomes that could be studied in a larger RCT.

Sponsor

The PROFIT trial is co-sponsored by King's College Hospital and Kings' College London. The funding was supplied by an NIHR research grant for patient benefit (PB-PG-0215-36070).

Trial Monitoring Groups

Trial Steering Committee (TSC)

This group will oversee the running of the trial and discuss any issues that may arise throughout the process of recruitment and follow up of patients. The group will be chaired by an independent clinician. Investigators will report to the group on a regular basis. The Data Monitoring Committee (DMC) will inform the TSC if there are any issues raised from its discussions.

Data Monitoring Committee

This is an entirely independent group that analyzes interim data, to determine whether or not the trial is safe to continue. It monitors adverse events and adverse reactions and reacts to any issues and directs the TSC as to whether or not the trial should continue. The DMC undertakes interim statistical analysis using an independent statistician to ensure the ongoing safety and integrity of the trial. The members of this committee are independent of the trial, but will be experienced clinicians with expertise in clinical trials.

Ethics and Dissemination

The ethical permission has been given by the South-East Research Ethics Committee, REC reference 17/LO/2081, IRAS project ID 197237. EUDRACT number 2017-003629-13.
The results of the trial will be analyzed and published in a peer reviewed journal and disseminated at international conferences.

Funding statement

This work was supported by the NIHR, grant number PB-PG-0215-36070. This paper presents independent research funded by the National Institute for Health Research (NIHR) under its Research for Patient Benefit (RfPB) Programme (Grant Reference Number PB-PG-0215-36070). The views expressed are those of the author(s) and not necessarily those of the NHS, the NIHR or the Department of Health & Social Care.

For peer review only - http://bmjopen.bmj.com/site/about/guidelines.xhtml

Competing Interests Statement

Dr Shawcross has received fees from Norgine, Falk and Shinogi-, outside the submitted work. Dr Goldenberg reports grants and personal fees from Astellas, personal fees from MSD, personal fees from Pfizer, personal fees from Shinogi, outside the submitted work.

Acknowledgements

We would like to thank the clinicians involved, King's College Hospital R&D Department, the NIHR, patient advisers and lay reviewers for their contributions in the set-up of the PROFIT trial. We would also like to acknowledge Liz Allen, our QP, for her contribution to the MHRA approval process.

Author Contributions

CW and DS wrote the manuscript, with assistance from SG, VP and ASF. AD and CF prepared the statistical analysis plan. LC and AO'B advised on study analyses and the format of the protocol for submission. SG provided the expert advice on FMT and has set up the FMT service at GSTT, which will supply the FMT to the study. All authors reviewed and edited the manuscript prior to submission.

For peer review only

1. Wiest R, Garcia-Tsao G. Bacterial translocation (BT) in cirrhosis. Hepatology. 2005;41(3):422-33.
2. Jalan R, Fernandez J, Wiest R, Schnabl B, Moreau R, Angeli P, et al. Bacterial infections in cirrhosis: a position statement based on the EASL Special Conference 2013. J Hepatol. 2014;60(6):1310-24.
3. Bajaj JS, Heuman D. M., Hylemin P.B. Sanyal A. J., White M. B. <Altered profile of human gut microbiome is associated with cirrhosis and its complications>. Journal of Hepatology. 2014;60:940-7
.
4. Nolan JP. The role of intestinal endotoxin in liver injury: a long and evolving history. Hepatology. 2010;52(5):1829-35.
5. Qin N, Yang F, Li A, Prifti E, Chen Y, Shao L, et al. Alterations of the human gut microbiome in liver cirrhosis. Nature. 2014;513(7516):59-64.
6. Bajaj JS, Heuman DM, Sanyal AJ, Hylemon PB, Sterling RK, Stravitz RT, et al. Modulation of the metabiome by rifaximin in patients with cirrhosis and minimal hepatic encephalopathy. PLoS One. 2013;8(4):e60042.
7. Shawcross DL. Is it time to target gut dysbiosis and immune dysfunction in the therapy of hepatic encephalopathy? Expert Rev Gastroenterol Hepatol. 2015;9(5):539-42.
8. Patel V OJ, Sturgeon J, Habtemariam Z, Preedy H, Richardson P, et al. Rifaximin Is Efficacious In The Treatment Of Chronic Overt Hepatic Encephalopathy: A UK Liver Multi-Centre Experience [Abstract]Suppl 1 Gut 2014;63.
9. NICE. National Institute for Health and Care Excellence. Rifaximin for the maintenance treatment of hepatic encephalopathy. 2015 Mar. Report No.: ID496.
.
10. NICE. National Institute for Health and Care Excellence. Faecal microbiota transplant for recurrent Clostridium difficile infection. 2014 Mar. Report No.: Interventional Procedure Guidance 485.
11. Sudarshan Paramsothy MAK, Nadeem O Kaakoush, Alissa J Walsh, Johan van den Bogaerde, Douglas Samuel, Rupert W L Leong, Susan Connor, Watson Ng, Ramesh Paramsothy, Wei Xuan, Enmoore Lin, Hazel M Mitchell, Thomas J Borody. <Multidonor intensive faecal microbiota transplantation for active UC; a randomsied placebo controlled trial.pdf>. Lancet. 2017;389:1218-28.
12. Paramsothy S, Paramsothy R, Rubin DT, Kamm MA, Kaakoush NO, Mitchell HM, et al. Faecal Microbiota Transplantation for Inflammatory Bowel Disease: A Systematic Review and Meta-analysis. J Crohns Colitis. 2017;11(10):1180-99.
13. Rossen NG, Fuentes S, van der Spek MJ, Tijssen JG, Hartman JH, Duflou A, et al. Findings From a Randomized Controlled Trial of Fecal Transplantation for Patients With Ulcerative Colitis. Gastroenterology. 2015;149(1):110-8 e4.
14. Moayyedi P, Surette MG, Kim PT, Libertucci J, Wolfe M, Onischi C, et al. Fecal Microbiota Transplantation Induces Remission in Patients With Active Ulcerative Colitis in a Randomized Controlled Trial. Gastroenterology. 2015;149(1):102-9 e6.
15. Kamath PS, Wiesner RH, Malinchoc M, Kremers W, Therneau TM, Kosberg CL, et al. A model to predict survival in patients with end-stage liver disease. Hepatology. 2001;33(2):464-70.

For peer review only

16. Mazzaferro V RE, Doci R, Andreloa S, Pulvirenti A, Bozzetti F, Montalto F, Ammatuna M, Morabito A, Gennari L Liver transplantation for the treatment of small hepatocellular carcinomas in patients with cirrhosis. New England Journal of Medicine. 1996;334(11):693-700.
17. DL S. RANDOMISED CLINICAL FEASIBILITY TRIAL PROTOCOL PROFIT: A PROspective, randomised placebo controlled feasibility trial of Faecal mIcrobiota Transplantation in cirrhosis
. 2015.
18. Goldenberg SD BR, Beales I, Digby-Bell J, Irving P, Kellingray L, Narbad A, Franslem-Elumogo N. Comparison of different strategies for providing fecal microbiota transplantation to treat patients with recurrent Clostridium difficile infection in two English hospitals: a review. Infectious Diseases and Therapy 2018.
19. Bajaj JS, Kassam Z, Fagan A, Gavis EA, Liu E, Cox IJ, et al. Fecal Microbiota Transplant from a Rational Stool Donor Improves Hepatic Encephalopathy: A Randomized Clinical Trial. Hepatology. 2017.

For peer review only

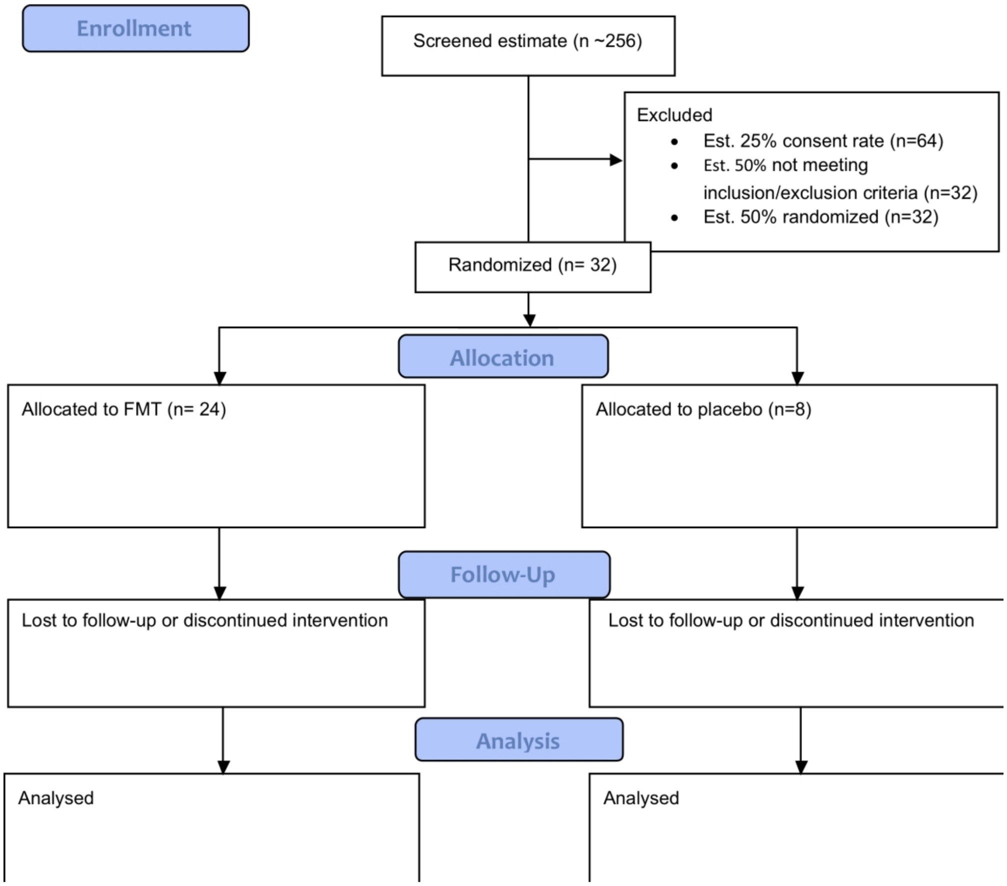

Figure 1 Study Flow Chart and Anticipated Recruitment

190x212mm (300 x 300 DPI)

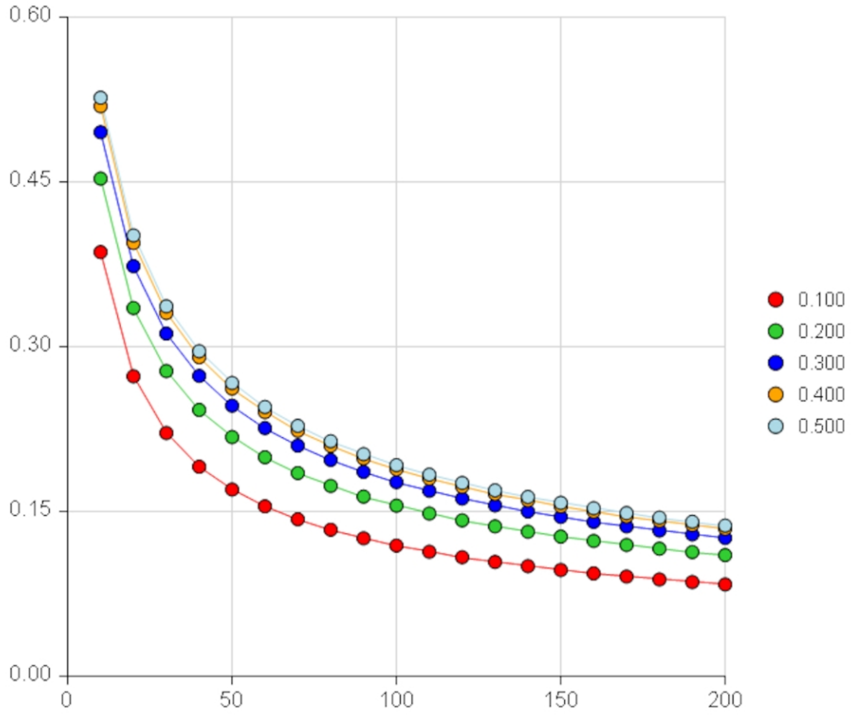

Figure 2 Confidence interval width around one proportion (P) by sample size (N) from PASS software

101x90mm (300 x 300 DPI)

King's College Hospital NHS
NHS Foundation Trust

Participant Information Sheet

Short Title: **PROFIT**

Full Title of Study: **A Prospective, randomized placebo controlled feasibility trial of faecal microbiota transplantation in cirrhosis**

Chief Investigator: **Dr D. Shawcross**

Researchers: **Dr C Woodhouse, Dr V. C. Patel, Prof A. Sanchez-Fueyo,**

For peer review only

Introduction

You are invited to take part in this research study. Before you decide it is important for you to understand why the research is being done and what it will involve. Please take time to read the following carefully and discuss it with friends, family or your GP if you wish. Ask us if there is anything that is not clear or if you would like more information. Take time to decide whether or not you wish to take part.

What is the purpose of the study?

Our bodies contain trillions of bacteria that live in our bowels. These bacteria help to keep us healthy. Patients with liver cirrhosis have more 'unfriendly' bacteria than people without cirrhosis. Patients with cirrhosis also have a 'leaky' gut, allowing these bacteria to leak into the blood stream, potentially causing serious infections. These can usually be treated with antibiotics, but may be very severe and may also cause kidney failure, hepatic encephalopathy (a condition where toxins build up in the blood stream, causing confusion and even coma) and potentially life threatening bleeding.

The treatment proposed in our study, FMT (faecal microbiota transplantation,) takes the bacteria from the intestines of healthy volunteers and replaces the abnormal bacteria in the gut of a patient with cirrhosis. The aim of faecal microbiota transplantation (FMT) is to replace the 'unfriendly' bacteria with 'friendly' bacteria from healthy volunteers, therefore preventing them from getting into the blood stream and causing serious problems.
Volunteers are screened very thoroughly (in a similar way to blood donors) to ensure that they are healthy and not liable to pass on any infections to the recipients of the FMT.

FMT has been tested in a small group of patients with cirrhosis in America and was found to be safe, and has also been used more widely in other conditions such as patients with a gut infection called 'clostridium difficile' and patients with inflammatory bowel disease. It is extremely successful in these patients and we would like to see if it can be as successful in liver patients.

Results from this study would support further research into the benefit of using FMT to lessen infection rates in patients with liver cirrhosis who have developed bowel problems.

What groups of patients will the study involve?

We are interested in involving people in this study who have liver cirrhosis of any cause.

For peer review only - http://bmjopen.bmj.com/site/about/guidelines.xhtml

King's College Hospital NHS
NHS Foundation Trust

Why have I been invited?

All patients who are admitted to the Liver wards and/or seen in the Liver out-patient clinics at King's College Hospital NHS Foundation Trust who have liver cirrhosis may be eligible to participate in this study. This also includes patients that are awaiting consideration for or have already been listed for liver transplantation

Do I have to take part?

No, participation is entirely voluntary. It is up to you whether or not you wish to take part. If you decide to take part you will be given this information sheet to keep and asked to sign a consent form. If you decide to take part you are still free to withdraw at any time and without giving a reason. This will not affect the medical care you will receive.

Who is organising and funding this study?

The doctor in charge of this study is Dr Debbie Shawcross. The study is funded by the National Institute of Health Research and is being sponsored by King's College London and Kings College Hospital NHS Foundation Trust.

Who has reviewed the study?

All research in the NHS is looked at by an independent group of people, called a Research Ethics Committee, to protect your interests. This study has been reviewed and approved by the London South East Research Ethics Committee. It has also been approved by the Health Research authority and the Medicines and Healthcare products Regulatory Agency.

What will happen to me if I take part (SUMMARY)?

If you decide to take part you will be randomly assigned to one of two groups. One group (the 'active treatment' group) will receive Faecal Microbiota Transplantation (FMT) whilst the other group (the comparison or 'control group') will receive a 'placebo' treatment. Administration of placebo or FMT will take place only ONCE during this study.

The placebo treatment is a 'dummy' treatment given to participants in the control group of a clinical trial. This method using a 'placebo control' is used to make sure the study results are reliable by checking that any changes measured are actually due to the effect of the active drug and not just because of chance. The placebo in this trial is a solution of saline and glycerol (salty water and a preservative, which are the same ingredients used in the FMT itself, just without the FMT.)

Both groups will then be followed up to see how effective the active treatment is based on various measurements taken. Blood, urine, saliva and stool samples will be obtained for tests at four specific times. Firstly when starting the study at 'baseline' (just before the FMT or placebo is given,) and then repeated at 7, 30 and 90 day intervals after treatment has been administered. Follow-up appointments at the 7, 30 day and 90 days after starting will be arranged to coincide as far as possible with planned routine medical appointments.

For peer review only

For peer review only - http://bmjopen.bmj.com/site/about/guidelines.xhtml

King's College Hospital NHS
NHS Foundation Trust

Study procedures	Screening	Baseline (-7 days)	Endoscopy	Day 7 (+/- 5days)	Day 30 (+/- 7 days)	Day 90 (+/- 7 days)
Signed informed consent	X					
Eligibility Criteria	X					
Participant demographics	X					
Medical and surgical histories	**X**			X	X	X
Dietary questionnaire		X		X	X	X
Medication usage		X		X	X	X
Clinical examination		X		X	X	X
Blood sampling		X		X	X	X
Stool sampling		X		X	X	X
Saliva sampling		X		X	X	X
Urine sampling		X		X	X	X
Randomisation		X				
FMT/placebo administration			X			
Adverse events monitoring /Safety			X	X	X	X

As part of the study you will have an endoscopy (a camera test looking into your stomach/small bowel) to give you the FMT or the placebo treatment. This means that you will not have to drink the liquid as it is placed directly into the small bowel where it is needed, avoiding the stomach acid, which could stop it working. Before the endoscopy you will be given a medicine to clear your own gut bacteria called 'Moviprep.' This will give you loose bowel motions, which is a normal reaction to the Moviprep. This is essential to clear your own gut bacteria from the bowel to allow the FMT to work.

We will see you after the endoscopy at days 7, 30 and 90 to take blood, urine, saliva and faecal samples from you. We will also assess your general wellbeing and check that your medicines have not changed. Blood samples will usually be taken at the time of when you are having tests done as part of your routine clinical care, to reduce the number of times you have to have a needle in your arm and, where possible, samples will be taken from tubes already in your blood vessels to minimise discomfort to you.
We will ask you to collect the urine and stool samples in the morning of each study visit and bring this with you when you attend clinic. We will ensure you are given all the necessary collection pots for this. After the last visit at 90 days, you will return to the usual standard of care as you were having for you underlying condition.

More detailed information about the study visits can be found on page 7 of this information sheet.

What are the side effects of taking part?

For peer review only - http://bmjopen.bmj.com/site/about/guidelines.xhtml

If you are assigned to the active treatment group and receive FMT, there are some possible side effects that you should be aware of. Faecal transplants have not been widely used in patients with cirrhosis yet, but have been used in lots of patients with an antibiotic associated diarrhoea called 'clostridium difficile.' Small numbers of patients in these studies reported some abdominal distension/bloating, flatulence, diarrhoea or constipation as side effects of the treatment. Part of this study is to assess how well tolerated the treatment is and whether or not patients with cirrhosis experience similar symptoms.

Side effects of blood sampling can include bruising and it can be uncomfortable. However, every effort will be made to perform blood sampling at the same time as regular blood tests to minimise any discomfort. There should be no side effects of sampling your urine and stool as we will simply ask you to collect the samples when you go to the toilet as normal. Saliva will be collected by asking you to rinse your mouth out with water and to spit into a collection pot.

What are the possible disadvantages and risks of taking part?

We hope that the knowledge we would gain from this study will improve our understanding of the way in which FMT works, and the role of the gut and immune system in liver cirrhosis. Patients who received FMT in a small study in America did well and had fewer hospital admissions than those in the 'standard of care' group. FMT appears to be safe in the considerable numbers of patients who have received it for other reasons. There are no disadvantages or risks involved in taking part with regard to the samples being obtained, and the amount of blood taken will not be harmful to you. The placebo treatment is not harmful, but is not expected to have any benefit on your clinical condition as it contains no active drug or therapy. All patients, regardless of whether they receive placebo or FMT have the same rigorous follow up and support from the trials team.

You will be closely monitored after the treatment is given and will be seen at regular intervals afterwards to see if you have experienced any side effects. We do not anticipate any serious problems to occur after the FMT or placebo treatment, but you will be given contact numbers should you run into any problems outside of the trial visits.

Apart from the side effects of FMT, all patients (those on placebo and active treatment) will need to take bowel preparation, which can cause dehydration. The endoscopy itself also has a small risk of bleeding, perforation (this is very rare and is thought to occur in around only 1 in 2000 tests) and damage to teeth and dental work. There is also a risk of aspiration of the FMT or placebo into the lungs, therefore you will be monitored closely after the procedure to monitor for this.

What are the possible benefits of taking part?

You will be taking part in a new and innovative trial to assess the effect of FMT on cirrhosis. There are few treatments available to patients with cirrhosis, aside from liver transplantation. Unfortunately due to the scarcity of donor organs, even those listed for transplant can wait months or even years for a new liver. Others are too unwell or frail to be listed for transplant. We hope that looking at FMT in more detail may provide a much needed treatment for patients with cirrhosis who are not suitable for transplant or to help those awaiting a new liver. This study is designed to look at the safety and tolerability of the treatment to see if a larger study would show an improvement in wellbeing for these patients.

For peer review only - http://bmjopen.bmj.com/site/about/guidelines.xhtml

What if I lose the ability to provide consent after enrolling to the study ('loss of capacity)?

In the event that you lose the ability to provide ongoing consent during the research due to your illness, having been able to provide consent at the start of the study, we will approach an appropriate relative or friend on your behalf to ask them if they think that you would have wanted to continue participation in this study. This person is termed a 'Consultee' and they are asked to set aside their own views and provide advice on you continuing to participate in the research, taking into consideration your wishes and interests.

Any advance decisions you may have made, and that they are aware of, should take precedence. If the Consultee decides that you would have no objection to continue taking part they will be asked to read and sign a consultee declaration form. They will then be given a copy to keep. If they decide that you would not wish to continue taking part we will withdraw you from the study; this will not, in any way, affect the standard of care you receive. If the Consultee we approach does not feel able to take on this role they may want to identify someone else to do it. They will be asked to take time to decide whether or not they feel that you would wish to continue taking part. They will be encouraged to ask the research team if there is anything that is not clear or if they would like more information. The information they will be provided with is the same as that provided to you.

You may wish to identify a suitable Consultee at this stage given the above, so that if you do lose the ability to provide consent after you are enrolled (should you decide to participate in the study) we can approach a person of your choice.

What if something goes wrong?

If you have a concern about any aspect of this study, you should ask to speak to your study doctor who will do their best to answer your questions (contact details on page 8) If you remain unhappy and wish to complain formally, you can do this through the NHS Complaints procedure by contacting your local Patient Advice Liaison Service (PALS) office. Details of your local office can be obtained by asking your study doctor, GP, telephoning your local hospital or looking on the NHS choices website. http://www.nhs.uk/pages/home.aspx

Every care will be taken in the course of this study. However in the unlikely event that you are injured by taking part, compensation may be available.

In the event that something does go wrong and you are harmed during the research and this is due to someone's negligence then you may have grounds for a legal action for compensation against King's College Hospital NHS Foundation Trust & King's College London, but you may have to pay your legal costs.

Regardless of this, if you wish to complain, or have any concerns about any aspect of the way you have been approached or treated by members of staff or about any side effects (adverse events) you may have experienced due to your participation in the study the normal National Health Service

For peer review only - http://bmjopen.bmj.com/site/about/guidelines.xhtml

King's College Hospital NHS
NHS Foundation Trust

complaints mechanisms are available to you. Please ask your study doctor if you would like more information on this.

Will my taking part in this study be kept confidential?

Yes. Once you have consented to take part in the study, you will be given a unique study number, which will be used to identify any samples and information collected during the research.

Some information regarding you and your condition will be recorded as part of this study. We will not record any personal identifiable information (name, date of birth or contact details) as part of the research records. All information will be stored anonymously in a password-protected database, or in a file in a secure research office. Only your treating doctors and the research team will have access to this information.

We will inform your General Practitioner (GP) of your participation in the study - this is routine for this type of research and helps them to care for you during the trial. We advise that you inform your private medical insurance provider (if you hold private medical insurance) of your participation in this study - this again is routine procedure for this type of research.

What will happen to the results of the research study?

When we have results for an adequate number of patients after the study is completed we plan to publish them in an international journal so that the information can benefit as many people as possible. We can provide you with a brief summary of the results of the study when available should you desire this.

What will happen to any samples that I give?

Every effort will be made to ensure blood samples for the study are taken at the same time as samples are taken as part of your routine clinical care – so that there will be no additional discomfort to you as a result of being involved in this study. The amount of blood taken per time point for the study is approximately 50mls or 10 teaspoonfuls. This is a small amount for the body (which contains about 5L of blood, or 5000mL) and will not adversely affect you in any way.

Samples of blood and other fluids obtained may be used immediately or saved for human or bacterial DNA or immune system analysis in the future, in the Liver Biobank. Some of the actual tests on the samples that are obtained as a result of you taking part in the study will be performed on site at King's College Hospital NHS Foundation Trust, whilst some samples will be transferred to other collaborator laboratories in the UK and France. At all times any samples that are transferred and stored will be coded anonymously regardless of which laboratory they are sent to.

Contact details:

Thank you for taking the time to read this information.

If there is any other information you would like, please do not hesitate to contact us on the numbers below. Out of hours or if a response on the above contact number is unavailable, it is possible to contact the on-call Hepatology Registrar – via KCH Switchboard – who will pass any messages on to the senior medical staff involved.

For peer review only - http://bmjopen.bmj.com/site/about/guidelines.xhtml

King's College Hospital NHS
NHS Foundation Trust

Principal investigator: Dr Debbie Shawcross Tel: 02032992504
Clinical research fellow: Dr C Woodhouse Tel: 02032992504
Research nurse: Ane Zamalloa Tel: 0203299 7623

For peer review only

For peer review only - http://bmjopen.bmj.com/site/about/guidelines.xhtml

King's College Hospital NHS
NHS Foundation Trust

What will happen to me if I take part (DETAILED EXPLANATION)?

You will be assessed for your eligibility to be involved in this study, and if you are happy to proceed will sign the consent form with a member of the research team. You will then be randomly allocated to one of two treatment groups:

1) the 'active treatment' group: participants in this group will receive the treatment being tested (FMT), or
2) the comparison or 'control group'; participants in this group will receive a 'placebo' treatment, which is a 'dummy' treatment and is indistinguishable from the actual treatment given to participants in the 'active treatment' group.

Before starting treatment with either of the above, you will undergo baseline assessments, which involve the following (those marked with * are being done as part of the study):

- Medical history including current medication usage.
- Physical examination including blood pressure, pulse rate, height, weight.
- Dietary questionnaire* and quality of life questionnaire*
- Blood sampling: will include tests of blood counts, kidney and liver function performed as part of your routine clinical care as well as checks for various viruses that can affect the liver.
- Blood sampling*: to measure endotoxin levels, immune function including DNA analysis and metabolic profiling.
- Urine sampling*: we will ask you to provide a urine sample (approx. 20mls) to measure metabolic profiling. If you are a woman of child-bearing age, we will also do a pregnancy test.
- Stool sampling*: we will ask you to provide stool samples to measure what types of bacteria are found, and in what patterns and quantities.
- Salivary sampling: *: we will ask you to provide saliva samples from your mouth to measure what types of bacteria are found, and in what patterns and quantities.
- If you have fluid in your abdomen called "ascites" we would like to take a sample of the fluid to check for infection. This involves passing a small needle into your abdomen under aseptic conditions. This test is voluntary and you do not have to take part, but it would help us to check for infection in the fluid and would mean we could treat any infection early, should one be present*.

After the baseline assessments have been completed, you will come up to the endoscopy unit to have the FMT or placebo treatment given. The procedure will be explained to you and the risks and benefits discussed. Endoscopy is a safe test, but like all procedures has some risks. The main risks to be aware of are a small risk of bleeding and the risk of perforation (1 in 9000.) There is also a risk of damaging teeth or dental work and a small risk of 'aspiration' which is where fluid from the stomach goes into the lungs. This is why we ask you not to eat for six hours prior to the test, to make sure the stomach is empty. Some people also develop a sore throat after the procedure. This should get better on its own.

You will also be given a medicine prior to the test called 'Moviprep' to help clear your bowels of their usual bacteria prior to the treatment with FMT or placebo. This is a safe medicine, but will cause you to have loose stools. This is essential to its function, but it is important you drink

For peer review only - http://bmjopen.bmj.com/site/about/guidelines.xhtml

plenty of fluid to prevent dehydration. Side effects can include dehydration, nausea and vomiting.

You will take one sachet of Moviprep mixed with a litre of water at 19.00 the night before the test. You can then have a light meal. On the morning of the endoscopy you will drink another sachet of Moviprep mixed with one litre of water at around 7.00am. You can take your usual medications as normal. There is a lot of fluid to drink, so you may find it easier if it is chilled or mixed with a squash (not blackcurrant) to make it easier to drink.

You will undergo similar assessments to the baseline visit at 7, 30 and 90 day intervals as you did just before starting treatment. You will in addition undergo the following as part of the study:

- Study treatment adverse events monitoring*: this to check whether you may have experienced any side effects that may be due to the study medication.

Your last visit will be at 90 days from the start of the trial. Samples will be collected and you will be reviewed by the team to check for any side effects. You will have the opportunity to ask questions to the team at all visits throughout the duration of the study and if you have any concerns outside of these visits you will be told who to contact.

Thank you for taking the time to read this information. If there is any other information you would like, please do not hesitate to contact us (020 3299 9000 ext. 32504).

Dr C Woodhouse, Dr V. C. Patel, Professor A Sanchez-Fueyo, Dr D. Shawcross
Institute of Liver Studies, Kings College Hospital

For peer review only

For peer review only - http://bmjopen.bmj.com/site/about/guidelines.xhtml

Faecal Microbiota Transplant (FMT) for recurrent or refractory *Clostridium difficile* Infection (CDI)

Donor screening questionnaire – Part 1

Name:

DOB:

Hospital number:

NHS number:

Contact details (preferably mobile):

Date of assessment:

Name / position of assessor:

Donor type	☐ Named donor<br>☐ Anonymous donor
If Named donor, name and hospital number of recipient	
If Named donor, relationship to recipient	
If Named donor, does the recipient normally live in the same dwelling?	☐ Yes ☐ No
Age	Exclude if <18 or >60
Gender	☐ Male ☐ Female
Ethnicity	☐ White - White British<br>☐ White - White Irish<br>☐ White - Other<br>☐ Mixed race – White and Black Caribbean<br>☐ Mixed race – White and Black African<br>☐ Mixed race – White and Asian<br>☐ Mixed race – Other<br>☐ Asian or Asian British – Indian<br>☐ Asian or Asian British – Bangladeshi<br>☐ Asian or Asian British – Pakistani<br>☐ Asian or Asian British – Other<br>☐ Black or Black British – Caribbean<br>☐ Black or Black British – African<br>☐ Black or Black British – Other<br>☐ Chinese<br>☐ Other
Height cm	

For peer review only

Weight kg	
BMI	Exclude if BMI>25
Has your weight changed by more than 5lb / 2kg in the past 6 months?	☐ Yes ☐ No<br><br>Detail:
Describe your diet (as many as apply):	☐ Omnivore<br>☐ Vegetarian<br>☐ Vegan<br>☐ Kosher<br>☐ Halal<br>☐ Raw food only<br>☐ Pescatarian<br>☐ No red meat<br>☐ Low carbohydrate<br>☐ Lactose free<br>☐ Gluten free<br>☐ Other
How many portions of fruit and vegetables do you consume per day?	☐ one or less<br>☐ two to three<br>☐ three to four<br>☐ five to six<br>☐ seven or more
How many servings of cow, sheep or goats milk do you consume per day?	☐ one or less<br>☐ two to three<br>☐ three to four<br>☐ five to six<br>☐ seven or more
Alcohol – units/week	
Smoking/day	
Normal bowel habit – average Bristol Stool Consistency	☐ 1<br>☐ 2<br>☐ 3<br>☐ 4<br>☐ 5<br>☐ 6<br>☐ 7<br><br>Exclude if 6 or 7
Normal bowel habit – average frequency	☐ >2/day<br>☐ once to twice daily<br>☐ once / 2 days<br>☐ <once / 2 days<br><br>Exclude if active diarrhoea (>3 UBM/day for at least 2 consecutive days)

For peer review only

During the past 7 days how many days were you physically active for a cumulative total of >60 mins/day?	
Have you ever been rejected as a blood donor/told not to donate?<br>If yes, why?	☐ Yes ☐ No<br>Exclude if YES
What is your country of birth?	
Have you ever resided in another country (other than UK) for >5 years?<br>If so which countries and when?	☐ Yes ☐ No
Do you currently have a profession that is associated with an increased risk of blood-borne transmissible diseases (e.g. daily contact with patients/inmates)?<br>If yes, what profession?	☐ Yes ☐ No<br>Exclude if health/social care worker with direct patient contact
Have you ever had a needle-stick or injury from a blood contaminated object from someone else? If yes, when and how was this follow up?	☐ Yes ☐ No
Have you ever injected yourself or been injected with illegal or non-prescribed drugs including body building drugs or cosmetics (even if this was only once or a long time ago?)	☐ Yes ☐ No<br>Exclude if YES
Have you ever had a tattoo?<br>If yes, when and in which country was it performed?	☐ Yes ☐ No<br>Exclude if within past 4 months
Have you ever had a piercing?<br>If yes, when and in which country was it performed?	☐ Yes ☐ No<br>Exclude if within past 4 months
Have you ever had acupuncture?<br>If yes, when and in which country was it performed?	☐ Yes ☐ No<br>Exclude if within past 4 months
Have you ever had an operation or undergone clinical treatment in a hospital with poor hygienic conditions?<br>If yes, when and in which country was it performed?	☐ Yes ☐ No
Have you ever had a rare infectious disease (e.g. tuberculosis, trypanosomiasis)?<br>If yes, when and which disease?	☐ Yes ☐ No
Have you ever been vaccinated against Hepatitis A or B?<br>If yes, which?	☐ Yes ☐ No

For peer review only

Have you received a live vaccine in the past 6 months?	☐ Yes ☐ No
In the last 12 months have you had sex with anyone who is HIV positive?	☐ Yes ☐ No<br>Exclude if Yes
In the last 12 months have you had sex with anyone with hepatitis B, hepatitis C or HTLV?	☐ Yes ☐ No<br>Exclude if Yes
In the last 12 months have you had sex with anyone who has ever been given money or drugs for sex?	☐ Yes ☐ No<br>Exclude if Yes
In the last 12 months have you had sex with anyone who has ever injected drugs?	☐ Yes ☐ No<br>Exclude if Yes
In the last 12 months have you had sex with anyone who may ever have had sex in parts of the world where HIV/AIDS is very common (this includes most countries in Africa)?	☐ Yes ☐ No<br>Exclude if Yes
**Male donors ONLY:**<br>In the last 12 months have you ever had oral or anal sex with a man, with or without a condom?	☐ Yes ☐ No<br>Exclude if Yes
**Female donors ONLY:**<br>In the last 12 months have you had sex with a man who has ever had oral or anal sex with another man, with or without a condom?	☐ Yes ☐ No<br>Exclude if Yes
Have you ever been treated for an intestinal infection?<br>If yes, which one and when?	☐ Yes ☐ No
Do you have any intestinal conditions:<br>Barrett's Oesophagus<br>Coeliac disease<br>Diverticular disease<br>Dyspepsia<br>Gastric ulcer<br>Gastro-oesophageal reflux disease<br>Hepatitis<br>H. pylori infection<br>Crohns disease<br>Ulcerative colitis<br>Other inflammatory bowel diseases<br>Irritable Bowel Syndrome<br>Lactose intolerance<br>Liver disease<br>Pancreatitis<br>Gastrointestinal malignancy or polyps	 ☐ Yes ☐ No<br>☐ Yes ☐ No<br>☐ Yes ☐ No<br>☐ Yes ☐ No<br>☐ Yes ☐ No<br>☐ Yes ☐ No<br>☐ Yes ☐ No<br>☐ Yes ☐ No<br>☐ Yes ☐ No<br>☐ Yes ☐ No<br>☐ Yes ☐ No<br>☐ Yes ☐ No<br>☐ Yes ☐ No<br>☐ Yes ☐ No<br>☐ Yes ☐ No<br>☐ Yes ☐ No<br><br>Exclude if Inflammatory Bowel Disease, Irritable Bowel Syndrome, GI malignancy, Hepatitis A, B, C or E

For peer review only

Have you used any antibiotics in the last 3 months?	☐ Yes ☐ No<br><br>Exclude if Yes
Have you had a fever in the last 2 weeks?	☐ Yes ☐ No<br><br>Exclude if Yes
Have you ever been incarcerated in prison?	☐ Yes ☐ No<br><br>Exclude if in past 4 months
Have you ever been immunosuppressed (e.g. during treatment for cancer, or for a solid organ transplant)?<br>If yes, when and why?	☐ Yes ☐ No
Have you ever had major gastrointestinal surgery?<br>If yes, when and why?	☐ Yes ☐ No<br><br>Relative exclusion criteria
Have you ever suffered from metabolic syndrome?	☐ Yes ☐ No<br><br>Relative exclusion criteria
Have you ever suffered from any autoimmune condition (e.g. rheumatoid), asthma or eczema?<br>If yes, which, and do you take any treatment?	☐ Yes ☐ No<br><br>Relative exclusion criteria
Have you ever had any chronic pain or fatigue syndromes?<br>If yes, which?	☐ Yes ☐ No<br><br>Relative exclusion criteria
Have you any history of CJD or other prion disease in your family? If yes, please specify<br>Patients should be considered to be at risk from genetic forms of CJD if they have or have had<br>1. Genetic testing, which has indicated they are at significant risk of developing CJD or other prion disease<br>2. A blood relative known to have a genetic mutation indicative of genetic CJD or other prion disease<br>3. Two or more blood relatives affected by CJD or other prion disease	☐ Yes ☐ No
Have you ever received growth hormone or gonadotrophic treatment? If yes, please specify;<br>i) Whether the hormone was derived from human pituitary glands<br>ii) The year of the treatment<br>iii) Whether the treatment was received in the UK or in another country	☐ Yes ☐ No

For peer review only

Recipients of hormone derived from human pituitary glands eg growth hormone or gonadtotrophin have been identified as potentially at risk of CJD. In the UK, the use of human growth hormone was stopped in 1985 but human-derived products may have been continued to be used in other countries. In the UK, the use of human-derived gonadotrophin was discontinued in 1973 but may have been continued in other countries after this time.	
Have you ever had surgery on your brain or spinal cord?<br>People who underwent intradural neurosurgical or spinal procedures before August 1992 may have received a graft of human-derived dura mater and should be treated as at increased risk, unless evidence can be provided that human-derived dura mater was not used. Patients who received a graft of human-derived dura mater between 1980 and August 1992 are at increased risk of both sporadic CJD and vCJD.	☐ Yes ☐ No
Are you normally resident in the UK?<br>If No state country of usual residence	☐ Yes ☐ No
Which countries have you visited in the last 12 months and what was the duration of stay?	
In the past 12 months have you been admitted to a hospital in a country other than the UK?<br><br>If yes state when and which countries	☐ Yes ☐ No
In the past 12 months have you been admitted to another hospital in London or Manchester?<br><br>If yes state when and which hospitals	☐ Yes ☐ No
List all current medications:	

For peer review only

	Exclude if any regular prescribed drugs (except OCP)

Is patient eligible to donate?	☐ Yes ☐ No
If No document reasons:	
If Yes proceed to screening tests<br>Ensure consent for testing documented	
Sign and date (health care practitioner) to indicate patient has provided consent	

Obtain serology for:	Requested?
HIV antigen/antibody	☐ Yes
HTLV-1/HTLV-II antibodies	☐ Yes
Hepatitis A IgG (add on IgM if positive)	☐ Yes
Hepatitis B surface antigen	☐ Yes
Hepatitis C IgG	☐ Yes
Syphilis serology (T. pallidum antibodies)	☐ Yes
CMV IgG (add on IgM if positive)	☐ Yes
EBV Serology	☐ Yes
Strongyloides serology	☐ Yes

Provide stool sample collection pots and request forms x3 for patient to take home. Instruct patient to provide three separate samples over 2-3 days, ensuring the pot is filled halfway in order to complete all testing.

Stool testing for:	Requested?
Bacterial culture (Campylobacter, Salmonella, Shigella, E. coli O:157) x3	☐ Yes
Ova, Cysts and Parasites x3	☐ Yes
C. difficile	☐ Yes
Resistant Gram negative organism screen	☐ Yes
MRSA screen	☐ Yes
Helicobacter pylori stool antigen	☐ Yes
E. histolytica PCR (not orderable on PCR)	

Arrange follow up visit for a minimum of 2-3 weeks time

Follow-up visit

Serology results:	Result
HIV antigen/antibody	☐ Detected ☐ Not detected

For peer review only

HTLV-1/HTLV-II antibodies	☐ Detected ☐ Not detected
Hepatitis A IgG (add on IgM if positive)	☐ Detected ☐ Not detected
Hepatitis B surface antigen	☐ Detected ☐ Not detected
Hepatitis C IgG	☐ Detected ☐ Not detected
Syphilis serology (T. pallidum antibodies)	☐ Detected ☐ Not detected
CMV IgG (add on IgM if positive)	☐ Detected ☐ Not detected
EBV Serology	☐ Detected ☐ Not detected
Strongyloides serology	☐ Detected ☐ Not detected

Stool results:	Result
Bacterial culture (Campylobacter, Salmonella, Shigella, E. coli O:157) x3	☐ Detected ☐ Not detected
Ova, Cysts and Parasites x3	☐ Detected ☐ Not detected
C. difficile	☐ Detected ☐ Not detected
Resistant Gram negative organism screen	☐ Detected ☐ Not detected
MRSA screen	☐ Detected ☐ Not detected
Helicobacter pylori stool antigen	☐ Detected ☐ Not detected
E. histolytica PCR (not orderable on PCR)	☐ Detected ☐ Not detected

Is all testing complete?	☐ Yes ☐ No
Accept as donor?	☐ Yes ☐ No
Any referrals required	☐ Yes ☐ No
Details of referrals made:	

Provide donor with stool collection kit x2 (contains collection instructions and copy of patient screening questionnaire 2). Explain requirement for stool to be fully processed within 6 hours of production.

The donor may provide multiple donations, but screening questionnaire 2 **MUST** be completed with **EACH** donation

If yes to any question, must undergo repeat full screening (however samples may be banked and quarantined for release after repeat testing).

If No to all questions samples may be released immediately and donor may continue to provide unlimited donations for 3 months after which time repeated screening will be required.

For peer review only

STANDARD PROTOCOL ITEMS: RECOMMENDATIONS FOR INTERVENTIONAL TRIALS

SPIRIT 2013 Checklist: Recommended items to address in a clinical trial protocol and related documents*

Section/item	Item No	Description
**Administrative information**		
Title	1	Descriptive title identifying the study design, population, interventions, and, if applicable, trial acronym (Page 1)
Trial registration	2a	Trial identifier and registry name. If not yet registered, name of intended registry (Page 1)
	2b	All items from the World Health Organization Trial Registration Data Set
Protocol version	3	Date and version identifier (Page 1)
Funding	4	Sources and types of financial, material, and other support (Pages 9, 47)
Roles and responsibilities	5a	Names, affiliations, and roles of protocol contributors (Pages 2-4)
	5b	Name and contact information for the trial sponsor (Pages 3-4)
	5c	Role of study sponsor and funders, if any, in study design; collection, management, analysis, and interpretation of data; writing of the report; and the decision to submit the report for publication, including whether they will have ultimate authority over any of these activities N/A
	5d	Composition, roles, and responsibilities of the coordinating centre, steering committee, endpoint adjudication committee, data management team, and other individuals or groups overseeing the trial, if applicable (see Item 21a for data monitoring committee) (Page 47)
**Introduction**		
Background and rationale	6a	Description of research question and justification for undertaking the trial, including summary of relevant studies (published and unpublished) examining benefits and harms for each intervention (Pages 17-20)
	6b	Explanation for choice of comparators (Pages 17-20)
Objectives	7	Specific objectives or hypotheses (Pages 21-22)

For peer review only

Trial design	8	Description of trial design including type of trial (eg, parallel group, crossover, factorial, single group), allocation ratio, and framework (eg, superiority, equivalence, noninferiority, exploratory) (Pages 23-25)

Methods: Participants, interventions, and outcomes

Study setting	9	Description of study settings (eg, community clinic, academic hospital) and list of countries where data will be collected. Reference to where list of study sites can be obtained (Page 23)
Eligibility criteria	10	Inclusion and exclusion criteria for participants. If applicable, eligibility criteria for study centres and individuals who will perform the interventions (eg, surgeons, psychotherapists) (Page 12, Page 29)
Interventions	11a	Interventions for each group with sufficient detail to allow replication, including how and when they will be administered (Pages 32-36)
	11b	Criteria for discontinuing or modifying allocated interventions for a given trial participant (eg, drug dose change in response to harms, participant request, or improving/worsening disease) (Pages 15-16, 36-39)
	11c	Strategies to improve adherence to intervention protocols, and any procedures for monitoring adherence (eg, drug tablet return, laboratory tests) (N/A, single treatment administered by clinical trials team)
	11d	Relevant concomitant care and interventions that are permitted or prohibited during the trial (Pages 29-31)
Outcomes	12	Primary, secondary, and other outcomes, including the specific measurement variable (eg, systolic blood pressure), analysis metric (eg, change from baseline, final value, time to event), method of aggregation (eg, median, proportion), and time point for each outcome. Explanation of the clinical relevance of chosen efficacy and harm outcomes is strongly recommended (Pages 10-11, 21-22)
Participant timeline	13	Time schedule of enrolment, interventions (including any run-ins and washouts), assessments, and visits for participants. A schematic diagram is highly recommended (see Figure) (Pages 24-28)
Sample size	14	Estimated number of participants needed to achieve study objectives and how it was determined, including clinical and statistical assumptions supporting any sample size calculations (Pages 45-46)
Recruitment	15	Strategies for achieving adequate participant enrolment to reach target sample size (Pages 23, 25)

Methods: Assignment of interventions (for controlled trials)

Allocation:

Sequence generation	16a	Method of generating the allocation sequence (eg, computer-generated random numbers), and list of any factors for stratification. To reduce predictability of a random sequence, details of any planned restriction (eg, blocking) should be provided in a separate document that is unavailable to those who enrol participants or assign interventions (Page 31)
Allocation concealment mechanism	16b	Mechanism of implementing the allocation sequence (eg, central telephone; sequentially numbered, opaque, sealed envelopes), describing any steps to conceal the sequence until interventions are assigned (Page 31, single blinded so only the patient is blinded)
Implementation	16c	Who will generate the allocation sequence, who will enrol participants, and who will assign participants to interventions (Trials Nurse, Chief Investigator or Clinical Research Fellow using randomisation software)
Blinding (masking)	17a	Who will be blinded after assignment to interventions (eg, trial participants, care providers, outcome assessors, data analysts), and how (Patient is blinded to treatment allocation as is trial statistician, database constructed so as not to reveal treatment allocation)
	17b	If blinded, circumstances under which unblinding is permissible, and procedure for revealing a participant's allocated intervention during the trial (N/A)

Methods: Data collection, management, and analysis

Data collection methods	18a	Plans for assessment and collection of outcome, baseline, and other trial data, including any related processes to promote data quality (eg, duplicate measurements, training of assessors) and a description of study instruments (eg, questionnaires, laboratory tests) along with their reliability and validity, if known. Reference to where data collection forms can be found, if not in the protocol
	18b	Plans to promote participant retention and complete follow-up, including list of any outcome data to be collected for participants who discontinue or deviate from intervention protocols
Data management	19	Plans for data entry, coding, security, and storage, including any related processes to promote data quality (eg, double data entry; range checks for data values). Reference to where details of data management procedures can be found, if not in the protocol (Page 43)
Statistical methods	20a	Statistical methods for analysing primary and secondary outcomes. Reference to where other details of the statistical analysis plan can be found, if not in the protocol (Pages 45-46)
	20b	Methods for any additional analyses (eg, subgroup and adjusted analyses) (To be confirmed)

	20c	Definition of analysis population relating to protocol non-adherence (eg, as randomised analysis), and any statistical methods to handle missing data (eg, multiple imputation) (Pages 45-46)
**Methods: Monitoring**		
Data monitoring	21a	Composition of data monitoring committee (DMC); summary of its role and reporting structure; statement of whether it is independent from the sponsor and competing interests; and reference to where further details about its charter can be found, if not in the protocol. Alternatively, an explanation of why a DMC is not needed (Page 47)
	21b	Description of any interim analyses and stopping guidelines, including who will have access to these interim results and make the final decision to terminate the trial (Pages 39-40, 47)
Harms	22	Plans for collecting, assessing, reporting, and managing solicited and spontaneously reported adverse events and other unintended effects of trial interventions or trial conduct (Pages 36-38)
Auditing	23	Frequency and procedures for auditing trial conduct, if any, and whether the process will be independent from investigators and the sponsor (Pages 14, 43-44)
**Ethics and dissemination**		
Research ethics approval	24	Plans for seeking research ethics committee/institutional review board (REC/IRB) approval (Page 14) Research Ethics approval was given by the London South East Research Ethics committee (ref 17/LO/2081)
Protocol amendments	25	Plans for communicating important protocol modifications (eg, changes to eligibility criteria, outcomes, analyses) to relevant parties (eg, investigators, REC/IRBs, trial participants, trial registries, journals, regulators) Direct communication with REC and involvement of DMC and TSC as required
Consent or assent	26a	Who will obtain informed consent or assent from potential trial participants or authorised surrogates, and how (see Item 32) (Pages 14-16)
	26b	Additional consent provisions for collection and use of participant data and biological specimens in ancillary studies, if applicable (N/A)
Confidentiality	27	How personal information about potential and enrolled participants will be collected, shared, and maintained in order to protect confidentiality before, during, and after the trial (Pages 15, 23, 31, 43, 44)
Declaration of interests	28	Financial and other competing interests for principal investigators for the overall trial and each study site N/A

For peer review only

Access to data	29	Statement of who will have access to the final trial dataset, and disclosure of contractual agreements that limit such access for investigators (Page 43)
Ancillary and post-trial care	30	Provisions, if any, for ancillary and post-trial care, and for compensation to those who suffer harm from trial participation (Information in PIS)
Dissemination policy	31a	Plans for investigators and sponsor to communicate trial results to participants, healthcare professionals, the public, and other relevant groups (eg, via publication, reporting in results databases, or other data sharing arrangements), including any publication restrictions (Page 47)
	31b	Authorship eligibility guidelines and any intended use of professional writers N/A
	31c	Plans, if any, for granting public access to the full protocol, participant-level dataset, and statistical code (Page 47)
**Appendices**		
Informed consent materials	32	Model consent form and other related documentation given to participants and authorised surrogates
Biological specimens	33	Plans for collection, laboratory evaluation, and storage of biological specimens for genetic or molecular analysis in the current trial and for future use in ancillary studies, if applicable

*It is strongly recommended that this checklist be read in conjunction with the SPIRIT 2013 Explanation & Elaboration for important clarification on the items. Amendments to the protocol should be tracked and dated. The SPIRIT checklist is copyrighted by the SPIRIT Group under the Creative Commons "Attribution-NonCommercial-NoDerivs 3.0 Unported" license.

For peer review only
